# Systematic review of the efficacy of pharmacological and non-pharmacological interventions for improving quality of life of people with dementia

**DOI:** 10.1192/bjp.2025.11

**Published:** 2026-01

**Authors:** Dominic Luxton, Naomi Thorpe, Emily Crane, Molly Warne, Olivia Cornwall, Daniel El-Dalil, Joshua Matthews, Anto P. Rajkumar

**Affiliations:** Mental Health and Neurosciences Academic Unit, School of Medicine, University of Nottingham, Nottingham, UK; Library and Knowledge Services, Nottinghamshire Healthcare NHS Foundation Trust, Nottingham, UK; Department of Medicine for the Elderly, University Hospitals of Derby and Burton NHS Foundation Trust, Derby, UK; Royal Primary Care, Chesterfield Royal Hospital NHS Foundation Trust, Chesterfield, UK; Emergency Department, Chesterfield Royal Hospital NHS Foundation Trust, Chesterfield, UK; Intensive Therapy Unit, Nottingham University Hospitals NHS Trust, Nottingham, UK; Haematology Department, Nottingham University Hospitals NHS Trust, Nottingham, UK; Mental Health Services for Older People, Nottinghamshire Health Care NHS Foundation Trust, Nottingham, UK

**Keywords:** Dementia, quality of life, systematic review, meta-analysis, psychosocial intervention

## Abstract

**Background:**

People with dementia (PwD) and their carers often consider maintaining good quality of life (QoL) more important than improvements in cognition or other symptoms of dementia. There is a clinical need for identifying interventions that can improve QoL of PwD. There are currently no evidence-based guidelines to help clinicians, patients and policy makers to make informed decisions regarding QoL in dementia.

**Aims:**

To conduct the first comprehensive systematic review of all studies that investigated efficacy of any pharmacological or non-pharmacological intervention for improving QoL of PwD.

**Method:**

Our review team identified eligible studies by comprehensively searching nine databases. We completed quality assessment, extracted relevant data and performed GRADE assessment of eligible studies. We conducted meta-analyses when three or more studies investigated an intervention for improving QoL of PwD.

**Results:**

We screened 14 389 abstracts and included 324 eligible studies. Our meta-analysis confirmed level 1 evidence supporting the use of group cognitive stimulation therapy for improving QoL (standardised mean difference 0.25; *P* = 0.003) of PwD. Our narrative data synthesis revealed level 2 evidence supporting 42 non-pharmacological interventions, including those based on cognitive rehabilitation, reminiscence, occupational therapy, robots, exercise or music therapy. Current evidence supporting the use of any pharmacological intervention for improving QoL in dementia is limited.

**Conclusions:**

Current evidence highlights the importance of non-pharmacological interventions and multidisciplinary care for supporting QoL of PwD. QoL should be prioritised when agreeing care plans. Further research focusing on QoL outcomes and investigating combined pharmacological and non-pharmacological interventions is urgently needed.

The global prevalence of dementia is estimated to reach 131.5 million by 2050,^1^ and the annual worldwide cost of dementia may rise to $2 trillion by 2030.^1^ Older adults consistently cite quality of life (QoL) as more important than disease-specific outcomes,^2^ and dementia can substantially impair the QoL of people with dementia (PwD).^3^ The importance of identifying interventions that can improve the QoL of PwD is increasingly recognised. Dementia intervention trials have focused mainly on cognitive and neuropsychiatric outcomes.^4^ However, the number of dementia studies including QoL as one of their outcomes has increased recently. There is an urgent need for systematically reviewing available evidence for guiding evidence-informed clinical decision-making and policy development that are essential for improving QoL in dementia.^5^

The World Health Organization defines QoL as ‘an individual’s perception of their position in life in the context of the culture and value systems in which they live and in relation to their goals, expectations, standards and concerns’.^6^ The views of PwD are important when assessing QoL, so widely used dementia-specific QoL measures such as the Quality of Life in Alzheimer’s Disease (QoL-AD)^7^ and the Dementia QoL measure (DEMQOL)^8^ are designed to be rated by both PwD and their carers. However, the results of self-reported and proxy-reported QoL measures may vary substantially.^9^ The concerns over reliability of self-rated QoL measures in those with severe cognitive impairment^10^ have been addressed by QoL assessment instruments such as the QoL for PwD (QUALIDEM).^3,11^ Moreover, assessing QoL in dementia is inherently subjective, and there is still a lack of consensus on which factors should be considered when measuring QoL of PwD.^3^ Hence, a wide range of heterogenous tools have been developed for measuring QoL in dementia, and their results are often difficult to compare.

Two small reviews have assessed the efficacy of pharmacological^12^ and non-pharmacological^13^ interventions for improving QoL of PwD separately. They did not include non-randomised studies or studies that investigated combined pharmacological and non-pharmacological interventions. Only 35 studies that were published before 2012 were included in those two reviews in total. More than 250 relevant studies on this topic have been published within the past 12 years. The prior review on non-pharmacological interventions^13^ reported group cognitive stimulation therapy (CST) as the only effective intervention for improving QoL of PwD living in care homes. It included only three CST studies and it could not conduct a meta-analysis. At least 20 studies that investigated the efficacy of CST in PwD have been published since then. Moreover, there has been a growing influence of technology in dementia care, and the recent studies have examined the use of innovative technologies such as companion robots^14^ and telemedicine^15^ for improving QoL of PwD. Furthermore, neuromodulation and alternative medicine interventions have not been included in any review.^13^

Hence, we aim to conduct the first comprehensive systematic review combining evidence from all studies that investigated any pharmacological, non-pharmacological and combined interventions for improving QoL of PwD. We do not have any evidence-based recommendations for improving QoL in dementia at present. Conducting a comprehensive systematic review is an essential prerequisite for facilitating the development of guidelines that may help clinicians, patients, their families and policy makers to make informed decisions regarding QoL in dementia.

## Method

### Protocol

Our systematic review protocol has been registered with the International Prospective Register of Systematic Reviews (PROSPERO identifier CRD42021249446; https://www.crd.york.ac.uk/prospero/display_record.php?RecordID=249446). We have documented all protocol amendments in the PROSPERO database. Supplementary File 1 presents the Preferred Reporting Items for Systematic Review and Meta-Analysis (PRISMA 2020) checklist.

### Search strategy

The following databases were searched initially from inception to 12 April 2021 by an information specialist (N.T.): Medline All, EMBASE, PsycINFO (all via Ovid), CINAHL Plus (EBSCOhost), Scopus, Web of Science Core Collection, Cochrane Library, Google Scholar and OpenGrey. We performed another round of systematic search of the nine databases for identifying eligible studies that were published until 20 June 2024. Our searches were restricted to papers available in English, and animal studies were excluded. The search strategy used a combination of free-text terms and relevant controlled vocabulary headings customised for each database, as well as advanced search syntax (truncation, Boolean logic AND/OR, and proximity searching) to ensure all relevant studies were identified. The search terms included the following themes, with synonyms to describe each: dementia, intervention and quality of life. Further details are presented in Supplementary File 2.

### Eligibility criteria

All original research papers that evaluated the efficacy of at least one pharmacological or non-pharmacological intervention in people with any type of clinically diagnosed dementia were deemed eligible to be included in this systematic review, if they included the changes in QoL as one of their primary or secondary outcome measures and employed any quantitative measure for reporting their QoL results. Randomised controlled trials (RCTs), quasi-RCTs, non-RCTs, case series and case reports that met our eligibility criteria were included. We excluded studies that were not published in English, cross-sectional studies that cannot evaluate the efficacy of any intervention, reviews and opinions. We excluded studies that included participants without dementia and did not report QoL results of PwD separately.

### Article selection

All identified abstracts were screened by a six-member review team using the Rayyan systematic review platform (Rayyan, Cambridge, MA, USA; see https://www.rayyan.ai/).^16^ Interrater agreement within the review team was good (multiple rater kappa = 0.78; *Z* = 36.22; *P* < 0.0001). An independent reviewer (N.T.) screened 10% of the abstracts again and confirmed the accuracy of the article selection process. We retrieved full texts of the potentially eligible abstracts, and their eligibility was assessed by a five-member review team. When the full text of a potentially eligible abstract was not retrievable, we requested the full text from the corresponding author by email. If the corresponding author did not respond within 14 days, then the abstract was excluded. Whenever there was disagreement regarding the eligibility of a study, the senior author (A.P.R.) independently reviewed it and resolved the disagreement through discussion with the review team. After we identified all eligible studies from our database searches, we employed backward citation analysis for identifying additional studies that met our eligibility criteria.

### Quality assessment

Two reviewers (D.L. and E.C.) assessed the quality of all eligible studies by using the Quality Assessment Tool for Quantitative Studies.^17^ Disagreements were resolved by discussion with the senior author (A.P.R.). Each study was assessed using the following domains: study design, selection bias, confounders, blinding, data collection method, withdrawals and drop-outs. Each domain was rated as strong, moderate or weak. The quality of included case reports and case series were assessed with the help of the CARE case report guidelines.^18^ Quality concerns were highlighted. We did not exclude any eligible study because of its quality assessment score.

### Data extraction

A three-member review team (D.L., E.C. and M.W.) started extracting the following data from all included studies from the first round of the systematic review on 29 June 2022, and from the second round of the systematic review on 15 August 2024: year of publication, investigated intervention(s), study design, setting, length of follow-up, prospective or retrospective study, sample size in each study arm, types and severity of dementia, mean age, gender and ethnicity of participants, QoL measure, statistical tests, reported *P*-values, effect sizes, mean difference between the study groups and their 95% confidence interval, multiple testing correction, correction(s) for confounder(s), interventions in comparison group, other concurrent treatments, type of qualitative data (if available) and study findings.

### Data synthesis

Narrative synthesis was carried out using extracted data. We categorised investigated interventions into pharmacological, non-pharmacological and combined interventions, and organised the data under these headings. We deemed the study findings as statistically significant when reported *P*-values were <0.05. We established hierarchies of evidence by using the Oxford Centre for Evidence-Based Medicine (OCEBM) levels of evidence, version 2.1.^19^ We then assessed the certainty of evidence by using the Grading of Recommendations, Assessment, Development and Evaluations (GRADE) framework certainty ratings,^20^ and those with higher ratings were given precedence when interpreting the results. When three or more studies had investigated the efficacy of an intervention for improving overall QoL or a specific QoL domain, we conducted appropriate meta-analyses. Then, we synthesised the information into summary tables according to the types of intervention and their levels of evidence.

### Data analysis

We used descriptive statistics to summarise extracted data. We assessed multiple rater interrater reliability with Stata version 16.1 for Windows (StataCorp LLC, College Station, TX, USA; see https://www.stata.com/) and its ‘kap’ command. We conducted meta-analyses with Stata version 16.1 and its ‘metan’ command. We assessed the degree of heterogeneity with Higgin’s *I* ^2^-statistic, and evaluated publication bias by using funnel plots. Standardised mean differences (SMDs) were used to synthesise continuous data.

## Results

**Fig. 1:**
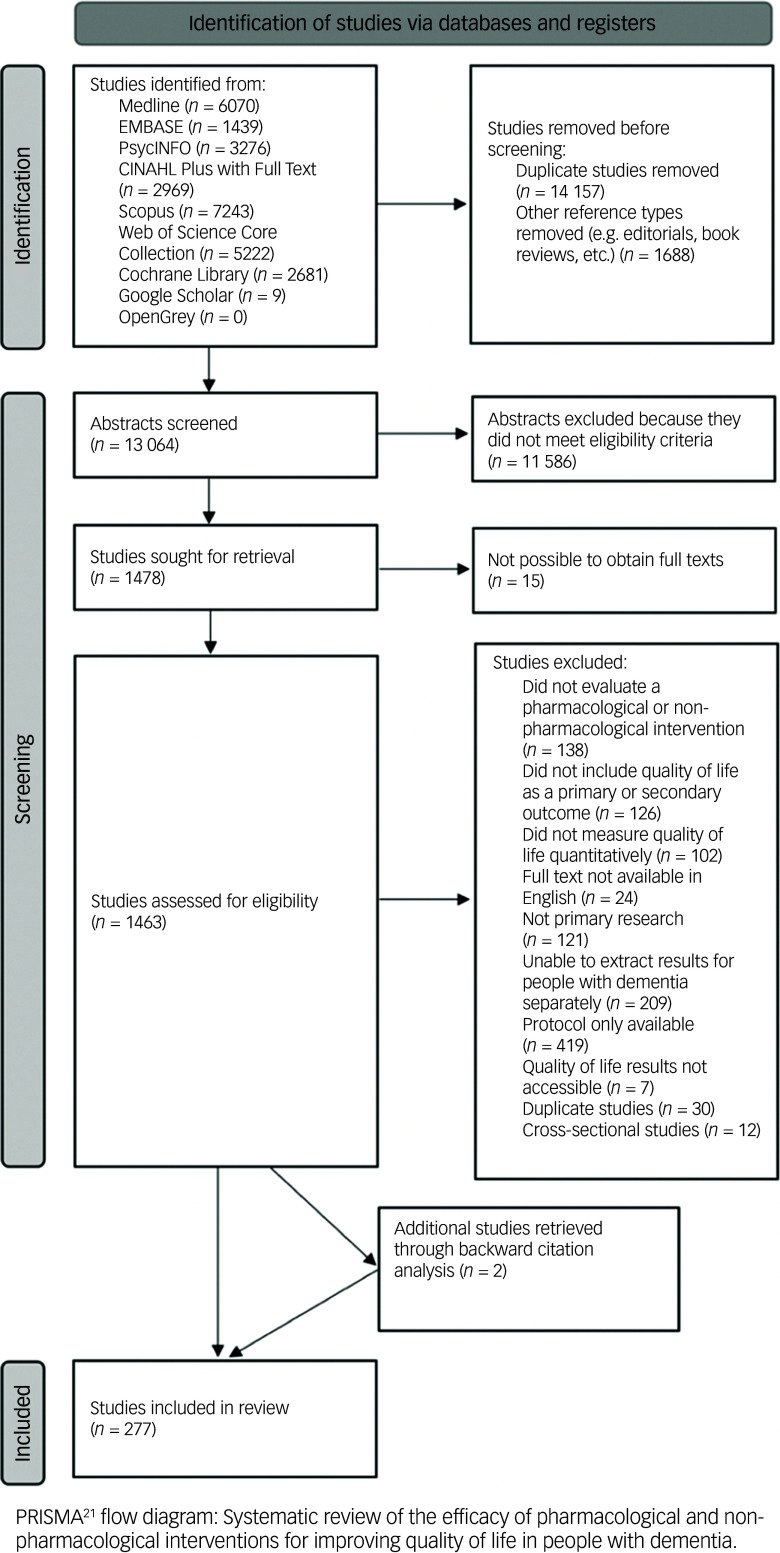
The Preferred Reporting Items for Systematic Reviews and Meta-Analyses (PRISMA) flowchart.

Figure 1 shows the PRISMA flow diagram presenting the article selection process of the first round of the systematic review.^21^ We screened 13 064 studies and identified 277 original research studies that met our eligibility criteria. Supplementary File 3 lists all 277 included studies. We screened 1325 studies and identified 47 more eligible studies in the second round of the systematic review. Supplementary File 4 lists those 47 included studies. A total of 54 different instruments were used to assess QoL of PwD. The most widely used instrument was the QoL-AD,^7,22^ which was used in 164 included studies. The DEMQOL and/or DEMQOL-Proxy^23^ were used in 34 studies, and the EuroQoL-5D (EQ-5D)^24^ was used in 33 included studies. The QUALIDEM,^11^ Quality of Life in Late-Stage Dementia Scale (QUALID)^25^ and Alzheimer’s Disease-Related Quality of Life (ADRQL)^26^ were used in 20, 16 and ten studies, respectively. Mean duration of follow-up in the included studies was 24.8 weeks, and the longest reported follow-up was 4 years. Supplementary Files 5, 5a and 6 present our quality assessment findings.

**Fig. 2:**
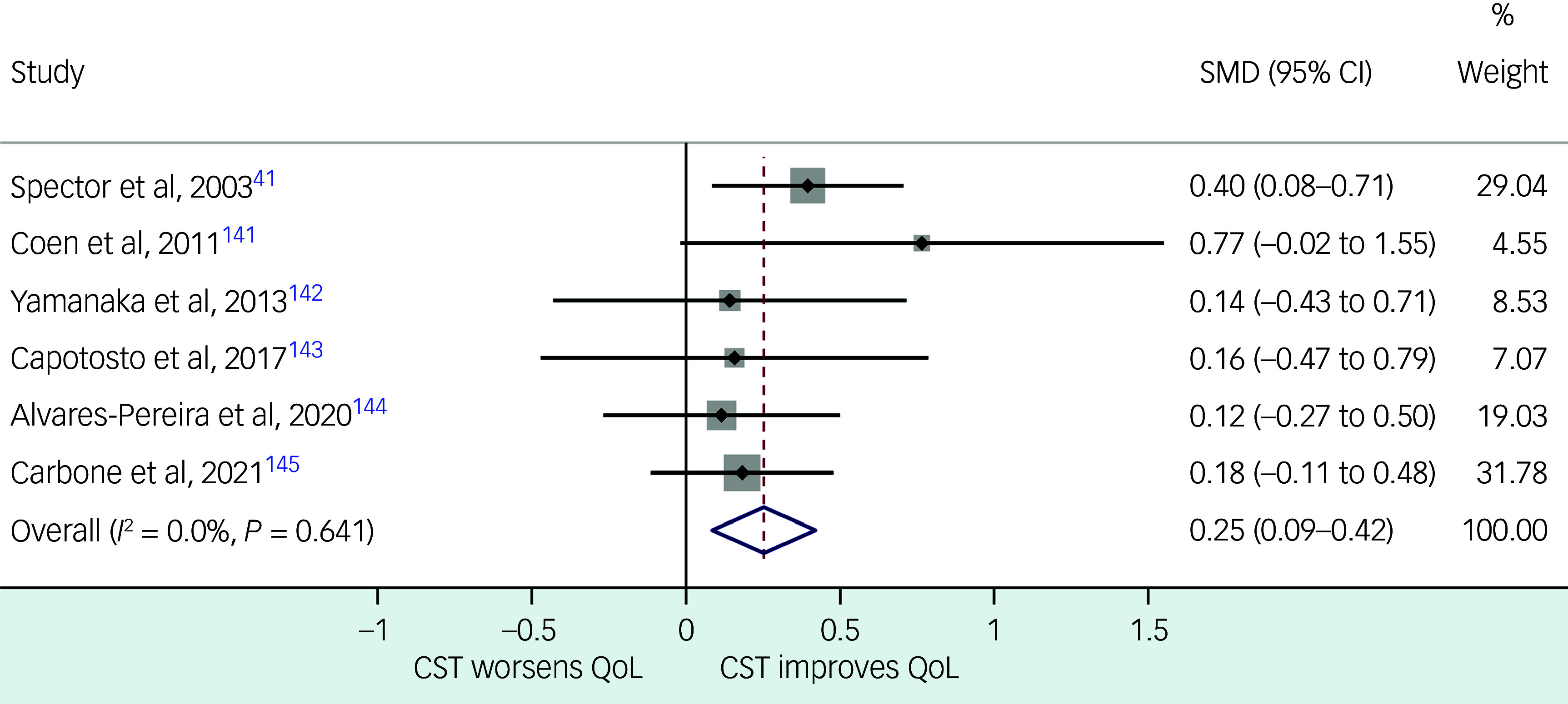
Fixed-effects meta-analysis of studies that investigated the efficacy of cognitive stimulation therapy for improving the quality of life of people with dementia. CST, cognitive stimulation therapy; QoL, quality of life; SMD, standardised mean difference.

### Pharmacological interventions

Table 1 summarises the current evidence supporting the use of various pharmacological interventions either on their own or combined with non-pharmacological interventions for improving QoL of PwD. None of them have level 1 evidence supporting their use for improving QoL.

**Table 1:** Pharmacological or combined pharmacological and non-pharmacological interventions for improving quality of life in people with dementia

Intervention	Highest level of evidence	GRADE certainty rating
Rasagiline^27^	2	Moderate
Plasma exchange with albumin replacement^a^	2	Moderate
Oxiracetam^28^	2	Low
Testosterone^29^	2	Low
Donepezil plus hyperbaric oxygen therapy^30^	3	Low
Donepezil plus complex psychosocial intervention^31^	3	Low
Memantine plus scalp electroacupuncture^32^	3	Low
Rivastigmine and donepezil combined therapy^33^	3	Low
Nimodipine^34^	3	Very low
Donepezil and memantine combined therapy^35^	4	Moderate
Rivastigmine^36^	4	Moderate
Galantamine^37^	4	Low
Memantine^38^	4	Low
Olanzapine^39^	4	Low
Risperidone^39^	4	Low
Dextromethorphan and quinidine^b^	4	Low
Out-patient dementia service combined with acetylcholinesterase (AChE) inhibitor use^40^	4	Very low

Oxford Centre for Evidence-Based Medicine levels of evidence^19^: 1 = systematic review of randomised trials; 2 = randomised trial or observational study with dramatic effect; 3 = non-randomised controlled cohort/follow-up study; 4 = case series, case–control studies or historically controlled studies; 5 = mechanism-based reasoning. GRADE of certainty ratings^20^: high = the true effect is similar to the estimated effect; moderate = the true effect is probably close to the estimated effect; low = the true effect may be markedly different from the estimated effect; very low = the true effect is probably markedly different from the estimated effect.

a Details of this study are available in Supplementary File 4.

b Details of this study are available in Supplementary File 3.

**Table 2:** Non-pharmacological interventions that have level 1 or level 2 evidence for improving quality of life of people with dementia

Intervention	Highest level of evidence	GRADE certainty rating
CST^41,42,a^	1	High
Activities of daily living training^43^	2	High
An adapted mindfulness intervention^44^	2	High
Caregiver delivered multisensory cognitive stimulation intervention^b^	2	High
Collaborative dementia care programme delivered via telephone and internet^45^	2	High
Community occupational therapy programme^46^	2	High
Continuous aerobic training^47^	2	High
Dementia care mapping^48^	2	High
Digital adaption of person-centred nursing home training programme^b^	2	High
Global postural re-education^49,50^	2	High
Individual reminiscence therapy^51^	2	High
Maintenance CST^52^	2	High
Novel occupational therapy programme^53^	2	High
Person-centred residential care^a^	2	High
Person-centred residential environment^a^	2	High
Group reminiscence therapy^54^	2	High
Stressor-oriented multicomponent intervention^55^	2	High
Activity scheduling^56^	2	Moderate
Arithmetic and combined drawing and writing interventions^b^	2	Moderate
CST based on Roy’s adaptation model^57^	2	Moderate
Creative expression therapy^58^	2	Moderate
Dementia Related Manual for Sleep; Strategies for Relatives Intervention (DREAMS START)^59^	2	Moderate
Hearing aids^60^	2	Moderate
Modified ketogenic diet^61^	2	Moderate
Music listening group^62^	2	Moderate
Music therapy (singing group)^a^	2	Moderate
Observation of mastication videos^b^	2	Moderate
Predictive nursing care programme^a^	2	Moderate
Recollection-based occupational therapy^63^	2	Moderate
Reminiscence programme using the life story approach^64^	2	Moderate
Social robots^b^	2	Moderate
Tailored activity programme^65^	2	Moderate
Chair yoga^66^	2	Low
Cognitive–behavioural therapy-based intervention (the Peaceful Mind programme)^67^	2	Low
CST combined with a fall prevention exercise (CogEx)^68^	2	Low
Companion robots (PARO)^14^	2	Low
Continuing care combined with music therapy^a^	2	Low
Cycling intervention^69^	2	Low
Drama therapy^b^	2	Low
Goal-orientated cognitive rehabilitation programme^70^	2	Low
Hypnosis^71^	2	Low
Life review^72^	2	Low
Visual mapping assistive technology^b^	2	Low

Oxford Centre for Evidence-Based Medicine levels of evidence^19^: 1 = systematic review of randomised trials; 2 = randomised trial or observational study with dramatic effect; 3 = non-randomised controlled cohort/follow-up study; 4 = case series, case–control studies or historically controlled studies; 5 = mechanism-based reasoning. GRADE of certainty ratings^20^: high = the true effect is similar to the estimated effect; moderate = the true effect is probably close to the estimated effect; low = the true effect may be markedly different from the estimated effect; very low = the true effect is probably markedly different from the estimated effect. CST, cognitive stimulation therapy.

a Further details are available in Supplementary File 3.

b Further details are available in Supplementary File 4.

#### Acetylcholinesterase inhibitors

Fifteen studies investigated the efficacy of acetylcholinesterase inhibitors (AChEIs) for improving QoL of PwD. Eight studies, including four RCTs, investigated donepezil monotherapy. None found statistically significant evidence supporting its efficacy. There is weak evidence supporting donepezil in combination with either rivastigmine^33^ or memantine.^35^ Two studies, including an RCT, investigated rivastigmine monotherapy. The RCT did not find evidence to support the use of rivastigmine to improve QoL, but a before-and-after study reported statistically significant increases in QoL-AD scores after treatment with rivastigmine transdermal patches.^36^ Moreover, an RCT that compared galantamine and nimodipine combined treatment with galantamine and placebo in people with Alzheimer’s disease and cerebrovascular disease did not find significant difference in QoL between groups.^37^ However, when all galantamine-treated patients were analysed together, there was a statistically significant difference between before and after participant-rated QoL-AD scores. One RCT investigated high-dose tacrine,^73^ which has been discontinued because of hepatotoxicity risk.

#### Memantine

Five studies investigated memantine and one of them supported its efficacy. A before-and-after study^38^ supported the use of memantine in alcohol-related dementia by reporting statistically significant improvement in QoL scores at 12 weeks follow-up. An RCT that compared memantine with placebo in people with Parkinson’s disease dementia (PDD) did not find a statistically significant difference in DEMQOL scores.^74^ Another RCT investigating people with PDD or dementia with Lewy bodies (DLB) compared memantine with placebo, and did not find a statistically significant difference in QoL-AD scores.^75^ However, a retrospective medical records review reported significantly higher QoL scores in a combined donepezil plus memantine group compared with donepezil monotherapy when treating concomitant Alzheimer’s disease and chronic obstructive pulmonary disease.^35^

#### Antidepressants

Three studies investigated antidepressants, and none provided evidence for their efficacy for improving QoL. An RCT that investigated bupropion use in people with Alzheimer’s disease and apathy reported statistically significant improvement in QoL in the placebo group.^76^ Another RCT and a before-and-after trial investigated sertraline and venlafaxine, respectively, in people with Alzheimer’s disease and depression, and did not find a statistically significant improvement in QoL (Supplementary File 3).

#### Antipsychotics

Three studies investigated antipsychotic medications. The highest level of evidence available for olanzapine or risperidone monotherapy was level 4. A double-blind trial found statistically significant improvements in QoL scores in groups treated with olanzapine or risperidone, but the difference between groups was not statistically significant.^39^ Another RCT investigated quetiapine, risperidone and olanzapine, and none of them improved QoL at 36 weeks follow-up.^77^

#### Other pharmacological interventions

Eighteen studies investigated other pharmacological interventions, and five of them reported statistically significant improvement in QoL. A placebo-controlled RCT reported statistically significant improvements in QoL-AD scores in people with mild Alzheimer’s disease (Supplementary File 4). Another placebo-controlled RCT reported statistically significant improvement in QoL scores after treatment with rasagiline in Alzheimer’s disease.^27^ Another small RCT (*N* = 18) that investigated testosterone in people with Alzheimer’s disease reported statistically significant improvements in caregiver-rated QoL-AD scores.^29^ However, the participant-rated QoL scores did not differ significantly at follow-up. Moreover, an RCT that investigated oxiracetam in people with multi-infarct or mixed dementia reported statistically significant improvements in QoL over 12 weeks.^28^ A non-randomised trial comparing nimodipine monotherapy with combined nimodipine and piracetam therapy in people with vascular dementia reported statistically significant benefit in all QoL domains in the nimodipine monotherapy group.^34^ Furthermore, a large RCT (*N* = 1534) that compared treatment with semagacestat (a γ-secretase inhibitor) with placebo in PwD reported statistically significant worsening of QoL in the semagacestat group at 76 weeks.^78^

### Combined pharmacological and non-pharmacological interventions

Three out of five studies that investigated AChEIs in combination with non-pharmacological interventions reported statistically significant QoL improvements. A non-randomised trial found statistically significant QoL improvement in the group receiving donepezil and a complex psychosocial intervention including reminiscence therapy, when compared with donepezil monotherapy.^31^ Another non-randomised trial reported similar QoL improvements with donepezil combined with hyperbaric oxygen therapy, when compared with donepezil monotherapy, in people with PDD.^30^ Moreover, a before-and-after study reported statistically significant improvements in QoL-AD scores of people with Alzheimer’s disease, when AChEI use was combined with out-patient dementia service use.^40^ A retrospective cohort study in vascular dementia compared memantine therapy with and without scalp electroacupuncture.^32^ QoL improvements were statistically significant in both intervention groups when compared with a control group. However, another RCT did not find any evidence to support rivastigmine therapy combined with physical exercise for improving QoL.^79^

### Non-pharmacological interventions

Table 2 presents all non-pharmacological interventions that have level 1 or level 2 evidence supporting their use for improving QoL of PwD. Supplementary Files 7 and 8 list all non-pharmacological interventions that have level 3 or level 4 evidence, respectively, for improving QoL.

#### CST

CST is the most widely investigated non-pharmacological intervention, and it is the only intervention that has level 1 evidence for improving QoL of PwD.^41^ Manualised group CST has been reported to significantly improve the QoL of PwD.^41^ Our fixed-effects meta-analysis (Fig. 2; Supplementary File 9) combined data from six relevant RCTs, and confirmed level 1 evidence supporting the use of group CST (pooled SMD = 0.25; 95% CI 0.09–0.42; *P* = 0.003). The heterogeneity between these studies was not statistically significant (*I* ^2^ = 0.0%; *χ* ^2^ = 3.39; d.f. = 5; *P* = 0.64). We could not include ten more studies that investigated the group CST in PwD in our meta-analysis, because seven of them were single-group studies without controls, two of them did not report group-level changes in QoL scores and another study^42^ used data from the previous CST RCT participants.^41^

Seven RCTs investigated other versions of CST. An RCT investigated CST intervention based on Roy’s adaption model,^80^ and reported statistically significant improvement in QoL.^57^ Maintenance CST was investigated by two RCTs. One of them reported statistically significant benefits for self-rated QoL at the 6-month primary end-point,^52^ and the proxy-rated QoL scores were significantly higher in the maintenance CST group at 3 months follow-up. Another RCT did not find any effect on QoL following maintenance CST sessions.^42^ Individual CST was investigated by two RCTs, and both did not report significant differences in QoL between individual CST and control groups.^81,82^ RCTs investigating virtual and mobile app versions of CST did not report significant differences in QoL (Supplementary File 4). Moreover, other RCTs studied CST combined with carer-training programmes^83^ and fall prevention exercises,^68^ and neither reported statistically significant QoL improvements.

#### Physical exercise

Twenty-three studies, including 14 RCTs, investigated a wide range of exercise interventions. Two RCTs, which investigated cycling^69^ and a continuous aerobic training intervention,^47^ reported statistically significant favourable QoL outcomes. Another RCT investigated the effects of a 6-month activities of daily living programme and an exercise programme on QoL, and reported statistically significant improvement on overall QoL by the activities of daily living programme.^43^ Another intervention including aerobic walking and upper limb exercises reportedly led to significantly better QoL scores.^84^ Furthermore, a small RCT compared the effects of chair yoga, a music intervention and chair-based exercise for improving QoL of PwD.^66^ QoL-AD scores were significantly higher in the chair yoga group compared with the music intervention group at 12 weeks. A multicomponent exercise programme including aerobic activities, lower body strength training, exercises to improve balance and flexibility, and training caregivers significantly improved QoL of PwD over the 13-month study period.^85^

#### Reminiscence therapy

Fifteen studies investigated various reminiscence therapy-based interventions. Six reported statistically significant QoL improvements. There is level 2 evidence for reminiscence therapy and level 4 evidence for intergenerational reminiscence therapy. An RCT investigated an 8-week reminiscence therapy programme and reported statistically significant QoL improvement.^54^ Individual reminiscence therapy was investigated by two RCTs, and both supported its efficacy for improving QoL of PwD.^51,64^ Three before-and-after studies that investigated a computer interactive reminiscence and conversation aid intervention,^86^ a reminiscence therapy programme developed using programmes by Westerhof et al^87^ and Webster et al,^88^ and an intergenerational reminiscence therapy programme^89^ reported statistically significant differences in QoL scores. However, eight more studies that investigated other variations of reminiscence therapy did not find statistically significant changes.

#### Music therapy

Fifteen studies investigated music-based interventions. Six of them reported statistically significant positive QoL findings. Level 2 evidence was found for a group music listening intervention, a group singing intervention and a complex intervention consisting of dietary guidance, medication guidance, life guidance and music listening therapy (Supplementary File 3). The RCT that investigated the group music listening intervention, where participants were encouraged to discuss their emotions, thoughts and feelings evoked by the music, had the longest follow-up, and it reported statistically significant QoL improvements after 9 months.^62^ Moreover, a before-and-after study that investigated a video music therapy intervention showing traditional folk dances and folk music reported a statistically significant increase in QoL scores after 6 months.^90^

#### Cognitive rehabilitation

Thirteen studies investigated various cognitive rehabilitation-based programmes. Six of them reported statistically significant QoL improvements. There is level 2 evidence supporting the use of goal-orientated cognitive rehabilitation in people with Parkinson’s disease and people with either PDD or DLB. An RCT reported statistically significant QoL improvement in the goal-oriented cognitive rehabilitation group after 6 months.^70^ Four studies that investigated multicomponent cognitive rehabilitation programmes, including a non-randomised trial evaluating a multidisciplinary rehabilitation programme^91^ and a before-and-after study investigating a cognitive rehabilitation programme developed for community-dwelling PwD,^92^ found statistically significant QoL improvements. A non-randomised trial investigated a complex rehabilitation nursing programme including cognitive rehabilitation, and reported statistically significantly higher QoL-AD scores after 6 months.^93^

#### Occupational therapy

Eleven studies, including eight RCTs, investigated a variety of occupational therapy programmes, and four of them reported statistically significant QoL improvements. Two RCTs investigated community-based occupational therapy programmes. One of them examined a 5-week occupational therapy programme consisting of ten sessions, and reported statistically significantly improvements in Dementia Quality of Life Instrument (DQoL)^94^ scores at 6 and 12 weeks follow-up.^46^ The second RCT investigated another 5-week programme consisting of relaxation, personal activities, cognitive exercise and recreational activity,^53^ and found statistically significant improvement in physical and psychological domain QoL scores. Another small RCT (*N* = 35) that investigated a recollection-based occupational therapy intervention reported statistically significant QoL improvement in PwD.^63^ Similarly, an RCT that investigated the effects of activity scheduling reported statistically significant improvement in QoL after 12 weeks.^56^ However, a large RCT (*N* = 465) that investigated the Community Occupational Therapy Dementia UK version did not find QoL improvement.^95^ Similarly, an RCT that investigated a care home-based occupational therapy programme did not support its use after 12 weeks follow-up.^96^

#### Art therapy

Seven studies investigated art-based interventions, and three of them found statistically significant QoL improvements. A randomised waiting list controlled study that investigated an art museum-based intervention found statistically significant improvements in self-rated QoL.^97^ Two before-and-after studies that investigated a therapeutic visual art intervention^98^ and the effects of visits to an art gallery with an art educator^99^ reported significant improvement in QoL of PwD.

#### Robot-aided interventions

Seven studies investigated the use of robots. Two of them reported statistically significant QoL improvement. RCTs investigating the use of a companion robot (PARO) and a social robot reported a statistically significant positive influence on participants’ QoL.^14^ However, another RCT including a PARO robot group reported a statistically significant QoL decline in that group (Supplementary File 3). Two before-and-after studies that investigated robotic pet ownership^100^ and the use of a service companion robot^101^ reported QoL improvements that were not statistically significant.

#### Other technology-aided interventions

Five studies assessed the efficacy of other technologies for improving QoL of PwD, and two studies reported statistically significant improvements. A large RCT (*N* = 655) examined a programme (The Care Ecosystem) for collaborative dementia care delivered via telephone and internet, and it reported a statistically significant decline in the control group when compared with the intervention group.^45^ Furthermore, a small before-and-after study (*N* = 8) studied the effects of emisymmetric bilateral stimulation, a novel electromagnetic field brain stimulation, and found statistically significant improvements in both physical and mental QoL domains^102^ after 5 weeks follow-up.^103^ Studies that investigated a virtual reality-based training programme,^104^ a wearable camera used as an external memory aid,^105^ an exergaming intervention,^106^ vagus nerve stimulation^107^ and a video communication app delivering weekly health services^15^ did not find any evidence supporting their use for improving QoL of PwD.

#### Gardening

Five studies investigated gardening-based interventions. A before-and-after study that investigated the use of therapeutic gardening reported a statistically significant increase (12.8%) in mean QoL scores, and provided level 4 evidence supporting its use.^108^ Moreover, participants in a feasibility trial investigating daily garden use experienced statistically significant improvement in the QUALIDEM domain of negative affect after 2 weeks follow-up.^109^ However, the only RCT that investigated horticultural therapy for improving QoL of PwD did not find a statistically significant difference.^110^

#### Canine-assisted therapy

Four studies investigated the use of dogs for improving QoL of PwD. A before-and-after study that investigated personalised prescription of canine-assisted therapy reported significantly improved QoL scores 1 week after the intervention.^111^ An RCT examining a standardised canine-assisted therapy reported statistically significant QoL improvement in one facility and statistically significant QoL decline in another facility.^112^ Furthermore, a before-and-after study that investigated a once-weekly canine-therapy programme found statistically significant QoL decline after 12 months follow-up.^113^

#### Dementia care mapping

Dementia care mapping (DCM) is a person-centred multicomponent intervention that is based on Kitwood’s social psychological theory of personhood in dementia.^114^ Five studies, including three RCTs, that investigated the interventions based on the DCM model as a process (as opposed to the DCM tool measuring QoL) were included in this systematic review. A large RCT reported statistically significant QoL improvement following DCM in PwD living in nursing homes.^48^ However, two other RCTs did not find statistically significant differences in QoL scores.^115,116^

#### Life story

Three studies, including an RCT, investigated life story-based interventions. The RCT showed statistically significant QoL improvement in the ‘life review’ group after 12 weeks.^72^ The other two studies did not report statistically significant differences in QoL.^117,118^

#### Dance therapy

Five studies investigated dance-based interventions. Two of them reported statistically significant QoL improvement. A before-and-after study investigating a person-centred creative dance intervention reported statistically significant improvement in self-rated QoL after 8 weeks.^119^ Another small before-and-after study that investigated circle dancing reported QoL improvement in five out of seven participants with dementia.^120^

#### Acupuncture

Two studies investigated the effects of acupuncture. A small (*N* = 16) before-and-after study reported an increase in QoL scores in people with vascular dementia receiving acupuncture every other day over 6 weeks.^121^ However, a follow-up RCT of a similar intervention by the same authors did not find statistically significant QoL improvement after 10 weeks follow-up.^122^ A relatively large (*N* = 129) before-and-after study investigating acupressure reported statistically significant improvement in the Global Health Quality of Life^123^ mood subscale in PwD.^124^

#### Mindfulness

A small RCT and a before-and-after study investigated mindfulness-based interventions. The RCT studied a group-based adapted mindfulness intervention for people with mild to moderate dementia living in care homes, and reported statistically significant improvement in QoL-AD scores, with medium effect size.^44^ The before-and-after study investigated another adapted mindfulness-based group training for PwD and their caregivers.^125^ It reported a reduction in QoL following the intervention.

#### Early psychosocial intervention

Two large RCTs investigated early psychosocial counselling and support programmes in people with Alzheimer’s dementia. The larger RCT (*N* = 330) reported better QoL after 12 months.^126^ However, both studies did not reveal any long-term effect of the interventions on QoL at 36 months follow-up (Supplementary File 3).

#### Global postural re-education

Two RCTs investigated global postural re-education, a physiotherapy method. Both RCTs reported statistically significant improvements in QoL of PwD.^49,50^

#### Other non-pharmacological interventions

A further 44 non-pharmacological interventions were investigated by a single study, which has not been replicated. Sixteen of them reported positive QoL outcomes in PwD. Among them, five studies investigated a heterogeneous range of complex non-pharmacological interventions. An RCT (*N* = 218) reported statistically significant improvement in QoL-AD scores by a stressor-oriented multicomponent intervention.^55^ A single-blind feasibility RCT investigating the Dementia Related Manual For Sleep: Strategies For Relatives Intervention (DREAMS START) reported statistically significant QoL improvement after 3 months.^59^ A small RCT investigated a tailored activity programme matching activities to the cognitive and functional capabilities, previous roles, habits and interests of the PwD.^65^ Caregivers reported statistically significant improvement in QoL of the PwD after 4 months follow-up. However, the study did not find such difference in the self-rated QoL scores. Supplementary File 10 presents the details of 19 studies, which reported statistically significant improvements in individual QoL domains, but not in overall QoL scores. Another small RCT investigated a cognitive–behavioural therapy based intervention, ‘Peaceful Mind’, and reported statistically significant improvements in QoL scores after 3 months, but this effect was not sustained at 6 months follow-up.^67^ Three more large RCTs investigating complex non-pharmacological interventions, a liaison input programme for delivering personalised intervention packages,^127^ a complex intervention including actively approaching counselling and caregiver support groups,^128^ and a human rights based intervention,^129^ did not find statistically significant QoL changes. Moreover, two before-and-after studies that investigated a programme combining caregiver training with residential respite stay^130^ and a staff training programme for assisted living residences^131^ reported a statistically significant decline in QoL.

An RCT (*N* = 72) reported a statistically significant improvement in QoL indices, measured by the DCM tool, in PwD receiving essential balm oil aromatherapy.^132^ An RCT investigating drama therapy has reported significant improvements in QUALID scores (Supplementary File 4). Another RCT found statistically significant improvement in QoL-AD scores in PwD receiving a ketogenic diet compared with those receiving normal diet.^61^ One more RCT compared creative expression therapy with standard cognitive training, and reported statistically significant QoL improvement after a month.^58^ Furthermore, a small RCT evaluated the interventions for improving QoL of people with Alzheimer’s dementia and age-related hearing loss, and reported significantly higher ADRQL scores at 12 months follow-up.^60^ Another small RCT (*N* = 18) investigated hypnosis and discussion therapy, and reported significantly better QoL of PwD receiving hypnosis after 21 months.^71^

## Discussion

This is the first comprehensive systematic review summarising available evidence from 324 relevant studies for the efficacy of all pharmacological, non-pharmacological and combined interventions for improving the QoL of PwD. Two prior small reviews focusing on either pharmacological or non-pharmacological interventions included only 15 studies^12^ and 20 studies,^13^ respectively. This systematic review advances our understanding of the existing evidence on this field comprehensively, and provides a clear building block upon which future research and clinical guidelines can be developed. It presents the first meta-analysis on this topic that confirms level 1 evidence supporting the efficacy of group CST^133^ for improving the QoL of PwD. Tables 1 and 2 list 46 interventions that currently have level 2 evidence supporting their clinical use. Nineteen more pharmacological and non-pharmacological interventions have level 3 evidence that warrant further research for refining their components and investigating their effectiveness.

Strengths of this systematic review are a comprehensive search of nine databases including grey literature, broad eligibility criteria, rigorous quality assessment, interrater reliability assessment and GRADE assessment of current evidence. We should acknowledge the limitations of excluding studies that were not published in English and studies that did not report QoL results of PwD separately. There was large heterogeneity among the included studies, because of the limited standardisation of various non-pharmacological interventions and the differences in population characteristics, dementia types, treatment settings, expertise of therapists, QoL definitions and QoL measurements. The degree of heterogeneity did not allow for conducting meaningful meta-analyses for synthesising available evidence for the efficacy of several non-pharmacological interventions, such as physical exercise, reminiscence therapy, cognitive rehabilitation and occupational therapy interventions. Moreover, important limitations of the included studies include small sample sizes, lack of power calculations, lack of appropriate control groups and short follow-up durations. Blinding is less feasible in non-pharmacological intervention trials than in pharmacological intervention trials. Lack of blinding might have led to more favourable reported QoL outcomes in included non-pharmacological intervention studies. Because of inconsistent reporting of rating procedures by many included studies, we could not ascertain whether reported QoL measures were rated by PwD and/or by their carers. Hence, we presented our findings without differentiating between self-rated and carer-rated QoL measures. Studies investigating specific types of dementia, especially non-Alzheimer’s dementia such as DLB or PDD, were sparse. Furthermore, there is still no consensus regarding how QoL should be measured in PwD, and the predictive validity of available QoL measurements remain uncertain.^3^ Self-rated QoL measures are often influenced by the severity of cognitive impairment, associated neuropsychiatric symptoms and comorbid physical illnesses. Carer-rated QoL measures can be influenced by the degree of functional impairment, caregiving burden and available support systems.^134,135^

The National Institute for Health and Care Excellence (NICE) guidelines do not currently support any pharmacological intervention for improving the QoL of PwD.^136^ Combined donepezil and memantine therapy, and monotherapy with rivastigmine, galantamine or memantine, have weak level 4 evidence for improving QoL of PwD and for continuing their routine clinical use. A few medications that can only be used with caution in PwD, such as antipsychotic medications, testosterone, rasagiline, nimodipine, dextromethorphan and quinidine, have similar level 4 or relatively better level 3 evidence for improving QoL. However, they do not have strong evidence for benefiting cognitive or neuropsychiatric symptoms of dementia, and there are safety concerns regarding their use in PwD. Hence, the current evidence do not support their routine use in PwD for improving their QoL. Combining non-pharmacological interventions with commonly used dementia medications can improve QoL outcomes. There is level 3 evidence supporting the combination of donepezil with complex psychosocial intervention,^31^ and memantine with scalp electroacupuncture,^32^ for improving the QoL of PwD. There is an urgent need for further research investigating more combinations of approved dementia medications, neuromodulation and standardised non-pharmacological interventions. Moreover, large dementia clinical trials investigating pharmacological interventions often neglect the importance of including QoL outcomes, and their follow-up periods may not be long enough to detect clinically significant QoL changes. Future clinical trials should consider these issues for identifying better evidence-based pharmacological or combined interventions for improving the QoL of PwD.

Current NICE guidelines for the management of dementia^136^ recommend offering group CST, and considering group reminiscence therapy, cognitive rehabilitation and occupational therapy for improving the well-being of people with mild to moderate to dementia. QoL including objective measures and physical factors is more comprehensive than subjective well-being,^137^ and the current NICE guidelines do not provide any recommendation regarding QoL of PwD. This systematic review and our meta-analysis confirm level 1 evidence for offering group CST for improving QoL for PwD, especially those with mild to moderate dementia. As the pooled SMD was small, there is a need for further investigations combining group CST with other non-pharmacological or pharmacological interventions. Additionally, current evidence support offering reminiscence therapy, physical exercise, music therapy, cognitive rehabilitation, occupational therapy, art therapy and dance therapy interventions for improving the QoL of PwD. However, unlike the group CST, there are wide variations within these therapy programmes. Further research is needed for standardising these seven non-pharmacological interventions, refining therapy manuals and investigating their efficacy in different settings. This systematic review has identified several specific programmes, such as a community-based occupational therapy intervention,^46^ continuous aerobic training,^47^ global postural re-education^50^ and an individual reminiscence therapy programme,^41^ that have level 2 evidence and warrant further research. Moreover, the influence of technology on clinical management of PwD is increasing. The current evidence do not support routine use of any technology for improving the QoL of PwD. However, there is adequate justification for pursuing further research focusing on the efficacy of companion robots,^14^ hearing aids and smart wearables, as well as household devices.

The common challenges pertaining to any non-pharmacological intervention or complex intervention clinical trial should be considered when interpreting available evidence and planning future research in this field. A well-standardised, clearly defined therapy manual will facilitate training therapists, reproducibility of research and future meta-analysis of results of different trials. However, this may increase training costs, and may limit the feasibility of the therapy in a different setting and in people with different types or levels of severity of dementia. Non-pharmacological intervention manuals should have well-defined therapy frameworks, describe specific methods and be adaptable by allowing trained therapists to choose activities from predefined lists that are appropriate to the needs of their patient population. Moreover, it is challenging to identify the key working component of a successful complex intervention, and to disentangle the true effect of a non-pharmacological intervention from the non-specific therapeutic effects during its delivery. Future clinical trials should avoid waiting list or no-intervention controls, and should consider comparing two or more active interventions. The therapy programmes that have level 2 evidence warrant further investigation for identifying their key working components and enhancing their efficacy. Furthermore, a large proportion of PwD reside in residential or nursing homes.^138^ It is important to develop interventions that are appropriate for use in these settings. This systematic review identified 18 studies that provided level 2 evidence for the use of various non-pharmacological interventions for improving the QoL of PwD living in nursing or residential homes.^48,139^ Training care home staff and promoting group participation may aid the implementation and cost-effectiveness of these interventions.

Our findings highlight the need for the following future research directives. First, working toward reaching better consensus on the domains of QoL and their measurement will reduce heterogeneity among future studies and will facilitate synthesis of their results. Future clinical trials investigating the use of various interventions in PwD should include QoL as one of their outcomes. Standardising the reminiscence therapy, physical exercise, music therapy, cognitive rehabilitation, occupational therapy, art therapy and dance therapy interventions and refining their manuals should be prioritised over developing novel therapy programmes. More studies investigating the efficacy of combined pharmacological and non-pharmacological interventions for improving QoL of PwD are warranted. Future non-pharmacological or combined intervention trials investigating PwD should include *a priori* power analyses and recruit adequate sample sizes for conducting subgroup analyses that are necessary for identifying key working components and for further refining of their intervention manuals. Future clinical trials investigating QoL of PwD should consider including appropriate health economic analyses. More qualitative research is needed for improving our understanding of the real-world effectiveness and acceptability of the identified evidence-based interventions for improving QoL of PwD. The evidence-based interventions should be adapted for the needs of people with specific types of dementia, especially non-Alzheimer’s dementia like DLB. And finally, they should be adapted for implementation in residential and nursing home settings, and their efficacy should be investigated in those settings.

In conclusion, individuals’ perspectives and their contexts are influential in determining their QoL.^6^ This comprehensive systematic review shows that the quest for personalised interventions for improving QoL of PwD is not incompatible with the principles of evidence-based medicine.^140^ There is a plethora of interventions with at least level 2 evidence available for clinicians to offer for improving the QoL of PwD. The nature of these interventions emphasise the importance of multidisciplinary care and of including PwD and their caregivers in therapeutic decision-making and further research. The findings of this systematic review may facilitate such a collaborative multidisciplinary approach, and may help clinicians, PwD, caregivers and policy makers to make evidence-informed decisions for improving QoL. Developing evidence-based clinical guidelines is a long process, and it is beyond the scope of any systematic review. However, we hope that the findings of this systematic review will set the stage for achieving this important goal.

## Supporting information

Luxton et al. supplementary material 1 Luxton et al. supplementary material

Luxton et al. supplementary material 2 Luxton et al. supplementary material

Luxton et al. supplementary material 3 Luxton et al. supplementary material

Luxton et al. supplementary material 4 Luxton et al. supplementary material

Luxton et al. supplementary material 5 Luxton et al. supplementary material

Luxton et al. supplementary material 6 Luxton et al. supplementary material

Luxton et al. supplementary material 7 Luxton et al. supplementary material

Luxton et al. supplementary material 8 Luxton et al. supplementary material

Luxton et al. supplementary material 9 Luxton et al. supplementary material

Luxton et al. supplementary material 10 Luxton et al. supplementary material

Luxton et al. supplementary material 11 Luxton et al. supplementary material

## Data Availability

The data that support the findings of this systematic review are available from the corresponding author, A.P.R., upon reasonable request.

## Supplementary material

The supplementary material is available online at https://doi.org/10.1192/bjp.2025.11
