# Supplementary material for: Systematic review of the efficacy of pharmacological and non-pharmacological interventions for improving quality of life of people with dementia
Source: Br J Psychiatry. 2025 Apr 1;228(1):55–67. doi: 10.1192/bjp.2025.11 (PMC12722012; doi:10.1192/bjp.2025.11)
Supplement: Luxton et al. supplementary material 2 — Luxton et al. supplementary material [file S000712502500011Xsup002.docx]

**Supplementary material-9:** Pharmacological or non-pharmacological interventions that improve one or more quality of life domains but not overall quality of life of people with dementia^1,2^.

| **Intervention** | **Highest level of evidence** | **GRADE certainty rating** | **QoL domain affected** |
| --- | --- | --- | --- |
| Traditional opera^3^ | 2 | High | Emotions, compatibility with friends, and entertainment capability |
| ‘Client centred’ global stimulation^4^ | 2 | Moderate | Münchner Lebensqualitäts Dimensionen Liste^5^, Family Survey: Circumstances (of living), Family survey: Mood |
| Art therapy^6^ | 2 | Moderate | Mental component summary of SF-8^7^ |
| Melissa Aromatherapy^8^ | 2 | Moderate | Reduction in social withdrawal and increase in constructive activities. |
| Memantine^9^ | 2 | Moderate | Carer-rated ‘life as a whole’ |
| Music therapy^10^ | 2 | Moderate | Restless behaviour |
| Innovative dementia-orientated Assessment system, case conference^11^ | 2 | Low | Care relationship, positive affect, social isolation and positive self-image |
| Neo (Narrative Approach) case conference^11^ | 2 | Low | Positive affect |
| Meeting Centres Support Program^12^ | 3 | Moderate | Self-esteem, positive affect and feelings of belonging |
| Movement-oriented restorative care^13^ | 3 | Moderate | Positive self-image |
| Aerobic exercise^14^ | 3 | Low | Social relations and environment domains of WHOQOL^15^ |
| A theatre-based communication method^16^ | 3 | Low | Positive affect and social relations |
| Small-scale long-term care setting^17^ | 3 | Low | Social relations, positive affect, having something to do, negative affect |
| Special Care Facility^18^ | 3 | Low | Interest in the environment, Activities of Daily Living function, negative affect |
| Theatre interventions^19^ | 3 | Low | care relation, positive affect, social isolation, feeling at home and having something to do. |
| Multiple training modalities^20^ | 4 | Moderate | Psychiatry domain of WHOQOL-BREF^15^ |
| Daily garden use^21^ | 4 | Low | Negative affect |
| Treadmill aerobic exercise intervention^22^ | 4 | Low | physical functioning, role functioning: physical, general health, Vitality and Social functioning |
| Acupressure^23^ | 4 | Very low | Mood domain of GHQ28^24^ |
| Adaptive riding^25^ | 4 | Very low | Durations of complex active participation |
| Group home-care^26^ | 4 | Very low | Positive affect, restlessness and attachment to others |

GRADE of certainty ratings^1^: High = the true effect is similar to the estimated effect, Moderate = the true effect is probably close to the estimated effect; Low = the true effect may be markedly different from the estimated effect; Very Low = the true effect is probably markedly different from the estimated effect.

**References:**

1. Guyatt GH, Oxman AD, Vist GE, Kunz R, Falck-Ytter Y, Alonso-Coello P, *et al.* GRADE: an emerging consensus on rating quality of evidence and strength of recommendations. *BMJ* 2008; **336**: 924–6.
2. OCEBM Levels of Evidence Working Group. The Oxford 2011 Levels of Evidence. Oxford Centre for Evidence-Based Medicine.
3. Chen X, Li DM, Xu H, Hu ZY. Effect of traditional opera on older adults with dementia. *Geriatr Nurs (Minneap)* 2020; **41**: 118–23.
4. Schecker M, Pirnay-Dummer P, Schmidtke K, Hentrich-Hesse T, Borchardt D. Cognitive interventions in mild Alzheimer’s disease: a therapy-evaluation study on the interaction of medication and cognitive treatment. *Dement Geriatr Cogn Dis Extra* 2013; **3**: 301–11.
5. von Steinbüchel N, Bullinger M, Kirchberger I. Die Münchner Lebensqualitäts-Dimensionen Liste (MLDL): Entwicklung und Prüfung eines Verfahrens zur krankheitsübergreifenden Erfassung von Lebensqualität. , 1999.
6. Hattori H, Hattori C, Hokao C, Mizushima K, Mase T. Controlled study on the cognitive and psychological effect of coloring and drawing in mild Alzheimer’s disease patients. *Geriatr Gerontol Int* 2011; **11**: 431–7.
7. Fukuhara S, Suzukamo Y. Manual of the SF-8 Japanese Version. *Kyoto: Institute for Health Outcomes & Process Evaluation Research* 2004.
8. Ballard CG, O’Brien JT, Reichelt K, Perry EK. Aromatherapy as a safe and effective treatment for the management of agitation in severe dementia: the results of a double-blind, placebo-controlled trial with Melissa. *Journal of Clinical Psychiatry* 2002; **63**: 553–8.
9. Larsson V, Engedal K, Aarsland D, Wattmo C, Minthon L, Londos E. Quality of Life and the Effect of Memantine in Dementia with Lewy Bodies and Parkinson’s Disease Dementia. *Dement Geriatr Cogn Disord* 2012; **32**: 227–34.
10. Prick AJC, Zuidema SU, van Domburg P, Verboon P, Vink AC, Schols J, et al. Effects of a music therapy and music listening intervention for nursing home residents with dementia: a randomized controlled trial. *Front Med (Lausanne)* 2024; **11**: 1304349.
11. Halek M, Reuther S, Muller-Widmer R, Trutschel D, Holle D. Dealing with the behaviour of residents with dementia that challenges: A stepped-wedge cluster randomized trial of two types of dementia-specific case conferences in nursing homes (FallDem). *Int J Nurs Stud* 2020; **104**: 103435.
12. Brooker D, Evans S, Evans S, Bray J, Saibene FL, Scorolli C, *et al.* Evaluation of the implementation of the Meeting Centres Support Program in Italy, Poland, and the UK; exploration of the effects on people with dementia. *Int J Geriatr Psychiatry* 2018; **33**: 883–92.
13. Henskens M, Nauta IM, Scherder EJA, Oosterveld FGJ, Vrijkotte S. Implementation and effects of Movement-oriented Restorative Care in a nursing home - a quasi-experimental study. *BMC Geriatr* 2017; **17**: 243.
14. Fitriana LA, Darmawati I, Nasution LA, Putri ST, Rohaedi S, Anggadiredja K, et al. Effect of Centella Asiatica and Aerobic Exercise in Older Women With Dementia: A Randomized Controlled Trial. *Malaysian Journal of Medicine and Health Sciences* 2021; **17**: 153-60.
15. Lucas-Carrasco R, Skevington SM, Gómez-Benito J, Rejas J, March J. Using the WHOQOL-BREF in persons with dementia: a validation study. *Alzheimer Dis Assoc Disord* 2011; **25**: 345–51.
16. Boersma P, Weert JCM van, Lissenberg-Witte BI, Meijel B van, Dröes R-M. Testing the Implementation of the Veder Contact Method: A Theatre-Based Communication Method in Dementia Care. *Gerontologist* 2019; **59**: 780–91.
17. de Rooij AHPM, Luijkx KG, Schaafsma J, Declercq AG, Emmerink PMJ, Schols JMGA. Quality of life of residents with dementia in traditional versus small-scale long-term care settings: A quasi-experimental study. *Int J Nurs Stud* 2012; **49**: 931–40.
18. Reimer MA, Slaughter S, Donaldson C, Currie G, Eliasziw M. Special care facility compared with traditional environments for dementia care: a longitudinal study of quality of life. *J Am Geriatr Soc* 2004; **52**: 1085–92.
19. van Dijk AM, van Weert JCM, Droes RM. Does theatre improve the quality of life of people with dementia? *Int Psychogeriatr* 2012; **24**: 367–81.
20. Tai SY, Hsu CL, Huang SW, Ma TC, Hsieh WC, Yang YH. Effects of multiple training modalities in patients with Alzheimer’s disease: a pilot study. *Neuropsychiatr Dis Treat* 2016; **12**: 2843–9.
21. van der Velde-van Buuringen M, Achterberg WP, Caljouw MAA. Daily garden use and quality of life in persons with advanced dementia living in a nursing home: A feasibility study. *Nurs Open* 2020; **21**: 21.
22. Abd El-Kader SM, Al-Jiffri OH. Aerobic exercise improves quality of life, psychological well-being and systemic inflammation in subjects with Alzheimer’s disease. *Afr Health Sci* 2016; **16**: 1045–55.
23. Simoncini M, Gatti A, Quirico P, Balla S, Capellero B, Obialero R, *et al.* Acupressure in insomnia and other sleep disorders in elderly institutionalized patients suffering from Alzheimer’s disease. *Aging Clin Exp Res* 2015; **27**: 37–42.
24. Goldberg DP, Gater R, Sartorius N, Ustun TB, Piccinelli M, Gureje O, *et al.* The validity of two versions of the GHQ in the WHO study of mental illness in general health care. *Psychol Med* 1997; **27**: 191–7.
25. Lassell R, Wood W, Schmid AA, Cross JE. A comparison of quality of life indicators during two complementary interventions: adaptive gardening and adaptive riding for people with dementia. *Complement Ther Med* 2021; **57**: 102658.
26. Yokota O, Fujisawa Y, Takahashi J, Terada S, Ishihara T, Nakashima H, *et al.* Effects of group-home care on behavioral symptoms, quality of life, and psychotropic drug use in patients with frontotemporal dementia. *J Am Med Dir Assoc* 2006; **7**: 335–7.
