# Supplementary material for: Systematic review of the efficacy of pharmacological and non-pharmacological interventions for improving quality of life of people with dementia
Source: Br J Psychiatry. 2025 Apr 1;228(1):55–67. doi: 10.1192/bjp.2025.11 (PMC12722012; doi:10.1192/bjp.2025.11)
Supplement: Luxton et al. supplementary material 3 — Luxton et al. supplementary material [file S000712502500011Xsup003.docx]

| **Supplementary material-2: A report detailing the search strategies for this systematic review** |
| --- |

**Database Searching**

| Name of Database | Platform | Date searched | # of results |
| --- | --- | --- | --- |
| Medline | Ovid MEDLINE(R) ALL <1946 to April 09, 2021> | 12-04-2021 | 6,070 |
| Embase | Ovid Embase <1974 to 2021 April 09> | 12-04-2021 | 1,439 |
| PsycINFO | Ovid PsycINFO <1806 to April Week 1 2021> | 12-04-2021 | 3,276 |
| CINAHL Plus with Full Text | EBSCOhost Research Databases  - CINAHL Plus with Full Text | 12-04-2021 | 2,969 |
| Scopus | Elsevier | 12-04-2021 | 7,243 |
| Web of Science Core Collection | ISI Web of Knowledge |  | 5,222 |
| Cochrane Library | www.cochranelibrary.com |  | 2,681 |
|  | | | |
| Total number of results |  |  | 28,900 |
| Duplicates found |  |  | 14,157 |
| Results after duplicates and other reference types removed |  |  | 13,055 |

**Search History for Databases for published literature**

**Platform**: Ovid

**Database**: Ovid MEDLINE(R) ALL <1946 to April 09, 2021>

**Date searched**: 12-04-2021

--------------------------------------------------------------------------------

1 (dementia or alzheimer*).tw,kw,kf. (230877)

2 exp Dementia/ (172748)

3 1 or 2 (270866)

4 (intervention* or therap* or treatment*).tw,kw,kf. (7070697)

5 exp Therapeutics/ or exp Drug Therapy/ or exp Psychotherapy/ (4826667)

6 4 or 5 (9794545)

7 3 and 6 (105649)

8 ("quality of life" or "quality-of-life" or "life quality" or wellbeing or "well-being" or "well being" or "life satisfaction").tw,kw,kf. (396558)

9 exp "Quality of Life"/ (206854)

10 8 or 9 (450726)

11 7 and 10 (6713)

12 Animals/ not Humans/ (4777601)

13 11 not 12 (6670)

14 limit 13 to english language (6070)

***************************

**Platform**: Ovid

**Database**: Embase <1974 to 2021 April 09>

**Date searched**: 12-04-2021

--------------------------------------------------------------------------------

1 (dementia or alzheimer*).tw,kw. (327994)

2 exp Dementia/ (379375)

3 1 or 2 (441364)

4 (intervention* or therap* or treatment*).tw,kw. (9542635)

5 exp Therapy/ or exp Drug Therapy/ or exp Psychotherapy/ (9092197)

6 4 or 5 (13901932)

7 3 and 6 (193553)

8 ("quality of life" or "quality-of-life" or "life quality" or wellbeing or "well-being" or "well being" or "life satisfaction").tw,kw. (606520)

9 exp "Quality of Life"/ (529865)

10 8 or 9 (750254)

11 7 and 10 (12986)

12 exp Animals/ or exp Invertebrate/ or Animal Experiment/ or Animal Model/ or Animal Tissue/ or Animal Cell/ or Nonhuman/ (29023697)

13 Human/ or Normal Human/ or Human Cell/ (22390603)

14 12 and 13 (22322412)

15 12 not 14 (6701285)

16 11 not 15 (12830)

17 limit 16 to (english language and exclude medline journals) (1439)

***************************

**Platform**: Ovid

**Database**: APA PsycINFO <1806 to April Week 1 2021>

**Date searched**: 12-04-2021

--------------------------------------------------------------------------------

1 (dementia or alzheimer*).tw,hw. (106262)

2 exp Dementia/ or exp Alzheimer's Disease/ (80480)

3 1 or 2 (106837)

4 (intervention* or therap* or treatment*).tw,hw. (1235034)

5 exp Treatment/ or exp Drug Therapy/ or exp Psychotherapy/ (1088627)

6 4 or 5 (1567147)

7 3 and 6 (45016)

8 ("quality of life" or "quality-of-life" or "life quality" or wellbeing or "well-being" or "well being" or "life satisfaction").tw,hw. (179705)

9 exp "Quality of Life"/ (45239)

10 8 or 9 (180831)

11 7 and 10 (3701)

12 limit 11 to (human and english language) (3276)

***************************

**Interface**: EBSCOhost Research Databases 
**Search Screen**: Advanced Search 
**Database**: CINAHL Plus with Full Text

**Date searched**: 12-04-2021

--------------------------------------------------------------------------------

| **#** | **Query** | **Results** |
| --- | --- | --- |
| S1 | TI ( dementia OR alzheimer* ) OR AB ( dementia OR alzheimer* ) OR SU ( dementia OR alzheimer* ) | 95,501 |
| S2 | (MH "Dementia+") | 76,072 |
| S3 | S1 OR S2 | 97,120 |
| S4 | TI ( intervention* or therap* or treatment* ) OR AB ( intervention* or therap* or treatment* ) OR SU ( intervention* or therap* or treatment* ) | 2,475,669 |
| S5 | (MH "Therapeutics+") OR (MH "Drug Therapy+") OR (MH "Psychotherapy+") | 1,716,354 |
| S6 | S4 OR S5 | 3,223,398 |
| S7 | S3 AND S6 | 49,783 |
| S8 | TI ( "quality of life" or "quality-of-life" or "life quality" or wellbeing or "well-being" or "well being" or "life satisfaction" ) OR AB ( "quality of life" or "quality-of-life" or "life quality" or wellbeing or "well-being" or "well being" or "life satisfaction" ) OR SU ( "quality of life" or "quality-of-life" or "life quality" or wellbeing or "well-being" or "well being" or "life satisfaction" ) | 260,280 |
| S9 | (MH "Quality of Life+") | 126,338 |
| S10 | S8 OR S9 | 267,526 |
| S11 | S7 AND S10 | 5,401 |
| S12 | (MH "Animals+") NOT (MH "Human") | 87,712 |
| S13 | S11 NOT S12  Limiters - English Language | 2,969 |

***************************

**Interface**: Elsevier

**Database**: Scopus

**Date searched**: 12-04-2021

--------------------------------------------------------------------------------

| **#** | **Query** | **Results** |
| --- | --- | --- |
| #1 | TITLE-ABS-KEY ( dementia  OR  alzheimer* ) | 353,445 |
| #2 | TITLE-ABS-KEY ( intervention*  OR  therap*  OR  treatment* ) | 12,273,707 |
| #3 | TITLE-ABS-KEY ( "quality of life"  OR  "quality-of-life"  OR  "life quality"  OR  wellbeing  OR  "well-being"  OR  "well being"  OR  "life satisfaction" ) | 758,951 |
| #4 | #1 AND #2 AND #3 | 9,775 |
| #5 | ( TITLE-ABS-KEY ( dementia OR alzheimer* ) ) AND ( TITLE-ABS-KEY ( intervention* OR therap* OR treatment* ) ) AND ( TITLE-ABS-KEY ( "quality of life" OR "quality-of-life" OR "life quality" OR wellbeing OR "well-being" OR "well being" OR "life satisfaction" ) ) AND ( LIMIT-TO ( LANGUAGE , "English" ) ) AND ( LIMIT-TO ( SRCTYPE , "j" ) ) AND ( LIMIT-TO ( DOCTYPE , "ar" ) OR LIMIT-TO ( DOCTYPE , "re" ) ) AND ( LIMIT-TO ( SUBJAREA , "MEDI" ) OR LIMIT-TO ( SUBJAREA , "NURS" ) OR LIMIT-TO ( SUBJAREA , "NEUR" ) OR LIMIT-TO ( SUBJAREA , "PSYC" ) OR LIMIT-TO ( SUBJAREA , "SOCI" ) OR LIMIT-TO ( SUBJAREA , "PHAR" ) OR LIMIT-TO ( SUBJAREA , "HEAL" ) OR LIMIT-TO ( SUBJAREA , "MULT" ) ) | 7,243 |

***************************

**Interface**: ISI Web of Knowledge

**Database**: Web of Science Core Collection

**Indexes**: SCI-EXPANDED, SSCI, A&HCI, CPCI-S, CPCI-SSH, BKCI-S, BKCI-SSH, ESCI, CCR-EXPANDED, IC

**Timespan:** All years

**Date searched**: 12-04-2021

--------------------------------------------------------------------------------

| **#** | **Query** | **Results** |
| --- | --- | --- |
| #1 | **TOPIC:** (dementia or alzheimer*) | 344,617 |
| #2 | **TOPIC:** (intervention* or therap* or treatment*) | 7,920,550 |
| #3 | **TOPIC:** ("quality of life" or "quality-of-life" or "life quality" or wellbeing or "well-being" or "well being" or "life satisfaction") | 576,798 |
| #4 | #3 AND #2 AND #1 | 7,010 |
| #5 | #3 AND #2 AND #1  **Refined by:** **LANGUAGES:** ( ENGLISH ) AND **DOCUMENT TYPES:** ( ARTICLE OR REVIEW ) AND **RESEARCH AREAS:** ( GERIATRICS GERONTOLOGY OR NEUROSCIENCES NEUROLOGY OR BIOMEDICAL SOCIAL SCIENCES OR PSYCHIATRY OR PSYCHOLOGY OR NURSING OR HEALTH CARE SCIENCES SERVICES OR PHARMACOLOGY PHARMACY OR REHABILITATION OR PUBLIC ENVIRONMENTAL OCCUPATIONAL HEALTH OR RESEARCH EXPERIMENTAL MEDICINE OR SOCIAL WORK OR BEHAVIORAL SCIENCES OR SOCIAL ISSUES OR SOCIAL SCIENCES OTHER TOPICS ) AND **WEB OF SCIENCE CATEGORIES:** ( GERIATRICS GERONTOLOGY OR GERONTOLOGY OR PSYCHIATRY OR CLINICAL NEUROLOGY OR NEUROSCIENCES OR NURSING OR HEALTH CARE SCIENCES SERVICES OR PHARMACOLOGY PHARMACY OR REHABILITATION OR PSYCHOLOGY CLINICAL OR PSYCHOLOGY OR PSYCHOLOGY SOCIAL OR PUBLIC ENVIRONMENTAL OCCUPATIONAL HEALTH OR MEDICINE RESEARCH EXPERIMENTAL OR SOCIAL ISSUES OR HEALTH POLICY SERVICES OR PSYCHOLOGY MULTIDISCIPLINARY OR MEDICINE GENERAL INTERNAL OR SOCIAL WORK OR BEHAVIORAL SCIENCES OR PSYCHOLOGY DEVELOPMENTAL OR PSYCHOLOGY APPLIED OR PSYCHOLOGY BIOLOGICAL OR SOCIOLOGY OR SOCIAL SCIENCES BIOMEDICAL ) | 5,222 |

***************************

**Interface**: Cochrane Library

**Database**: Cochrane Database of Systematic Reviews

**Date searched**: 12-04-2021

--------------------------------------------------------------------------------

ID Search Hits

#1 (dementia OR alzheimer*):ti,ab,kw (Word variations have been searched) 20035

#2 MeSH descriptor: [Dementia] explode all trees 6074

#3 #1 OR #2 20293

#4 (intervention* OR therap* OR treatment*):ti,ab,kw (Word variations have been searched) 1221917

#5 MeSH descriptor: [Therapeutics] explode all trees 309166

#6 MeSH descriptor: [Drug Therapy] explode all trees 142349

#7 MeSH descriptor: [Psychotherapy] explode all trees 24514

#8 #4 OR #5 OR #6 OR #7 1263799

#9 ("quality of life" or "quality-of-life" or "life quality" or wellbeing or "well-being" or "well being" or "life satisfaction"):ti,ab,kw (Word variations have been searched) 127600

#10 MeSH descriptor: [Quality of Life] explode all trees 24829

#11 #9 OR #10 127600

#12 #3 AND #8 AND #11 in Cochrane Reviews, Trials 2681

***************************
