# Supplementary material for: Systematic review of the efficacy of pharmacological and non-pharmacological interventions for improving quality of life of people with dementia
Source: Br J Psychiatry. 2025 Apr 1;228(1):55–67. doi: 10.1192/bjp.2025.11 (PMC12722012; doi:10.1192/bjp.2025.11)
Supplement: Luxton et al. supplementary material 4 — Luxton et al. supplementary material [file S000712502500011Xsup004.docx]

**Supplementary material-3:** List of all studies included in the first round of the systematic review

1 Abd El- Kader SM. Role of Aerobic Exercise Training in Changing Exercise Tolerance and Quality of Life in Alzheimer’s Disease. *Eur J Gen Med* 2011; **8**: 1–6.

2 Abd El-Kader SM, Al-Jiffri OH. Aerobic exercise improves quality of life, psychological well-being and systemic inflammation in subjects with Alzheimer’s disease. *Afr Health Sci* 2016; **16**: 1045–55.

3 Adrait A, Perrot X, Nguyen MF, Gueugnon M, Petitot C, Collet L, *et al.* Do Hearing Aids Influence Behavioral and Psychological Symptoms of Dementia and Quality of Life in Hearing Impaired Alzheimer’s Disease Patients and Their Caregivers? *J Alzheimers Dis* 2017; **58**: 109–21.

4 Aguiar P, Monteiro L, Feres A, Gomes I, Melo A. Rivastigmine transdermal patch and physical exercises for Alzheimer’s disease: a randomized clinical trial. *Curr Alzheimer Res* 2014; **11**: 532–7.

5 Aguirre E, Hoare Z, Streater A, Spector A, Woods B, Hoe J, *et al.* Cognitive stimulation therapy (CST) for people with dementia--who benefits most? *Int J Geriatr Psychiatry* 2013; **28**: 284–90.

6 Aisen PS, Schafer KA, Grundman M, Pfeiffer E, Sano M, Davis KL, *et al.* Effects of rofecoxib or naproxen vs placebo on Alzheimer disease progression: a randomized controlled trial. *JAMA* 2003; **289**: 2819–26.

7 Alvares-Pereira G, Silva-Nunes M v, Spector A. Validation of the cognitive stimulation therapy (CST) program for people with dementia in Portugal. *Aging Ment Health* 2021; **25**:1019-28.

8 Amieva H, Robert PH, Grandoulier AS, Meillon C, de Rotrou J, Andrieu S, *et al.* Group and individual cognitive therapies in Alzheimer’s disease: the ETNA3 randomized trial. *Int Psychogeriatr* 2016; **28**: 707–17.

9 Astell AJ, Smith SK, Potter S, Preston-Jones E. Computer Interactive Reminiscence and Conversation Aid groups—Delivering cognitive stimulation with technology. *Alzheimers Dement (N Y)* 2018; **4**: 481–7.

10 Avila R, Bottino CMC, Carvalho IAM, Santos CB, Seral C, Miotto EC. Neuropsychological rehabilitation of memory deficits and activities of daily living in patients with Alzheimer’s disease: a pilot study. *Braz J Med Biol Res* 2004; **37**: 1721–9.

11 Ballard CG, O’Brien JT, Reichelt K, Perry EK. Aromatherapy as a safe and effective treatment for the management of agitation in severe dementia: the results of a double-blind, placebo-controlled trial with Melissa. *J Clin Psychiatry* 2002; **63**: 553–8.

12 Bergh S, Selbaek G, Engedal K. Discontinuation of antidepressants in people with dementia and neuropsychiatric symptoms (DESEP study): double blind, randomised, parallel group, placebo controlled trial. *BMJ* 2012; **344**: e1566.

13 Berk L, Warmenhoven F, Stiekema APM, van Oorsouw K, van Os J, de Vugt M, *et al.* Mindfulness-Based Intervention for People With Dementia and Their Partners: Results of a Mixed-Methods Study. *Front Aging Neurosci* 2019; **11**: 92.

14 Binns E, Kerse N, Peri K, Cheung G, Taylor D. Combining cognitive stimulation therapy and fall prevention exercise (CogEx) in older adults with mild to moderate dementia: a feasibility randomised controlled trial. *Pilot Feasibility Stud* 2020; **6**: 108.

15 Birkenhager-Gillesse EG, Achterberg WP, Janus SIM, Kollen BJ, Zuidema SU. Effects of caregiver dementia training in caregiver-patient dyads: A randomized controlled study. *Int J Geriatr Psychiatry* 2020; **35**: 1376–84.

16 Boersma P, Weert JCM van, Lissenberg-Witte BI, Meijel B van, Dröes R-M. Testing the Implementation of the Veder Contact Method: A Theatre-Based Communication Method in Dementia Care. *Gerontologist* 2019; **59**: 780–91.

17 Borges-Machado F, Ribeiro O, Sampaio A, Marques-Aleixo I, Meireles J, Carvalho J. Feasibility and Impact of a Multicomponent Exercise Intervention in Patients With Alzheimer’s Disease: A Pilot Study. *Am J Alzheimers Dis Other Demen* 2019; **34**: 95–103.

18 Bottini G, Vallar G, Cappa S, Monza GC, Scarpini E, Baron P, *et al.* Oxiracetam in dementia: a double-blind, placebo-controlled study. *Acta Neurol Scand* 1992; **86**: 237–41.

19 Brodaty H, Low LF. Making Memories: pilot evaluation of a new program for people with dementia and their caregivers. *Australas J Ageing* 2004; **23**: 144–6.

20 Bromundt V, Wirz-Justice A, Boutellier M, Winter S, Haberstroh M, Terman M, *et al.* Effects of a dawn-dusk simulation on circadian rest-activity cycles, sleep, mood and well-being in dementia patients. *Exp Gerontol* 2019; **124**: 110641.

21 Brooker D, Evans S, Evans S, Bray J, Saibene FL, Scorolli C, *et al.* Evaluation of the implementation of the Meeting Centres Support Program in Italy, Poland, and the UK; exploration of the effects on people with dementia. *Int J Geriatr Psychiatry* 2018; **33**: 883–92.

22 Burns A, Rossor M, Hecker J, Gauthier S, Petit H, Moller H, *et al.* The effects of donepezil in Alzheimer’s disease-results from a multinational trial. *Dement Geriatr Cogn Disord* 1999; **10**: 237–44.

23 Camic PM, Tischler V, Pearman CH. Viewing and making art together: a multi-session art-gallery-based intervention for people with dementia and their carers. *Aging Ment Health* 2014; **18**: 161–8.

24 Cao YY, Qian L, Yu WG, Li TT, Mao S, Han GW. Donepezil plus memantine versus donepezil alone for treatment of concomitant Alzheimer’s disease and chronic obstructive pulmonary disease: a retrospective observational study. *J Int Med Res*2020; **48**: 12.

25 Capotosto E, Belacchi C, Gardini S, Faggian S, Piras F, Mantoan V, *et al.* Cognitive stimulation therapy in the Italian context: its efficacy in cognitive and non-cognitive measures in older adults with dementia. *Int J Geriatr Psychiatry* 2017; **32**: 331–40.

26 Caramelli P, Laks J, Palmini ALF, Nitrini R, Chaves MLF, Forlenza O v, *et al.* Effects of galantamine and galantamine combined with nimodipine on cognitive speed and quality of life in mixed dementia: a 24-week, randomized, placebo-controlled exploratory trial (the REMIX study). *Arq Neuropsiquiatr* 2014; **72**: 411–7.

27 Carbone E, Gardini S, Pastore M, Piras F, Vincenzi M, Borella E. Cognitive Stimulation Therapy (CST) for older adults with mild-to-moderate dementia in Italy: effects on cognitive functioning and on emotional and neuropsychiatric symptoms. *J Gerontol B Psychol Sci Soc Sci* 2021; **76**: 1700-10.

28 Chapman SB, Weiner ME, Rackley A, Hynan LS, Zientz J. Effects of cognitive-communication-stimulation for Alzheimer’s disease patients treated with donepezil. *J Speech Lang Hear Res* 2004; **47**: 1149–63.

29 Charlesworth G, Burnell K, Crellin N, Hoare Z, Hoe J, Knapp M, *et al.* Peer support and reminiscence therapy for people with dementia and their family carers: a factorial pragmatic randomised trial. *J Neurol Neurosurg Psychiatry* 2016; **87**: 1218–28.

30 Charras K, Gzil F. Judging a Book by Its Cover: Uniforms and Quality of Life in Special Care Units for People With Dementia. *Am J Alzheimers Dis Other Demen* 2013; **28**: 450–8.

31 Charras K, Mabire JB, Bouaziz N, Deschamps P, Froget B, de Malherbe A, *et al.* Dance intervention for people with dementia: Lessons learned from a small-sample crossover explorative study. *Arts in Psychotherapy* 2020; **70**.

32 Chaudhry N, Tofique S, Husain N, Couture D, Glasgow P, Husain M, *et al.* Montessori intervention for individuals with dementia: feasibility study of a culturally adapted psychosocial intervention in Pakistan (MIRACLE). *BJPsych Open* 2020; **6**: e69.

33 Chen K, Lou VWQ, Tan KCK, Wai MY, Chan LL. Effects of a Humanoid Companion Robot on Dementia Symptoms and Caregiver Distress for Residents in Long-Term Care. *J Am Med Dir Assoc* 2020; **21**: 1724- 28.e3.

34 Chen X, Li DM, Xu H, Hu ZY. Effect of traditional opera on older adults with dementia. *Geriatr Nurs* 2020; **41**: 118–23.

35 Chenoweth L, Forbes I, Fleming R, King MT, Stein-Parbury J, Luscombe G, *et al.* PerCEN: a cluster randomized controlled trial of person-centered residential care and environment for people with dementia. *Int Psychogeriatr* 2014; **26**: 1147–60.

36 Chenoweth L, Jeon YH. Determining the efficacy of Dementia Care Mapping as an outcome measure and a process for change: a pilot study. *Aging Ment Health* 2007; **11**: 237–45.

37 Chenoweth L, King MT, Jeon YH, Brodaty H, Stein-Parbury J, Norman R, *et al.* Caring for Aged Dementia Care Resident Study (CADRES) of person-centred care, dementia-care mapping, and usual care in dementia: a cluster-randomised trial. *Lancet Neurology* 2009; **8**: 317–25.

38 Cheon Y, Park J, Joe KH, Kim DJ. The effect of 12-week open-label memantine treatment on cognitive function improvement in patients with alcohol-related dementia. *Int J Neuropsychopharmacol* 2008; **11**: 971–83.

39 Cheston R, Howells L. A feasibility study of translating “Living Well with Dementia” groups into a Primary Care Improving Access to Psychological Therapy service (innovative practice). *Dementia* 2016; **15**: 273–8.

40 Chew J, Chong MS, Fong YL, Tay L. Outcomes of a multimodal cognitive and physical rehabilitation program for persons with mild dementia and their caregivers: a goal-oriented approach. *Clin Interv Aging* 2015; **10**: 1687–94.

41 Cho HK. The Effects of Music Therapy-Singing Group on Quality of Life and Affect of Persons With Dementia: A Randomized Controlled Trial. *Front Med* 2018; **5**: 279.

42 Chu CH, Puts M, Brooks D, Parry M, McGilton KS. A Feasibility Study of a Multifaceted Walking Intervention to Maintain the Functional Mobility, Activities of Daily Living, and Quality of Life of Nursing Home Residents With Dementia. *Rehabil Nurs* 2020; **45**: 204–17.

43 Chung JCC. An intergenerational reminiscence programme for older adults with early dementia and youth volunteers: values and challenges. *Scand J Caring Sci* 2009; **23**: 259–64.

44 Churcher Clarke A, Chan J, Stott J, Royan L, Spector A. An adapted mindfulness intervention for people with dementia in care homes: Feasibility pilot study. *Int J Geriatr Psychiatry* 2017; **32**: e123–31.

45 Clare L, Kudlicka A, Oyebode JR, Jones RW, Bayer A, Leroi I, *et al.* Individual goal-oriented cognitive rehabilitation to improve everyday functioning for people with early-stage dementia: a multicentre randomised controlled trial (the GREAT trial). *Int J Geriatr Psychiatry* 2019; **34**: 709‐721.

46 Coelho T, Marques C, Moreira D, Soares M, Portugal P, Marques A, *et al.* Promoting Reminiscences with Virtual Reality Headsets: A Pilot Study with People with Dementia. *Int J Environ Res Public Health* 2020; **17**: 12.

47 Coen RF, Flynn B, Rigney E, O’Connor E, Fitzgerald L, Murray C, *et al.* Efficacy of a cognitive stimulation therapy programme for people with dementia. *Ir J Psychol Med* 2011; **28**: 145–7.

48 Collins H, van Puymbroeck M, Hawkins BL, Vidotto J. The Impact of a Sensory Garden for People with Dementia. *Ther Recreation J* 2020; **54**: 48–63.

49 Collins RN, Gilligan LJ, Poz R. The Evaluation of a Compassion-Focused Therapy Group for Couples Experiencing a Dementia Diagnosis. *Clin Gerontol* 2018; **41**: 474–86.

50 Cornelis E, Gorus E, Beyer I, van Puyvelde K, Lieten S, Versijpt J, *et al.* A retrospective study of a multicomponent rehabilitation programme for community-dwelling persons with dementia and their caregivers. *Br J Occup Ther* 2018; **81**: 5–14.

51 Cove J, Jacobi N, Donovan H, Orrell M, Stott J, Spector A. Effectiveness of weekly cognitive stimulation therapy for people with dementia and the additional impact of enhancing cognitive stimulation therapy with a carer training program. *Clin Interv Aging* 2014; **9**: 2143–50.

52 Craig C, Hiskey S, Royan L, Poz R, Spector A. Compassion focused therapy for people with dementia: A feasibility study. *Int J Geriatr Psychiatry* 2018; **33**: 1727–35.

53 Cunningham S, Brill M, Whalley JH, Read R, Anderson G, Edwards S, *et al.* Assessing Wellbeing in People Living with Dementia Using Reminiscence Music with a Mobile App (Memory Tracks): A Mixed Methods Cohort Study. *J Healthc Eng* 2019; **2019**: 8924273.

54 D’Cunha NM, McKune AJ, Isbel S, Kellett J, Georgousopoulou EN, Naumovski N. Psychophysiological Responses in People Living with Dementia after an Art Gallery Intervention: An Exploratory Study. *J Alzheimers Dis* 2019; **72**: 549–62.

55 D’Onofrio G, Sancarlo D, Raciti M, Burke M, Teare A, Kovacic T, *et al.* MARIO Project: Validation and Evidence of Service Robots for Older People with Dementia. *J Alzheimers Dis* 2019; **68**: 1587–601.

56 Davis RN, Massman PJ, Doody RS. Cognitive intervention in Alzheimer disease: a randomized placebo-controlled study. *Alzheimer Dis Assoc Disord* 2001; **15**: 1–9.

57 de Rooij AHPM, Luijkx KG, Schaafsma J, Declercq AG, Emmerink PMJ, Schols JMGA. Quality of life of residents with dementia in traditional versus small-scale long-term care settings: A quasi-experimental study. *Int J Nurs Stud* 2012; **49**: 931–40.

58 de Vocht HM, Hoogeboom AMGM, van Niekerk B, den Ouden MEM. The Impact of Individualized Interaction on the Quality of Life of Elderly Dependent on Care as a Result of Dementia: A Study with a Pre-Post Design. *Dement Geriatr Cogn Disord* 2015; **39**: 272–80.

59 Doody RS, D’Amico S, Cutler AJ, Davis CS, Shin P, Ledon F, *et al.* An open-label study to assess safety, tolerability, and effectiveness of dextromethorphan/quinidine for pseudobulbar affect in dementia: PRISM II results. *CNS Spectr* 2016; **21**: 450–9.

60 Doody RS, Raman R, Farlow M, Vellas B, Joffe S, Kieburtz K, *et al.* A Phase 3 Trial of Semagacestat for Treatment of Alzheimer’s Disease A BS T R AC T. *N Engl J Med* 2013; **369**: 341–50.

61 Duff S, Nightingale D. Alternative approaches to supporting individuals with dementia: enhancing quality of life through hypnosis. *Alzheimers care today* 2007; **8**: 321–31.

62 Edwards CA, McDonnell C, Merl H. An evaluation of a therapeutic garden’s influence on the quality of life of aged care residents with dementia. *Dementia* 2013; **12**: 494–510.

63 Enette L, Vogel T, Merle S, Valard-Guiguet AG, Ozier-Lafontaine N, Neviere R, *et al.* Effect of 9 weeks continuous vs. interval aerobic training on plasma BDNF levels, aerobic fitness, cognitive capacity and quality of life among seniors with mild to moderate Alzheimer’s disease: a randomized controlled trial. *Eur Rev Aging Phys Act* 2020; **17**: 2.

64 el Alili M, Smaling HJA, Joling KJ, Achterberg WP, Francke AL, Bosmans JE, *et al.* Cost-effectiveness of the Namaste care family program for nursing home residents with advanced dementia in comparison with usual care: a cluster-randomized controlled trial. *BMC Health Serv Res* 2020; **20**: 831.

65 Fan A, Zhou JW. Effect of the combination of donepezil with hyperbaric oxygen therapy and functional rehabilitation training on Parkinson’s disease dementia and the neurological function system. *Int J Clin Exp Med* 2020; **13**: 5867–75.

66 Ferrer B, del Valle A. A Rehabilitation Program for Alzheimer’s Disease. *J Nurs Res* 2014; **22**: 192–9.

67 Fialho PPA, Koenig AM, dos Santos MDL, Barbosa MT, Caramelli P. Positive effects of a cognitive-behavioral intervention program for family caregivers of demented elderly. *Arq Neuropsiquiatr* 2012; **70**: 786–92.

68 Foloppe DA, Richard P, Yamaguchi T, Etcharry-Bouyx F, Allain P. The potential of virtual reality-based training to enhance the functional autonomy of Alzheimer’s disease patients in cooking activities: A single case study. *Neuropsychol Rehabil* 2018; **28**: 709–33.

69 Fontaine CS, Hynan LS, Koch K, Martin-Cook K, Svetlik D, Weiner MF. A double-blind comparison of olanzapine versus risperidone in the acute treatment of dementia-related behavioral disturbances in extended care facilities. *J Clin Psychiatry* 2003; **64**: 726–30.

70 Garrido S, Dunne L, Stevens CJ, Chang E, Clements-Cortes A. Music Playlists for People with Dementia: Trialing A Guide for Caregivers. *J Alzheimers Dis* 2020; **77**: 219–26.

71 Gavrilova SI, Ferri CP, Mikhaylova N, Sokolova O, Banerjee S, Prince M. Helping carers to care-The 10/66 dementia research group’s randomized control trial of a caregiver intervention in Russia. *Int J Geriatr Psychiatry* 2009; **24**:347-54

72 Gault LM, Ritchie CW, Robieson WZ, Pritchett Y, Othman AA, Lenz RA. A phase 2 randomized, controlled trial of the α7 agonist ABT-126 in mild-to-moderate Alzheimer’s dementia. *Alzheimers Dement (N Y)* 2015; **1**: 81–90.

73 Gibbor L, Forde L, Yates L, Orfanos S, Komodromos C, Page H, *et al.* A feasibility randomised control trial of individual cognitive stimulation therapy for dementia: impact on cognition, quality of life and positive psychology. *Aging Ment Health* 2021; **25**: 999-1007.

74 Goyder J, Orrell M, Wenborn J, Spector A, Goyder J, Orrell M, *et al.* Staff training using STAR: a pilot study in UK care homes. *Int Psychogeriatr* 2012; **24**: 911–20.

75 Graff MJL, Vernooij-Dassen MJM, Thijssen M, Dekker J, Hoefnagels WHL, OldeRikkert MGM. Effects of community occupational therapy on quality of life, mood, and health status in dementia patients and their caregivers: A randomized controlled trial. *J Gerontol A Biol Sci Med Sci* 2007; **62**: 1002–9.

76 Green RC, Schneider LS, Amato DA, Beelen AP, Wilcock G, Swabb EA, *et al.* Effect of tarenflurbil on cognitive decline and activities of daily living in patients with mild Alzheimer disease: A randomized controlled trial. *JAMA* 2009; **302**: 2557–64.

77 Graff MJL, Vernooij-Dassen MJM, Zajec J, Olde-Rikkert MGM, Hoefnagels WHL, Dekker J. How can occupational therapy improve the daily performance and communication of an older patient with dementia and his primary caregiver?:A case study. *Dementia* 2006; **5**: 503–32.

78 Gridley K, Brooks J, Birks Y, Baxter K, Parker G. Improving care for people with dementia: development and initial feasibility study for evaluation of life story work in dementia care. *Health Soc Care Deliv Res* 2016; **8**: 8.

79 Gresham M, Heffernan M, Brodaty H, Haapala I, Biggs S, Kurrle S. The Going to Stay at Home program: combining dementia caregiver training and residential respite care. *Int Psychogeriatr* 2018; **30**: 1697–706.

80 Guerra M, Ferri CP, Fonseca M, Banerjee S, Prince M. Helping carers to care: the 10/66 dementia research group’s randomized control trial of a caregiver intervention in Peru. *Braz J Psychiatry*2011; **33**: 47–54.

81 Guerriero F, Botarelli E, Mele G, Polo L, Zoncu D, Renati P, *et al.* An innovative intervention for the treatment of cognitive impairment–emisymmetric bilateral stimulation improves cognitive functions in alzheimer’s disease and mild cognitive impairment: An open-label study. *Neuropsychiatr Dis Treat* 2015; **11**: 2391–404.

82 Gustafsson C, Svanberg C, Mullersdorf M. Using a Robotic Cat in Dementia Care: A Pilot Study. *J Gerontol Nurs* 2015; **41**: 46–56.

83 Halek M, Reuther S, Muller-Widmer R, Trutschel D, Holle D. Dealing with the behaviour of residents with dementia that challenges: A stepped-wedge cluster randomized trial of two types of dementia-specific case conferences in nursing homes (FallDem). *Int J Nurs Stud* 2020; **104**: 103435.

84 Hamill M, Smith L, Röhricht F. ‘Dancing down memory lane’: Circle dancing as a psychotherapeutic intervention in dementia—a pilot study. *Dementia (14713012)* 2012; **11**: 709–24.

85 Hammarlund RA, Whatley KL, Zielinski MH, Jubert JC. Benefits of Affordable Robotic Pet Ownership in Older Adults With Dementia. *J Gerontol Nurs* 2021; **47**: 18–22.

86 Hattori H, Hattori C, Hokao C, Mizushima K, Mase T. Controlled study on the cognitive and psychological effect of coloring and drawing in mild Alzheimer’s disease patients. *Geriatr Gerontol Int* 2011; **11**: 431–7.

87 Henskens M, Nauta IM, Drost KT, Scherder EJA. The effects of movement stimulation on activities of daily living performance and quality of life in nursing home residents with dementia: a randomized controlled trial. *Clin Interv Aging* 2018; **13**: 805–16.

88 Henskens M, Nauta IM, Scherder EJA, Oosterveld FGJ, Vrijkotte S. Implementation and effects of Movement-oriented Restorative Care in a nursing home - a quasi-experimental study. *BMC Geriatr* 2017; **17**: 243.

89 Hindle J v, Watermeyer TJ, Roberts J, Brand A, Hoare Z, Martyr A, *et al.* Goal-orientated cognitive rehabilitation for dementias associated with Parkinson’s disease-A pilot randomised controlled trial. *Int J Geriatr Psychiatry* 2018; **33**: 718–28.

90 Hoffmann K, Sobol NA, Frederiksen KS, Beyer N, Vogel A, Vestergaard K, *et al.* Moderate-to-high intensity physical exercise in patients with Alzheimer’s disease: A randomized controlled trial. *J Alzheimers Dis* 2016; **50**: 443–53.

91 Hum A, Tay RY, Wong YKY, Ali NB, Leong IYO, Wu HY, *et al.* Advanced dementia: an integrated homecare programme. *BMJ Support Palliat Care* 2020; **10**: e40.

92 Hutson C, Orrell M, Dugmore O, Spector A. Sonas: a pilot study investigating the effectiveness of an intervention for people with moderate to severe dementia. *Am J Alzheimers Dis Other Demen* 2014; **29**: 696–703.

93 Jaaniste J, Linnell S, Ollerton RL, Slewa-Younan S. Drama therapy with older people with dementia-Does it improve quality of life? *Arts in Psychotherapy* 2015; **43**: 40–8.

94 Jo HK, Song E. The Effect of Reminiscence Therapy on Depression, Quality of Life, Ego-Integrity, Social Behavior Function, and Activies of Daily Living in Elderly Patients With Mild Dementia. *Educ Gerontol* 2015; **41**: 1–13.

95 Johnston B, Lawton S, McCaw C, Law E, Murray J, Gibb J, *et al.* Living well with dementia: enhancing dignity and quality of life, using a novel intervention, Dignity Therapy. *Int J Older People Nurs* 2016; **11**: 107–20.

96 Kallio EL, Ohman H, Hietanen M, Soini H, Strandberg TE, Kautiainen H, *et al.* Effects of Cognitive Training on Cognition and Quality of Life of Older Persons with Dementia. *J Am Geriatr Soc* 2018; **66**: 664–70.

97 Karefjard A, Nordgren L. Effects of dog-assisted intervention on quality of life in nursing home residents with dementia. *Scand J Occup Ther* 2019; **26**: 433–40.

98 Kelly ME, Finan S, Lawless M, Scully N, Fitzpatrick J, Quigley M, *et al.* An evaluation of community-based cognitive stimulation therapy: a pilot study with an Irish population of people with dementia. *Ir J Psychol Med* 2017; **34**: 157–67.

99 Kim D. The Effects of a Recollection-Based Occupational Therapy Program of Alzheimer’s Disease: A Randomized Controlled Trial. *Occup Ther Int* 2020; **2020**: 6305727.

100 Kim HH. Effects of experience-based group therapy on cognitive and physical functions and psychological symptoms of elderly people with mild dementia. *J Phys Ther Sci* 2015; **27**: 2069–71.

101 Kim HJ, Yang Y, Oh JG, Oh S, Choi H, Kim KH, *et al.* Effectiveness of a community-based multidomain cognitive intervention program in patients with Alzheimer’s disease. *Geriatr Gerontol Int* 2016; **16**: 191–9.

102 Kim KU, Kim SH, Oh HW. The effects of occupation-centered activity program on fall-related factors and quality of life in patients with dementia. *J Phys Ther Sci* 2017; **29**: 1188–91.

103 Kinderman P, Butchard S, Bruen AJ, Wall A, Goulden N, Hoare Z, *et al.* A randomised controlled trial to evaluate the impact of a human rights based approach to dementia care in inpatient ward and care home settings. *Health Soc Care Deliv Res* 2018; **3**: 3.

104 Knapp MJ, Knopman DS, Solomon PR, Pendlebury WW, Davis CS, Gracon SI. A 30-week randomized controlled trial of high-dose tacrine in patients with Alzheimer’s disease. The Tacrine Study Group. *JAMA* 1994; **271**: 985–91.

105 Koh WLE, Low F, Kam JW, Rahim S, Ng WF, Ng L. Person-centred creative dance intervention for persons with dementia living in the community in Singapore. *Dementia (London)* 2020; **19**: 2430–43.

106 Kohne ZAM, Nikpeyma N, Bayat F, Salsali M, Hunter P v, Kaasalainen S, *et al.* The effects of a Namaste care program on quality of life: A pilot study in Iranian women with late-stage Alzheimer’s disease. *Geriatr Nurs* 2021; **42**: 78–82.

107 Koivisto AM, Hallikainen I, Valimaki T, Hongisto K, Hiltunen A, Karppi P, *et al.* Early psychosocial intervention does not delay institutionalization in persons with mild Alzheimer disease and has impact on neither disease progression nor caregivers’ well-being: ALSOVA 3-year follow-up. *Int J Geriatr Psychiatry* 2016; **31**: 273–83.

108 Kok JS, Nielen MMA, Scherder EJA. Quality of life in small-scaled homelike nursing homes: an 8-month controlled trial. *Health Qual Life Outcomes* 2018; **16**: 1.

109 Kontos P, Miller KL, Colobong R, Palma Lazgare LI, Binns M, Low LF, *et al.* Elder-Clowning in Long-Term Dementia Care: Results of a Pilot Study. *J Am Geriatr Soc* 2016; **64**: 347–53.

110 Kumar P, Tiwari SC, Goel A, Sreenivas V, Kumar N, Tripathi RK, *et al.* Novel occupational therapy interventions may improve quality of life in older adults with dementia. *Int Arch Med* 2014; **7**: 26.

111 Kurz A, Thone-Otto A, Cramer B, Egert S, Frolich L, Gertz HJ, *et al.* CORDIAL: cognitive rehabilitation and cognitive-behavioral treatment for early dementia in Alzheimer disease: a multicenter, randomized, controlled trial. *Alzheimer Dis Assoc Disord* 2012; **26**: 246–53.

112 Laakkonen M-L, Kautiainen H, Holtta E, Savikko N, Tilvis RS, Strandberg TE, *et al.* Effects of self-management groups for people with dementia and their spouses-Randomized controlled trial. *J Am Geriatr Soc* 2016; **64**: 752–60.

113 Lai F. The Protective Impact of Telemedicine on Persons With Dementia and Their Caregivers During the COVID-19 Pandemic. *Am J Geriatr Psychiatry* 2020; **28**: 1175-84

114 Lai F-Y, Yan E-H, Tsui WS, Yu K-Y. A randomized control trial of activity scheduling for caring for older adults with dementia and its impact on their spouse care-givers. *Arch Gerontol Geriatr* 2020; **90**: 104167

115 Lam FMH, Liao LR, Kwok TCY, Pang MYC. Effects of adding whole-body vibration to routine day activity program on physical functioning in elderly with mild or moderate dementia: a randomized controlled trial. *Int J Geriatr Psychiatry* 2018; **33**: 21–30.

116 Lamb SE, Sheehan B, Atherton N, Nichols V, Collins H, Mistry D, *et al.* Dementia And Physical Activity (DAPA) trial of moderate to high intensity exercise training for people with dementia: randomised controlled trial. *BMJ* 2018; **361**: k1675.

117 Larsson V, Engedal K, Aarsland D, Wattmo C, Minthon L, Londos E. Quality of Life and the Effect of Memantine in Dementia with Lewy Bodies and Parkinson’s Disease Dementia. *Dement Geriatr Cogn Disord* 2012; **32**: 227–34.

118 Lassell R, Wood W, Schmid AA, Cross JE. A comparison of quality of life indicators during two complementary interventions: adaptive gardening and adaptive riding for people with dementia. *Complement Ther Med* 2021; **57**: 102658.

119 Latham I, Brooker D, Bray J, Jacobson-Wright N, Frost F. The Impact of Implementing a Namaste Care Intervention in UK Care Homes for People Living with Advanced Dementia, Staff and Families. *Int J Environ Res Public Health* 2020; **17**: 18.

120 Lazar A, Demiris G, Thompson HJ. Evaluation of a multifunctional technology system in a memory care unit: Opportunities for innovation in dementia care. *Inform Health Soc Care* 2016; **41**: 373–86.

121 Leroi I, Simkin Z, Hooper E, Wolski L, Abrams H, Armitage CJ, *et al.* Impact of an intervention to support hearing and vision in dementia: the SENSE-Cog Field Trial. *Int J Geriatr Psychiatry* 2020; **35**: 348‐57.

122 Leroi I, Atkinson R, Overshott R. Memantine improves goal attainment and reduces caregiver burden in Parkinson’s disease with dementia. *Int J Geriatr Psychiatry* 2014; **29**: 899–905.

123 Lin R, Chen HY, Li H, Li J. Effects of creative expression therapy on Chinese elderly patients with dementia: an exploratory randomized controlled trial. *Neuropsychiatr Dis Treat* 2019; **15**: 2171–80.

124 Liu M, Peng DZ, Cao J, Zhang WX. Predictive care improves quality of life and satisfaction in patients with AD. *Int J Clin Exp Med* 2019; **12**: 1763–70.

125 Liu SY, Shen YY, Zheng GF. Application of comprehensive nursing intervention for Alzheimer’s patients and its effects on recovery of cognitive function. *Int J Clin Exp Med* 2019; **12**: 4012–9.

126 Livingston G, Barber J, Marston L, Stringer A, Panca M, Hunter R, *et al.* Clinical and cost-effectiveness of the Managing Agitation and Raising Quality of Life (MARQUE) intervention for agitation in people with dementia in care homes: a single-blind, cluster-randomised controlled trial. *Lancet Psychiatry* 2019; **6**: 293–304.

127 Livingston G, Barber J, Rapaport P, Knapp M, Griffin M, King D, *et al.* Long-term clinical and cost-effectiveness of psychological intervention for family carers of people with dementia: a single-blind, randomised, controlled trial. *Lancet Psychiatry* 2014; **1**: 539–48.

128 Livingston G, Barber JA, Kinnunen KM, Webster L, Kyle SD, Cooper C, *et al.* DREAMS-START (Dementia RElAted Manual for Sleep; STrAtegies for RelaTives) for people with dementia and sleep disturbances: a single-blind feasibility and acceptability randomized controlled trial. *Int Psychogeriatr* 2019; **31**: 251–65.

129 Logsdon RG, Pike KC, Korte L, Goehring C. Memory Care and Wellness Services: Efficacy of Specialized Dementia Care in Adult Day Services. *Gerontologist* 2016; **56**: 318–25.

130 Logsdon RG, Pike KC, McCurry SM, Hunter P, Maher J, Snyder L, *et al.* Early-stage memory loss support groups: outcomes from a randomized controlled clinical trial. *J Gerontol B Psychol Sci Soc Sci* 2010; **65**: 691–7.

131 Lök N, Bademli K, Selçuk‐Tosun A, Selçuk-Tosun A. The effect of reminiscence therapy on cognitive functions, depression, and quality of life in Alzheimer patients: Randomized controlled trial. *Int J Geriatr Psychiatry* 2019; **34**: 47–53.

132 Lok N, Buldukoglu K, Barcin E. Effects of the cognitive stimulation therapy based on Roy’s adaptation model on Alzheimer’s patients’ cognitive functions, coping-adaptation skills, and quality of life: A randomized controlled trial. *Perspect Psychiatr Care* 2020; **56**: 581–92.

133 Lovestone S, Boadab M, Dubois B, Hull M, Rinne JO, Huppertz H-J, *et al.* A phase II trial of tideglusib in Alzheimer’s disease. *J Alzheimers Dis* 2015; **45**: 75–88.

134 Lu PH, Masterman DA, Mulnard R, Cotman C, Miller B, Yaffe K, *et al.* Effects of testosterone on cognition and mood in male patients with mild Alzheimer disease and healthy elderly men. *Arch Neurol* 2006; **63**: 177–85.

135 Machado F, Nunes P v, Viola LF, Santos FS, Forlenza O v, Yassuda MS. Quality of life and Alzheimer’s disease: Influence of participation in a rehabilitation center. *Dement Neuropsychol* 2009; **3**: 241–7.

136 Mador J, Heckec J, Clark M. Evaluation of donepezil in Alzheimer’s disease -experience from an Australian memory clinic. *Australas J Ageing* 2003; **22**: 146–50.

137 Maier F, Spottke A, Bach JP, Bartels C, Buerger K, Dodel R, *et al.* Bupropion for the Treatment of Apathy in Alzheimer Disease: A Randomized Clinical Trial. *JAMA Netw Open* 2020; **3**: e206027.

138 Marinho V, Bertrand E, Naylor R, Bomilcar I, Laks J, Spector A, *et al.* Cognitive stimulation therapy for people with dementia in Brazil (CST-Brasil): Results from a single blind randomized controlled trial. *Int J Geriatr Psychiatry* 2021; **36**: 286–93.

139 Marshall A, Spreadbury J, Cheston R, Coleman P, Ballinger C, Mullee M, *et al.* A pilot randomised controlled trial to compare changes in quality of life for participants with early diagnosis dementia who attend a “Living Well with Dementia” group compared to waiting-list control. *Aging Ment Health* 2015; **19**: 526–35.

140 Matthews DC, Ritter A, Thomas RG, Andrews RD, Lukic AS, Revta C, *et al.* Rasagiline effects on glucose metabolism, cognition, and tau in Alzheimer’s dementia. *Alzheimers Dement (N Y)* 2021; **7**: e12106.

141 McCarney R, Fisher P, Iliffe S, van Haselen R, Griffin M, van der Meulen J, *et al.* Ginkgo biloba for mild to moderate dementia in a community setting: a pragmatic, randomised, parallel-group, double-blind, placebo-controlled trial. *Int J Geriatr Psychiatry* 2008; **23**: 1222–30.

142 McGilton KS, Rochon E, Sidani S, Shaw A, Ben-David BM, Saragosa M, *et al.* Can We Help Care Providers Communicate More Effectively With Persons Having Dementia Living in Long-Term Care Homes? *Am J Alzheimers Dis Other Demen* 2017; **32**: 41–50.

143 McCarney R, Warner J, Iliffe S, van Haselen R, Griffin M, Fisher P. The Hawthorne Effect: a randomised, controlled trial. *BMC Med Res Methodol* 2007; **7**: 30.

144 Meeuwsen EJ, Melis RJF, van der Aa G, Goluke-Willemse GAM, de Leest BJM, van Raak F, *et al.* Effectiveness of dementia follow-up care by memory clinics or general practitioners: randomised controlled trial. *BMJ* 2012; **344**: e3086

145 Meguro M, Kasai M, Akanuma K, Ishii H, Yamaguchi S, Meguro K. Comprehensive approach of donepezil and psychosocial interventions on cognitive function and quality of life for Alzheimer’s disease: the Osaki-Tajiri Project. *Age Ageing* 2008; **37**: 469–73.

146 Mehling WE, Scott TM, Duffy J, Whitmer RA, Chesney MA, Boscardin WJ, *et al.* Dyadic Group Exercises for Persons with Memory Deficits and Care Partners: Mixed-Method Findings from the Paired Preventing Loss of Independence through Exercise (PLIE) Randomized Trial. *J Alzheimers Dis* 2020; **78**: 1689–706.

147 Menn P, Holle R, Kunz S, Donath C, Lauterberg J, Leidl R, *et al.* Dementia care in the general practice setting: a cluster randomized trial on the effectiveness and cost impact of three management strategies. *Value Health* 2012; **15**: 851–9.

148 Merrill CA, Jonsson MAG, Minthon L, Ejnell H, Silander HCS, Blennow K, *et al.* Vagus nerve stimulation in patients with Alzheimer’s disease: Additional follow-up results of a pilot study through 1 year. *J Clin Psychiatry* 2006; **67**: 1171–8.

149 Middelstadt J, Folkerts A-K, Blawath S, Kalbe E. Cognitive stimulation for people with dementia in long-term care facilities: Baseline cognitive level predicts cognitive gains, moderated by depression. *J Alzheimers Dis* 2016; **54**: 253–68.

150 Moyle W, Cooke M, Beattie E, Jones C, Klein B, Cook G, *et al.* Exploring the effect of companion robots on emotional expression in older adults with dementia: a pilot randomized controlled trial. *J Gerontol Nurs* 2013; **39**: 46–53.

151 Moir AR, Cassidy-Nolan D, Gough AS, Cassidy K-L. Music Therapy in Long-Term Care: Impact on Behavioural and Psychological Symptoms of Dementia and Facility Milieu. *Canadian Journal of Music Therapy* 2019; **25**: 50–9.

152 Munch M, Schmieder M, Bieler K, Goldbach R, Fuhrmann T, Zumstein N, *et al.* Bright Light Delights: Effects of Daily Light Exposure on Emotions, Restactivity Cycles, Sleep and Melatonin Secretion in Severely Demented Patients. *Curr Alzheimer Res* 2017; **14**: 1063–75.

153 Nakanishi K, Yamaga T. Effect of Instrumental Activities of Daily Living habituation due to routinising therapy in patients with frontotemporal dementia. *BMJ Case Reports* 2021; **14**: 4.

154 Nordgren L, Engstrom G. Animal-assisted intervention in dementia: effects on quality of life. *Clin Nurs Res* 2014; **23**: 7–19.

155 Nordheim J, Hausler A, Yasar S, Suhr R, Kuhlmey A, Rapp M, *et al.* Psychosocial Intervention in Couples Coping with Dementia Led by a Psychotherapist and a Social Worker: The DYADEM Trial. *J Alzheimers Dis* 2019; **68**: 745–55.

156 Novelli MMPC, Machado SCB, Lima GB, Cantatore L, Sena BP, Rodrigues RS, *et al.* Effects of the Tailored Activity Program in Brazil (TAP-BR) for Persons With Dementia: A Randomized Pilot Trial. *Alzheimer Dis Assoc Disord* 2018; **32**: 339–45.

157 Olakehinde O, Adebiyi A, Siwoku A, Mkenda S, Paddick SM, Gray WK, *et al.* Managing dementia in rural Nigeria: feasibility of cognitive stimulation therapy and exploration of clinical improvements. *Aging Ment Health* 2019; **23**: 1377–81.

158 Orgeta V, Tuijt R, Leung P, Verdaguer ES, Gould RL, Jones R, *et al.* Behavioral Activation for Promoting Well-Being in Mild Dementia: Feasibility and Outcomes of a Pilot Randomized Controlled Trial. *J Alzheimers Dis* 2019; **72**: 563–74.

159 Orrell M, Aguirre E, Spector A, Hoare Z, Woods RT, Streater A, *et al.* Maintenance cognitive stimulation therapy for dementia: single-blind, multicentre, pragmatic randomised controlled trial. *Br J Psychiatry* 2014; **204**: 454–61.

160 Orrell M, Hancock G, Hoe J, Woods B, Livingston G, Challis D. A cluster randomised controlled trial to reduce the unmet needs of people with dementia living in residential care. *Int J Geriatr Psychiatry* 2007; **22**: 1127–34.

161 Orrell M, Spector A, Thorgrimsen L, Woods B. A pilot study examining the effectiveness of maintenance Cognitive Stimulation Therapy (MCST) for people with dementia. *Int J Geriatr Psychiatry* 2005; **20**: 446–51.

162 Orrell M, Yates L, Leung P, Kang S, Hoare Z, Whitaker C, *et al.* The impact of individual Cognitive Stimulation Therapy (iCST) on cognition, quality of life, caregiver health, and family relationships in dementia: A randomised controlled trial. *PLoS Med* 2017; **14**: e1002269.

163 Padala KP, Padala PR, Lensing SY, Dennis RA, Bopp MM, Roberson PK, *et al.* Home-Based Exercise Program Improves Balance and Fear of Falling in Community-Dwelling Older Adults with Mild Alzheimer’s Disease: A Pilot Study. *J Alzheimers Dis* 2017; **59**: 565–74.

164 Paddick SM, Mkenda S, Mbowe G, Kisoli A, Gray WK, Dotchin CL, *et al.* Cognitive stimulation therapy as a sustainable intervention for dementia in sub-Saharan Africa: feasibility and clinical efficacy using a stepped-wedge design. *Int Psychogeriatr* 2017; **29**: 979–89.

165 Palm R, Trutschel D, Sorg CGG, Dichter MN, Haastert B, Holle B. Quality of Life in People With Severe Dementia and Its Association With the Environment in Nursing Homes: An Observational Study. *Gerontologist* 2019; **59**: 665–74.

166 Park J, Tolea MI, Sherman D, Rosenfeld A, Arcay V, Lopes Y, *et al.* Feasibility of Conducting Nonpharmacological Interventions to Manage Dementia Symptoms in Community-Dwelling Older Adults: A Cluster Randomized Controlled Trial. *Am J Alzheimers Dis Other Demen* 2020; **35**: 1533317519872635.

167 Park MH, Smith SC, Chrysanthaki T, Neuburger J, Ritchie CW, Hendriks AAJ, *et al.* Change in Health-related Quality of Life After Referral to Memory Assessment Services. *Alzheimer Dis Assoc Disord* 2017; **31**: 192–9.

168 Perez-Saez E, Justo-Henriques SI, Alves Apostolo JL. Multicenter randomized controlled trial of the effects of individual reminiscence therapy on cognition, depression and quality of life: Analysis of a sample of older adults with Alzheimer’s disease and vascular dementia. *Clin Neuropsychol* 2022; **36**:1975-96.

169 Pfeiffer E, Baxter D, Candelora E, Haag S, Nadiminti L, Leaverton P. Finding and treating depression in Alzheimer’s patients: A study of the effects on patients and caregivers. *Psychopharmacol Bull* 1997; **33**: 721–9.

170 Phillips MCL, Deprez LM, Mortimer GMN, Murtagh DKJ, McCoy S, Mylchreest R, *et al.* Randomized crossover trial of a modified ketogenic diet in Alzheimer’s disease. *Alzheimers Res Ther* 2021; **13**: 51.

171 Phung KTT, Waldorff FB, Buss D v, Eckermann A, Keiding N, Rishøj S, *et al.* A three-year follow-up on the efficacy of psychosocial interventions for patients with mild dementia and their caregivers: The multicentre, rater-blinded, randomised Danish Alzheimer Intervention Study (DAISY). *BMJ Open* 2013; **3**: e003584

172 Pimouguet C, le Goff M, Wittwer J, Dartigues JF, Helmer C. Benefits of Occupational Therapy in Dementia Patients: Findings from a Real-World Observational Study. *J Alzheimers Dis* 2017; **56**: 509–17.

173 Politis AM, Vozzella S, Mayer LS, Onyike CU, Baker AS, Lyketsos CG. A randomized, controlled, clinical trial of activity therapy for apathy in patients with dementia residing in long-term care. *Int J Geriatr Psychiatry* 2004; **19**: 1087–94.

174 Pongan E, Tillmann B, Leveque Y, Trombert B, Getenet JC, Auguste N, *et al.* Can musical or painting interventions improve chronic pain, mood, quality of life, and cognition in patients with mild Alzheimer’s disease? Evidence from a randomized controlled trial. *J Alzheimers Dis* 2017; **60**: 663–77.

175 Possin KL, Merrilees JJ, Dulaney S, Bonasera SJ, Chiong W, Lee K, *et al.* Effect of Collaborative Dementia Care via Telephone and Internet on Quality of Life, Caregiver Well-being, and Health Care Use: The Care Ecosystem Randomized Clinical Trial. *JAMA Intern Med* 2019; **30**: 30.

176 Pozzi C, Lanzoni A, Lucchi E, Bergamini L, Bevilacqua P, Manni B, *et al.* A pilot study of community-based occupational therapy for persons with dementia (COTID-IT Program) and their caregivers: evidence for applicability in Italy. *Aging Clin Exp Res* 2019; **31**: 1299–304.

177 Ptomey LT, Vidoni ED, Montenegro-Montenegro E, Thompson MA, Sherman JR, Gorczyca AM, *et al.* The Feasibility of Remotely Delivered Exercise Session in Adults With Alzheimer’s Disease and Their Caregivers. *J Aging Phys Act* 2019; **27**: 670–7.

278 Raglio A, Bellandi D, Baiardi P, Gianotti M, Ubezio MC, Zanacchi E, *et al.* Effect of Active Music Therapy and Individualized Listening to Music on Dementia: A Multicenter Randomized Controlled Trial. *J Am Geriatr Soc* 2015; **63**: 1534–9.

179 Raglio A, Filippi S, Leonardelli L, Trentini E, Bellandi D. The Global Music Approach to Dementia (GMA-D): evidences from a case report. *Aging Clin Exp Res* 2018; **30**: 1533–6.

180 Rapp T, Apouey BH, Senik C. The impact of institution use on the wellbeing of Alzheimer’s disease patients and their caregivers. *Soc Sci Med* 2018; **207**: 1–10.

181 Reimer MA, Slaughter S, Donaldson C, Currie G, Eliasziw M. Special care facility compared with traditional environments for dementia care: a longitudinal study of quality of life. *J Am Geriatr Soc* 2004; **52**: 1085–92.

182 Richards AG, Tietyen AC, Jicha GA, Bardach SH, Schmitt FA, Fardo DW, *et al.* Visual Arts Education improves self-esteem for persons with dementia and reduces caregiver burden: A randomized controlled trial. *Dementia* 2019; **18**: 3130–42.

183 Ridder HM, Wigram T, Ottesen AM. A pilot study on the effects of music therapy on frontotemporal dementia -- developing a research protocol. *Nord J Music Ther* 2009; **18**: 103–32.

184 Ridder HMO, Stige B, Qvale LG, Gold C. Individual music therapy for agitation in dementia: an exploratory randomized controlled trial. *Aging Ment Health* 2013; **17**: 667–78.

185 Rogers SL, Doody RS, Mohs RC, Friedhoff LT, Group and the DS. Donepezil Improves Cognition and Global Function in Alzheimer Disease: A 15-Week, Double-blind, Placebo-Controlled Study. *Arch Intern Med* 1998; **158**: 1021–31.

186 Rogers SL, Farlow MR, Doody RS, Mohs R, Friedhoff LT, Albala B, *et al.* A 24-week, double-blind, placebo-controlled trial of donepezil in patients with Alzheimer’s disease. *Neurology* 1998; **50**: 136–45.

187 Rogers SL, Friedhoff LT. The efficacy and safety of donepezil in patients with Alzheimer’s disease: results of a US Multicentre, Randomized, Double-Blind, Placebo-Controlled Trial. The Donepezil Study Group. *Dementia* 1996; **7**: 293–303.

188 Rokstad AMM, Engedal K, Kirkevold Ø, Šaltytė Benth J, Barca ML, Selbæk G, *et al.* The association between attending specialized day care centers and the quality of life of people with dementia. *Int Psychogeriatr* 2017; **29**: 627–36.

189 Rokstad AMM, Røsvik J, Kirkevold Ø, Selbaek G, Saltyte Benth J, Engedal K. The Effect of Person-Centred Dementia Care to Prevent Agitation and Other Neuropsychiatric Symptoms and Enhance Quality of Life in Nursing Home Patients: A 10-Month Randomized Controlled Trial. *Dement Geriatr Cogn Disord* 2013; **36**: 340–53.

190 Rose N, Whitworth A, Smart S, Oliver E, Cartwright J. “I remember when … ”: The impact of reminiscence therapy on discourse production in older adults with cognitive impairment. *Int J Speech Lang Pathol* 2020; **22**: 359–71.

191 Rubbi I, Magnani D, Naldoni G, di Lorenzo R, Cremonini V, Capucci P, *et al.* Efficacy of video-music therapy on quality of life improvement in a group of patients with Alzheimer’s disease: a pre-post study. *Acta Biomed* 2016; **87**: 30–7.

192 Saint-Bryant CA, Murrill J, Hayward JK, Nunez KM, Spector A. SettleIN: Using a Manualised Intervention to Facilitate the Adjustment of Older Adults with Dementia Following Placement into Residential Care. *Int J Environ Res Public Health* 2020; **17**: 10.

193 Sakakibara M, Kido M, Kuribayashi J, Okada H, Igarashi A, Kamei H, *et al.* Comparison of the Effects of a Brand-name Drug and Its Generic Drug on the Quality of Life of Alzheimer’s Disease Patients. *Clin Psychopharmacol Neurosci* 2015; **13**: 174–9.

194 Sanchez-Valdeon L, Fernandez-Martinez E, Loma-Ramos S, Lopez-Alonso AI, Darkistade EB, Ladera V. Canine-Assisted Therapy and Quality of Life in People With Alzheimer-Type Dementia: Pilot Study. *Front Psychol* 2019; **10**: 6.

195 Salazar MCR, Baez ALM, Gallego EAQ, Granada LMR. Hatha Yoga effects on Alzheimer patients (AP). *Act Colom Psicol* 2017; **20**: 139–53.

196 Santos GD, Nunes P v, Stella F, Brum PS, Yassuda MS, Ueno LM, *et al.* Multidisciplinary rehabilitation program: Effects of a multimodal intervention for patients with Alzheimer’s disease and cognitive impairment without dementia. *Arch Clin Psychiatry* 2015; **42**: 153–6.

197 Sano M, Bell K, Cote L, Dooneief G, Lawton A, Legler L, *et al.* Double-blind parallel design pilot study of acetyl levocarnitine in patients with Alzheimer’s disease. *Arch Neurol* 1992; **49**: 1137–41.

198 Schall A, Tesky VA, Adams AK, Pantel J. Art museum-based intervention to promote emotional well-being and improve quality of life in people with dementia: The ARTEMIS project. *Dementia* 2018; **17**: 728–43.

199 Sarkamo T, Tervaniemi M, Laitinen S, Numminen A, Kurki M, Johnson JK, *et al.* Cognitive, emotional, and social benefits of regular musical activities in early dementia: randomized controlled study. *Gerontologist* 2014; **54**: 634–50.

200 Schecker M, Pirnay-Dummer P, Schmidtke K, Hentrich-Hesse T, Borchardt D. Cognitive interventions in mild Alzheimer’s disease: a therapy-evaluation study on the interaction of medication and cognitive treatment. *Dement Geriatr Cogn Dis Extra* 2013; **3**: 301–11.

201 Scheltens P, Kamphuis PJGH, Verhey FRJ, Olde Rikkert MGM, Wurtman RJ, Wilkinson D, *et al.* Efficacy of a medical food in mild Alzheimer’s disease: A randomized, controlled trial. *Alzheimers Dement* 2010; **6**: 1-10.e1.

202 Shaw I, Cronje M, Shaw BS. Group-Based Exercise as a Therapeutic Strategy for the Improvement of Mental Outcomes in Mild to Moderate Alzheimer’s Patients in Low Resource Care Facilities. *Asian J Sports Med* 2021; **12**: e106593.

203 Serrani Azcurra DJ. A reminiscence program intervention to improve the quality of life of long-term care residents with Alzheimer’s disease: a randomized controlled trial. *Braz J Psychiatry*2012; **34**: 422–33.

204 Sheehan B, Lall R, Gage H, Holland C, Katz J, Mitchell K. A 12-month follow-up study of people with dementia referred to general hospital liaison psychiatry services. *Age Ageing* 2013; **42**: 786–90.

205 Shi G-X, Li Q-Q, Yang B-F, Liu Y, Guan L-P, Wu M-M, *et al.* Acupuncture for Vascular Dementia: A Pragmatic Randomized Clinical Trial. *ScientificWorldJournal* 2015; **2015**: 1–8.

206 Shi GX, Liu CZ, Li QQ, Zhu H, Wang LP. Influence of acupuncture on cognitive function and markers of oxidative DNA damage in patients with vascular dementia. *J Tradit Chin Med* 2012; **32**: 199–202.

207 Shoesmith E, Charura D, Surr C. Acceptability and feasibility study of a six-week person-centred, therapeutic visual art intervention for people with dementia. *Arts Health* 2021; **13**:296-314.

208 Silva Serelli L, Reis RC, Laks J, Pádua AC, Bottino CMC, Caramelli P. Effects of the Staff Training for Assisted Living Residences protocol for caregivers of older adults with dementia: A pilot study in the Brazilian population. *Geriatr Gerontol Int* 2017; **17**: 449–55.

209 Silva AR, Pinho MS, Macedo L, Moulin C, Caldeira S, Firmino H, *et al.* It is not only memory: effects of sensecam on improving well-being in patients with mild Alzheimer disease. *Int Psychogeriatr* 2017; **29**: 741–54.

210 Slaughter SE, Wagg AS, Jones CA, Schopflocher D, Ickert C, Bampton E, *et al.* Mobility of Vulnerable Elders study: effect of the sit-to-stand activity on mobility, function, and quality of life. *J Am Med Dir Assoc* 2015; **16**: 138–43.

211 Simoncini M, Gatti A, Quirico P, Balla S, Capellero B, Obialero R, *et al.* Acupressure in insomnia and other sleep disorders in elderly institutionalized patients suffering from Alzheimer’s disease. *Aging Clin Exp Res* 2015; **27**: 37–42.

212 Smith GS, Laxton AW, Tang-Wai DF, McAndrews MP, Diaconescu AO, Workman CI, *et al.* Increased cerebral metabolism after 1 year of deep brain stimulation in Alzheimer disease. *Arch Neurol* 2012; **69**: 1141–8.

213 Soylemez BA, Kucukguclu O, Buckwalter KC. Application of the Progressively Lowered Stress Threshold Model with Community-Based Caregivers: A Randomized Controlled Trial. *J Gerontol Nurs* 2016; **42**: 44–54.

214 Spector A, Thorgrimsen L, Woods B, Royan L, Davies S, Butterworth M, *et al.* Efficacy of an evidence-based cognitive stimulation therapy programme for people with dementia: randomised controlled trial. *Br J Psychiatry* 2003; **183**: 248–54.

215 Stanley MA, Calleo J, Bush AL, Wilson N, Snow AL, Kraus-Schuman C, *et al.* The peaceful mind program: a pilot test of a cognitive-behavioral therapy-based intervention for anxious patients with dementia. *Am J Geriatr Psychiatry* 2013; **21**: 696–708.

216 Steinberg M, Sheppard Leoutsakos JM, Podewills LJ, Lyketsos CG. Evaluation of a home-based exercise program in the treatment of Alzheimer’s disease: The Maximizing Independence in Dementia (MIND) study. *Int J Geriatr Psychiatry* 2009; **24**: 680–5.

217 Stewart DB, Berg-Weger M, Tebb S, Sakamoto M, Roselle K, Downing L, *et al.* Making a Difference: A Study of Cognitive Stimulation Therapy for Persons with Dementia. *J Gerontol Soc Work* 2017; **60**: 300–12.

218 Streater A, Spector A, Aguirre E, Orrell M. Cognitive stimulation therapy (CST) for people with dementia in practice: An observational study. *Br J Occup Ther* 2016; **79**: 762–7.

219 Subramaniam P, Woods B. Digital life storybooks for people with dementia living in care homes: an evaluation. *Clin Interv Aging* 2016; **11**: 1263–76.

220 Subramaniam P, Woods B, Whitaker C. Life review and life story books for people with mild to moderate dementia: a randomised controlled trial. *Aging Ment Health* 2014; **18**: 363–75.

221 Sultzer DL, Davis SM, Tariot PN, Dagerman KS, Lebowitz BD, Lyketsos CG, *et al.* Clinical symptom responses to atypical antipsychotic medications in Alzheimer’s disease: phase 1 outcomes from the CATIE-AD effectiveness trial. *Am J Psychiatry* 2008; **165**: 844–54.

222 Swinnen N, Vandenbulcke M, de Bruin ED, Akkerman R, Stubbs B, Firth J, *et al.* The efficacy of exergaming in people with major neurocognitive disorder residing in long-term care facilities: a pilot randomized controlled trial. *Alzheimers Res Ther* 2021; **13**: 70.

223 Tai SY, Hsu CL, Huang SW, Ma TC, Hsieh WC, Yang YH. Effects of multiple training modalities in patients with Alzheimer’s disease: a pilot study. *Neuropsychiatr Dis Treat* 2016; **12**: 2843–9.

224 Tamplin J, Clark IN, Lee YC, Baker FA. Remini-Sing: A Feasibility Study of Therapeutic Group Singing to Support Relationship Quality and Wellbeing for Community-Dwelling People Living With Dementia and Their Family Caregivers. *Front Med* 2018; **5**: 245.

225 Tanaka S, Honda S, Nakano H, Sato Y, Araya K, Yamaguchi H. Comparison between group and personal rehabilitation for dementia in a geriatric health service facility: single-blinded randomized controlled study. *Psychogeriatrics* 2017; **17**: 177–85.

226 Tay FHE, Thompson CL, Nieh CM, Nieh CC, Koh HM, Tan JJC, *et al.* Person-centered care for older people with dementia in the acute hospital. *Alzheimers Dement (N Y)* 2018; **4**: 19–27.

227 Taylor ME, Lord SR, Brodaty H, Kurrle SE, Hamilton S, Ramsay E, *et al.* A home-based, carer-enhanced exercise program improves balance and falls efficacy in community-dwelling older people with dementia. *Int Psychogeriatr* 2017; **29**: 81–91.

228 Teri L, Logsdon RG, McCurry SM, Pike KC, McGough EL. Translating an Evidence-based Multicomponent Intervention for Older Adults With Dementia and Caregivers. *Gerontologist* 2020; **60**: 548–57.

229 Telenius EW, Engedal K, Bergland A. Effect of a high-intensity exercise program on physical function and mental health in nursing home residents with dementia: an assessor blinded randomized controlled trial. *PLoS One* 2015; **10**: e0126102.

230 Testad I, Kajander M, Gjestsen MT, Dalen I. Health promotion intervention for people with early-stage dementia: A quasi-experimental study. *Brain Behav* 2020; **10**: e01888.

231 Thorgrimsen L, Schweitzer P, Orrell M. Evaluating reminiscence for people with dementia: A pilot study. *The Arts in Psychotherapy* 2002; **29**: 93–7.

232 Tietyen AC, Richards AG. A Visual Arts Education pedagogical approach for enhancing quality of life for persons with dementia (innovative practice). *Dementia* 2020; **19**: 1244–51.

233 Todri J, Lena O, Martinez Gil JL. A single blind randomized controlled trial of global postural re-education: Cognitive effects on Alzheimer disease patients. *European Journal of Psychiatry* 2019; **33**: 83–90.

234 Todri J, Lena O, Martinez Gil JL. An Experimental Pilot Study of Global Postural Reeducation Concerning the Cognitive Approach of Patients With Alzheimer’s Disease. *Am J Alzheimers Dis Other Demen* 2020; **35**: 1533317519867824.

235 Travers C. Increasing enjoyable activities to treat depression in nursing home residents with dementia: A pilot study. *Dementia* 2017; **16**: 204–18.

236 Travers C, Perkins J, Rand J, Bartlett H, Morton J. An evaluation of dog-assisted therapy for residents of aged care facilities with dementia. *Anthrozoos* 2013; **26**: 213–25.

237 Uyar F, Ozmen D, Mavioglu H, Atalay N. Assessment of the impact of dementia care and support program in both patient and caregiver outcomes: An intervention study. *Turk Geriatri Dergisi* 2019; **22**: 482–93.

238 Valenti Soler M, Aguera-Ortiz L, Olazaran Rodriguez J, Mendoza Rebolledo C, Perez Munoz A, Rodriguez Perez I, *et al.* Social robots in advanced dementia. *Front Aging Neurosci* 2015; **7**: 133.

239 Vagenas V, Vlachos GS, Vlachou N, Liakopoulos D, Kalaitzakis ME, Vikelis M. A prospective non-interventional study for evaluation of quality of life in patients with Alzheimer’s disease treated with rivastigmine transdermal patch. *SAGE Open Med* 2015; **3**: 2050312115587795.

240 van de Ven G, Draskovic I, Adang EMM, Donders R, Zuidema SU, Koopmans RTCM, *et al.* Effects of Dementia-Care Mapping on Residents and Staff of Care Homes: A Pragmatic Cluster-Randomised Controlled Trial. *PLoS One* 2013; **8**.

241 van Dam PH, Achterberg WP, Husebo BS, Caljouw MAA. Does paracetamol improve quality of life, discomfort, pain and neuropsychiatric symptoms in persons with advanced dementia living in long-term care facilities? A randomised double-blind placebo-controlled crossover (Q-PID) trial. *BMC Medicine* 2020; **18**: 407.

242 van de Ven-Vakhteeva J, Bor H, Wetzels RB, Koopmans RTCM, Zuidema SU. The impact of antipsychotics and neuropsychiatric symptoms on the quality of life of people with dementia living in nursing homes. *Int J Geriatr Psychiatry* 2013; **28**: 530–8.

243 van den Elsen GAH, Ahmed AIA, Verkes RJ, Kramers C, Feuth T, Rosenberg PB, *et al.* Tetrahydrocannabinol for neuropsychiatric symptoms in dementia: A randomized controlled trial. *Neurology* 2015; **84**: 2338–46.

244 van der Velde-van Buuringen M, Achterberg WP, Caljouw MAA. Daily garden use and quality of life in persons with advanced dementia living in a nursing home: A feasibility study. *Nurs Open* 2021; **8**: 1243-53.

245 van Haeften-van Dijk AM, Meiland FJM, Hattink BJJ, Bakker TJEM, Dröes R-M. Community day care with carer support versus usual nursing home-based day care: effects on needs, behavior, mood, and quality of life of people with dementia. *Int Psychogeriatr* 2016; **28**: 631–45.

246 van Dijk AM, van Weert JCM, Droes RM. Does theatre improve the quality of life of people with dementia? *Int Psychogeriatr* 2012; **24**: 367–81.

247 Veleva BI, Caljouw MAA, van der Steen JT, Mertens BJA, Chel VGM, Numans ME. The Effect of Ultraviolet B Irradiation Compared with Oral Vitamin D Supplementation on the Well-being of Nursing Home Residents with Dementia: A Randomized Controlled Trial. *Int J Environ Res Public Health* 2020; **17**: 5.

248 Verbeek H, Zwakhalen SM, van Rossum E, Ambergen T, Kempen GI, Hamers JP. Dementia Care Redesigned: Effects of Small-Scale Living Facilities on Residents, Their Family Caregivers, and Staff. *J Am Med Dir Assoc* 2010; **11**: 662–70.

249 Vigliotti AA, Chinchilli VM, George DR. Evaluating the Benefits of the TimeSlips Creative Storytelling Program for Persons With Varying Degrees of Dementia Severity. *Am J Alzheimers Dis Other Demen* 2019; **34**: 163–70.

250 Villar F, Celdran M, Vila-Miravent J, Fernandez E. Involving institutionalized people with dementia in their care-planning meetings: Impact on their quality of life measured by a proxy method: Innovative Practice. *Dementia* 2019; **18**: 1936–41.

251 Villars H, Dupuy C, Perrin A, Vellas B, Nourhashemi F. Impact of a therapeutic educational program on quality of life in Alzheimer’s disease: results of a pilot study. *J Alzheimers Dis* 2015; **43**: 167–76.

252 Viola LF, Nunes P v, Yassuda MS, Aprahamian I, Santos FS, Santos GD, *et al.* Effects of a multidisciplinary cognitive rehabilitation program for patients with mild Alzheimer’s disease. *Clinics (Sao Paulo, Brazil)* 2011; **66**: 1395‐1400.

253 Vroomen JM, Bosmans JE, van de Ven PM, Joling KJ, van Mierlo LD, Meiland FJM, *et al.* Community-Dwelling Patients With Dementia and Their Informal Caregivers With and Without Case Management: 2-Year Outcomes of a Pragmatic Trial. *J Am Med Dir Assoc* 2015; **16**: 800.e1-8.

254 Walter M, Hanni B, Haug M, Amrhein I, Krebs-Roubicek E, Muller-Spahn F, *et al.* Humour therapy in patients with late-life depression or Alzheimer’s disease: a pilot study. *Int J Geriatr Psychiatry* 2007; **22**: 77–83.

255 Wang GH, Wang LH, Wang C, Qin LH. Spore powder of Ganoderma lucidum for the treatment of Alzheimer disease: A pilot study. *Medicine* 2018; **97**: e0636.

256 Weintraub D, Rosenberg PB, Drye LT, Martin BK, Frangakis C, Mintzer JE, *et al.* Sertraline for the treatment of depression in Alzheimer disease: week-24 outcomes. *Am J Geriatr Psychiatry* 2010; **18**: 332–40.

257 Ward WE, Ashaye KA. An observational study of the needs and quality of life amongst patients in the treatment of Alzheimer’s dementia with cholinesterase inhibitors. *Curr Aging Sci* 2008; **1**: 140–3.

258 Wenborn J, Challis D, Head J, Miranda-Castillo C, Popham C, Thakur R, *et al.* Providing activity for people with dementia in care homes: a cluster randomised controlled trial. *Int J Geriatr Psychiatry* 2013; **28**: 1296–304.

259 Wenborn J, O’Keeffe AG, Mountain G, Moniz-Cook E, King M, Omar RZ, *et al.* Community Occupational Therapy for people with dementia and family carers (COTiD-UK) versus treatment as usual (Valuing Active Life in Dementia [VALID]) study: A single-blind, randomised controlled trial. *PLoS Med* 2021; **18**.

260 Werheid K, Schaubs B, Aguirre E, Spector A. Cognitive Stimulation Therapy: Model-Based Cultural Adaptation and Manual Translation of an Evidence-Based Psychosocial Group Therapy for People with Dementia. *GeroPsych*2021; **34**: 117-24

261 Wilks SE, Boyd PA, Bates SM, Cain DS, Geiger JR. Montessori-Based Activities Among Persons with Late-Stage Dementia: Evaluation of Mental and Behavioral Health Outcomes. *Dementia* 2019; **18**: 1373–92.

262 Wolf-Ostermann K, Worch A, Fischer T, Wulff I, Gräske J. Health outcomes and quality of life of residents of shared-housing arrangements compared to residents of special care units - results of the Berlin De We GE-study. *J Clin Nurs* 2012; **21**: 3047–60.

263 Woods RT, Orrell M, Bruce E, Edwards RT, Hoare Z, Hounsome B, *et al.* REMCARE: pragmatic Multi-Centre Randomised Trial of Reminiscence Groups for People with Dementia and their Family Carers: effectiveness and Economic Analysis. *PLoS One* 2016; **11**: e0152843.

264 Yamanaka K, Kawano Y, Noguchi D, Nakaaki S, Watanabe N, Amano T, *et al.* Effects of cognitive stimulation therapy Japanese version (CST-J) for people with dementia: a single-blind, controlled clinical trial. *Aging Ment Health* 2013; **17**: 579–86.

265 Yang B, Yang SY, Zhang YM, Liu WT, Gan Y, Li YL, *et al.* Stressor-Oriented MUlticomponent Intervention and the WeLl-Being of Patients with Alzheimer’s Disease: A Randomized Controlled Trial (SOUL-P). *J Alzheimers Dis* 2021; **79**: 141–52.

266 Yang SY, Shan CL, Qing H, Wang W, Zhu Y, Yin MM, *et al.* The Effects of Aerobic Exercise on Cognitive Function of Alzheimer’s Disease Patients. *CNS Neurol Disord Drug Targets* 2015; **14**: 1292–7.

267 Yang Y, Kwan RYC, Zhai HM, Xiong Y, Zhao T, Fang KL, *et al.* Effect of horticultural therapy on apathy in nursing home residents with dementia: a pilot randomized controlled trial. *Aging Ment Health* 2022; **26**:745-53.

268 Yasuda M, Sakakibara H. Care staff training based on person-centered care and dementia care mapping, and its effects on the quality of life of nursing home residents with dementia. *Aging Ment Health* 2017; **21**: 991–6.

269 Yokota O, Fujisawa Y, Takahashi J, Terada S, Ishihara T, Nakashima H, *et al.* Effects of group-home care on behavioral symptoms, quality of life, and psychotropic drug use in patients with frontotemporal dementia. *J Am Med Dir Assoc* 2006; **7**: 335–7.

270 Yu F, Mathiason MA, Johnson K, Gaugler JE, Klassen D. Memory matters in dementia: Efficacy of a mobile reminiscing therapy app. *Alzheimers Dement (N Y)* 2019; **5**: 644–51.

271 Yu F, Nelson NW, Savik K, Wyman JF, Dysken M, Bronas UG. Affecting cognition and quality of life via aerobic exercise in Alzheimer’s disease. *Western J Nurs Res* 2013; **35**: 24–38.

272 Yue A, Han X, Mao E, Wu G, Gao J, Huang L, *et al.* The effect of scalp electroacupuncture combined with Memantine in patients with vascular dementia: A retrospective study. *Medicine* 2020; **99**: e21242.

273 Zhang SY, Liu M. The effects of continuing care combined with music therapy on the linguistic skills, self-care, and cognitive function in Alzheimer’s disease patients. *Int J Clin Exp Med* 2020; **13**: 9621–7.

274 Zhang XH, Yu RH, Wang HL, Zheng RF. Effects of rivastigmine hydrogen tartrate and donepezil hydrochloride on the cognitive function and mental behavior of patients with Alzheimer’s disease. *Exp Ther Med* 2020; **20**: 1789–95.

275 Zhu B, Yao QP, Shao ZM, Sun LL, Ruan HL, Wang GY, *et al.* Effects of 3+1 holistic rehabilitation nursing mode on the rehabilitation and cognitive function of patients with Alzheimer’s disease. *Int J Clin Exp Med* 2020; **13**: 5645–52.

276 Zongfang Z, Wenjing L, Zhaomin C, Lei Z. Therapeutic effect of piracetam with nimodipine on vascular dementia after cerebral infarction. *Pak J Pharm Sci* 2020; **33**: 2405–11.

277 Zhang AY, Yek OPL, Lum TYS, Wong GHY, Spector A. Cultural adaptation of cognitive stimulation therapy (CST) for Chinese people with dementia: multicentre pilot study. *Int J Geriatr Psychiatry* 2018; **33**: 841–8.
