# Supplementary material for: Systematic review of the efficacy of pharmacological and non-pharmacological interventions for improving quality of life of people with dementia
Source: Br J Psychiatry. 2025 Apr 1;228(1):55–67. doi: 10.1192/bjp.2025.11 (PMC12722012; doi:10.1192/bjp.2025.11)
Supplement: Luxton et al. supplementary material 5 — Luxton et al. supplementary material [file S000712502500011Xsup005.docx]

**Supplementary material-4:** List of all studies included in the second round of the systematic review

1. Angiolillo A, Leccese D, Ciccotelli S, Di Cesare G, D'Elia K, Aurisano N, et al. Effects of Nordic walking in Alzheimer's disease: A single-blind randomized controlled clinical trial. *Heliyon* 2023; **9**(5): e15865.

2. Appel L, Appel E, Kisonas E, Lewis-Fung S, Pardini S, Rosenberg J, et al. Evaluating the Impact of Virtual Reality on the Behavioral and Psychological Symptoms of Dementia and Quality of Life of Inpatients With Dementia in Acute Care: Randomized Controlled Trial (VRCT). *J Med Internet Res* 2024; **26**: e51758.

3. Baker FA, Pac Soo V, Bloska J, Blauth L, Bukowska AA, Flynn L, et al. Home-based family caregiver-delivered music and reading interventions for people living with dementia (HOMESIDE trial): an international randomised controlled trial. *EClinicalMedicine.* 2023; **65**: 102224.

4. Bielderman A, van Corven CTM, Koopmans R, Leontjevas R, de Vugt ME, Bakker C, et al. Evaluation of the SPAN intervention for people living with young-onset dementia in the community and their family caregivers: a randomized controlled trial. *Aging & Mental Health* 2024; **28**(2): 275-84.

5. Boada M, Lopez OL, Olazaran J, Nunez L, Pfeffer M, Puente O, et al. Neuropsychological, neuropsychiatric, and quality-of-life assessments in Alzheimer's disease patients treated with plasma exchange with albumin replacement from the randomized AMBAR study. *Alzheimer's & Dementia* 2022; **18**(7): 1314-24.

6. Borges-Machado F, Teixeira L, Carvalho J, Ribeiro O. Does Multicomponent Physical Exercise Training Work for Dementia? Exploring the Effects on Cognition, Neuropsychiatric Symptoms, and Quality of Life. *J Geriatr Psychiatry Neurol.* 2023; **36**(5): 376-85.

7. Bracco L, Pinto-Carral A, Hillaert L, Mourey F. Tango-therapy vs physical exercise in older people with dementia; a randomized controlled trial. *BMC Geriatrics* 2023; **23**(1): 693.

8. Chantanachai T, Sturnieks DL, Lord SR, Close JCT, Kurrle SE, Delbaere K, et al. Effect of cognitive training on cognitive function in community-dwelling older people with mild-to-moderate dementia: A single-blind randomised controlled trial. *Australas J Ageing* 2024; **11**: 11.

9. Clarkson P, Pitts R, Islam S, Peconi J, Russell I, Fegan G, et al. Dementia Early-Stage Cognitive Aids New Trial (DESCANT) of memory aids and guidance for people with dementia: randomised controlled trial. *J Neurol Neurosurg Psychiatry* 2022; **93**(9): 1001-9.

10. Cooper C, Zabihi S, Akhtar A, Lee T, Isaaq A, Le Novere M, et al. Feasibility and acceptability of NIDUS-professional, a training and support intervention for homecare workers caring for clients living with dementia: a cluster-randomised feasibility trial. *Age & Ageing* 2024; **53**(4): 01.

11. Douma JG, Vuijk PJ, Volkers KM, Scherder EJA. Observing videos of mastication in dementia: Results of a clustered randomised controlled trial. *J Oral Rehabil* 2024; **51**(3): 546-55.

12. Fitriana LA, Darmawati I, Nasution LA, Putri ST, Rohaedi S, Anggadiredja K, et al. Effect of Centella Asiatica and Aerobic Exercise in Older Women With Dementia: A Randomized Controlled Trial. *Malaysian Journal of Medicine and Health Sciences* 2021; **17**: 153-60.

13. Foroumandi E, Javan R, Moayed L, Fahimi H, Kheirabadi F, Neamatshahi M, et al. The effects of fenugreek seed extract supplementation in patients with Alzheimer's disease: A randomized, double-blind, placebo-controlled trial. *Phytother Res* 2023; **37**(1): 285-94.

14. Han SS, White K, Cisek E. A Feasibility Study of Individuals Living at Home with Alzheimer's Disease and Related Dementias: Utilization of Visual Mapping Assistive Technology to Enhance Quality of Life and Reduce Caregiver Burden. *Clin Interv Aging* 2022; **17**: 1885-92.

15. Huang CY, Hu CJ, Huang LK, Chang EH. Effects of caregiver counselling on medication persistence and adherence in patients with dementia at a pharmacist-managed clinic: A pilot study. *J Clin Pharm Ther* 2022; **47**(12): 2074-82.

16. Inskip MJ, Mavros Y, Sachdev PS, Hausdorff JM, Hillel I, Singh MAF. Promoting independence in Lewy body dementia through exercise: the PRIDE study. *BMC Geriatrics* 2022; **22**(1): 650.

17. Jenewein J, Moergeli H, Meyer-Heim T, Muijres P, Bopp-Kistler I, Chochinov HM, et al. Feasibility, Acceptability, and Preliminary Efficacy of Dignity Therapy in Patients With Early Stage Dementia and Their Family. A Pilot Randomized Controlled Trial. *Front Psychiatry* 2021; **12**: 795813.

18. Justo-Henriques SI, Perez-Saez E, Apostolo JLA, Carvalho JO. Effectiveness of a Randomized Controlled Trial of Individual Reminiscence Therapy on Cognition, Mood and Quality of Life in Azorean Older Adults with Neurocognitive Disorders. *J Clin Med* 2021; **10**(22): 19.

19. Justo-Henriques SI, Perez-Saez E, Marques-Castro AE, Carvalho JO. Effectiveness of a year-long individual cognitive stimulation program in Portuguese older adults with cognitive impairment. *Neuropsychol Dev Cogn B Aging Neuropsychol Cogn* 2023; **30**(3): 321-35.

20. Kor PPK, Parial LL, Yu CTK, Liu JYW, Liu DPM, Hon JMK. Effects of a Family Caregiver-Delivered MultiSensory Cognitive Stimulation Intervention for Older People With Dementia During Coronavirus 2019: A Randomized Controlled Trial. *Gerontologist* 2024; **64**(2): 01.

21. Koskas P, Kohler S, Estrada J, Sebbagh M, Lacaille S, Lilamand M. Effect of a multi-domain intervention on the quality of life in older adults with major neurocognitive disorder: A pilot study. *Revue Neurologique* 2022; **178**(4): 355-62.

22. Kosters J, Janus SIM, van den Bosch KA, Andringa TC, Hoop EO, de Boer MR, et al. Soundscape Awareness Intervention Reduced Neuropsychiatric Symptoms in Nursing Home Residents With Dementia: A Cluster-Randomized Trial With MoSART. *J Am Med Dir Assoc* 2023; **24**(2): 192-8.e5.

23. Kratzer A, Diehl K, Gefeller O, Meyer S, Graessel E. Non-pharmacological, psychosocial MAKS-s intervention for people with severe dementia in nursing homes: results of a cluster-randomised trial. *BMC Geriatrics* 2022; **22**(1): 1001.

24. Kwon MH, Kim SK. Effects of Client-Centered Occupational Therapy on Behavioral Psychological Symptoms, Social Interaction, Occupational Performance, Quality of Life, and Caregiver Burden among the Individuals with Dementia. *Occup Ther Health Care* 2023; **37**(2): 266-81.

25. Leach MJ, Sangalli M, Breakspear I, Walsh S. Essential oils for agitation in dementia [rELOAD]: A pragmatic, cluster-randomized, placebo-controlled, pilot feasibility trial. *Integr Med Res* 2021; **10**(4): 100747.

26. Lech S, Gellert P, Spang RP, Voigt-Antons JN, Huscher D, O'Sullivan JL, et al. Effectiveness of a tablet-based intervention for people living with dementia in primary care-A cluster randomized controlled trial. *Int J Geriatr Psychiatry* 2023; **38**(12): e6035.

27. Lin LW, Lu YH, Chang TH, Yeh SH. Effects of Drama Therapy on Depressive Symptoms, Attention, and Quality of Life in Patients With Dementia. *J Nurs Res* 2021; **30**(1): e188.

28. McDermid J, Henley W, Corbett A, Williams G, Fossey J, Clare L, et al. Impact of the iWHELD digital person-centered care program on quality of life, agitation and psychotropic medications in people with dementia living in nursing homes during the COVID-19 pandemic: A randomized controlled trial. *Alzheimer's & Dementia* 2024; **20**(3): 1797-806.

29. Monin JK, Oettingen G, Laws H, David D, DeMatteo L, Marottoli R. A Controlled Pilot Study of the Wish Outcome Obstacle Plan Strategy for Spouses of Persons With Early-Stage Dementia. *J Gerontol B Psychol Sci Soc Sci* 2022; **77**(3): 513-24.

30. Mountain GA, Cooper CL, Wright J, Walters SJ, Lee E, Craig C, et al. The Journeying through Dementia psychosocial intervention versus usual care study: a single-blind, parallel group, phase 3 trial. *Lancet Healthy Longev* 2022; **3**(4): e276-e85.

31. Noone D, Payne J, Stott J, Aguirre E, Patel-Palfreman MM, Stoner C, et al. The Feasibility of a Mindfulness Intervention for Depression in People with Mild Dementia: A Pilot Randomized Controlled Trial. *Clin Gerontol* 2023; **46**(3): 346-58.

32. Prick AJC, Zuidema SU, van Domburg P, Verboon P, Vink AC, Schols J, et al. Effects of a music therapy and music listening intervention for nursing home residents with dementia: a randomized controlled trial. *Front Med (Lausanne)* 2024; **11**: 1304349.

33. Rai HK, Schneider J, Orrell M. An Individual Cognitive Stimulation Therapy App for People with Dementia and Carers: Results from a Feasibility Randomized Controlled Trial (RCT). *Clin Interv Aging* 2021; **16**: 2079-94.

34. Rogers CL, Lageman SK, Fontanesi J, Wilson GD, Boling PA, Bansal S, et al. Low-Dose Whole Brain Radiation Therapy for Alzheimer's Dementia: Results From a Pilot Trial in Humans. *Int J Radiat Oncol Biol Phys* 2023; **117**(1): 87-95.

35. Saitoh Y, Hosomi K, Mano T, Takeya Y, Tagami S, Mori N, et al. Randomized, sham-controlled, clinical trial of repetitive transcranial magnetic stimulation for patients with Alzheimer’s dementia in Japan. *Front Aging Neurosci* 2022; **14**.

36. Schüssler S, Zuschnegg J, Paletta L, Lodron G, Steiner J, Pansy-Resch S, et al. Effects of coach robot pepper versus tablet training on psychosocial and physical outcomes of persons with dementia: A mixed-methods study. *Alzheimers Dement* 2021; **17**: e053453.

37. Shyu YIL, Lin CC, Kwok YT, Shyu HY, Kuo LM. A community‐based computerised cognitive training program for older persons with mild dementia: A pilot study. *Australas J Ageing* 2022; **41**(1): e82-e93.

38. Spector A, Abdul Wahab ND, Stott J, Fisher E, Hui EK, Perkins L, et al. Virtual group Cognitive Stimulation Therapy (vCST) for dementia: mixed methods feasibility randomised controlled trial. *Gerontologist* 2024; **6**: 06.

39. Thumuluri D, Lyday R, Babcock P, Ip EH, Kraft RA, Laurienti PJ, et al. Improvisational Movement to Improve Quality of Life in Older Adults With Early-Stage Dementia: A Pilot Study. *Front Sports Act Living* 2021; **3**: 796101.

40. Timler A, Bulsara C, Bulsara M, Vickery A, Jacques A, Codde J. Examining the use of cannabidiol and delta-9-tetrahydrocannabinol-based medicine among individuals diagnosed with dementia living within residential aged care facilities: Results of a double-blind randomised crossover trial. *Australas J Ageing* 2023; **42**(4): 698-709.

41. Torres-Castro S, Rabaneda-Bueno R, Lopez-Ortega M, Gutierrez-Robledo LM, Guzman A. Multicomponent Staff Training Intervention to Improve Residential Dementia Care (PROCUIDA-Demencia): A Mixed-Methods 2-Arm Cluster Randomized Controlled Pilot and Clinical Outcomes Study. *J Am Med Dir Assoc* 2022; **23**(3): 350-8.e5.

42. van Santen J, Meiland FJM, Droes RM, van Straten A, Bosmans JE. Cost-effectiveness of exergaming compared to regular day-care activities in dementia: Results of a randomised controlled trial in The Netherlands. *Health Soc Care Community* 2022; **30**(5): e1794-e804.

43. Villars H, Cantet C, de Peretti E, Perrin A, Soto-Martin M, Gardette V. Impact of an educational programme on Alzheimer's disease patients' quality of life: results of the randomized controlled trial THERAD. *Alzheimers Res Ther* 2021; **13**(1): 152.

44. Vu HT, Nguyen HT, Nguyen AT. Effectiveness of Non-Pharmacological Interventions for Dementia among the Elderly: A Randomized Controlled Trial. *Geriatrics* 2024; **9**(2): 18.

45. Yang L, Xuan C, Yu C, Jin X, Zheng P, Yan J. Effects of comprehensive intervention on life quality among the elderly with Alzheimer Disease and their caregivers based on mixed models. *Nurs Open* 2022; **9**(2): 1412-22.

46. Yous ML, Hunter PV, Coker E, Fisher KA, Nicula M, Kazmie N, et al. Feasibility and Effects of Namaste Care for Persons with Advanced Dementia in Canadian Long-Term Care Homes. *J Am Med Dir Assoc* 2023; **24**(9): 1433-8.e5.

47. Yuan L, Ye J, Wang W, Xiao A, Zhou Y, Lin W, et al. Research of Arithmetic and Drawing Writing in Improving Communication and Cognitive Function in Patients with Mild-to-Moderate Dementia: A Cluster Randomized Controlled Trial. *Alpha Psychiatry* 2024; **25**(2): 262-8.
