# Supplementary material for: Systematic review of the efficacy of pharmacological and non-pharmacological interventions for improving quality of life of people with dementia
Source: Br J Psychiatry. 2025 Apr 1;228(1):55–67. doi: 10.1192/bjp.2025.11 (PMC12722012; doi:10.1192/bjp.2025.11)
Supplement: Luxton et al. supplementary material 6 — Luxton et al. supplementary material [file S000712502500011Xsup006.docx]

| **First author name**  **Supplementary material-5:** Quality assessment of all studies included in the first round of the systematic review. | **Year of publication** | **Intervention studied** | **Selection bias** | **Study design** | **Confounders** | **Blinding** | **Data collection method** | **Withdrawals and dropouts** | **Global quality assessment rating** |
| --- | --- | --- | --- | --- | --- | --- | --- | --- | --- |
| Abd El- Kader | 2016 | Treadmill aerobic exercise | 2 | 1 | 3 | 3 | 1 | 2 | 3 |
| Abd El- Kader | 2011 | Aerobic walking exercise training and upper limbs exercises | 3 | 1 | 3 | 3 | 1 | 3 | 3 |
| Adrait | 2017 | Hearing Aids | 2 | 1 | 1 | 1 | 1 | 2 | 1 |
| Aguiar | 2014 | Rivastigmine patch combined with physical exercise vs. Rivastigmine patch alone | 2 | 1 | 1 | 2 | 1 | 2 | 1 |
| Aguirre | 2013 | Cognitive stimulation therapy | 1 | 2 | 2 | 3 | 1 | 1 | 2 |
| Aisen | 2003 | Rofecoxib vs. naproxen | 1 | 1 | 1 | 1 | 1 | 2 | 1 |
| Alvares-Pereira | 2020 | Cognitive Stimulation Therapy | 2 | 1 | 1 | 2 | 1 | 1 | 1 |
| Amieva | 2016 | Cognitive training vs. reminiscence therapy vs. an individualised cognitive rehabilitation program | 2 | 1 | 3 | 2 | 1 | 2 | 2 |
| Astell | 2018 | Computer interactive reminiscence and conversation aid groups (CIRCA) | 3 | 2 | 2 | 2 | 1 | 1 | 2 |
| Avila | 2004 | Neuropsychological rehabillitation | 3 | 2 | 2 | 2 | 3 | 3 | 3 |
| Ballard | 2002 | Melissa Aromatherapy | 1 | 1 | 3 | 1 | 1 | 1 | 2 |
| Bergh | 2012 | Discontinuing antidepressants | 2 | 1 | 1 | 1 | 1 | 1 | 1 |
| Berk | 2019 | Mindfulness-based intervention for persons with dementia and their caregivers | 3 | 2 | 2 | 2 | 1 | 3 | 3 |
| Binns | 2020 | Cognitive stimulation therapy combined with a fall prevention exercise (CogEx) | 2 | 1 | 3 | 2 | 1 | 2 | 2 |
| Birkenhäger-Gillesse | 2020 | Caregiver training intervention | 2 | 1 | 3 | 2 | 1 | 2 | 2 |
| Boersma | 2019 | The Veder Contact Method: A Theatre-Based Communication Method | 3 | 1 | 1 | 3 | 1 | 2 | 3 |
| Borges-Machado | 2019 | Multicomponent Exercise Intervention | 3 | 1 | 3 | 2 | 1 | 1 | 3 |
| Bottini | 1992 | Oxiracetam | 1 | 1 | 1 | 1 | 2 | 1 | 1 |
| Brodaty | 2004 | Making Memories program comprised of discussion and behavioural modification components and ongoing support | 3 | 2 | 2 | 2 | 1 | 3 | 3 |
| Bromundt | 2019 | Dawn-dusk simulation | 2 | 2 | 1 | 2 | 1 | 3 | 2 |
| Brooker | 2017 | Implementation of the Meeting Centres Support Program | 3 | 2 | 1 | 2 | 1 | 2 | 2 |
| Burns | 1999 | Donepezil 5mg vs. 10mg | 3 | 1 | 1 | 1 | 3 | 1 | 3 |
| Camic | 2014 | A multi-session art-gallery-based intervention | 2 | 2 | 3 | 2 | 1 | 1 | 2 |
| Cao | 2020 | Donepezil plus memantine vs. donepezil alone | 1 | 2 | 1 | 2 | 1 | 1 | 1 |
| Capotosto | 2017 | Cognitive Stimulation Therapy | 2 | 2 | 1 | 1 | 1 | 1 | 1 |
| Caramelli | 2014 | Galantamine vs. galantamine combined with nimodipine | 2 | 1 | 2 | 2 | 1 | 3 | 2 |
| Carbone | 2021 | Cognitive Stimulation Therapy | 2 | 1 | 1 | 2 | 1 | 1 | 1 |
| Chapman | 2004 | Cognitive-communication Stimulation plus Donepezil | 2 | 1 | 1 | 2 | 1 | 1 | 1 |
| Charlesworth | 2016 | Peer support and reminiscence therapy | 2 | 1 | 1 | 2 | 1 | 1 | 1 |
| Charras | 2020 | Dance intervention | 3 | 2 | 2 | 2 | 1 | 3 | 3 |
| Charras | 2013 | Wearing uniform | 2 | 2 | 2 | 2 | 1 | 2 | 1 |
| Chaudhry | 2020 | Culturally adapted, group-based Montessori intervention | 3 | 2 | 2 | 2 | 1 | 1 | 2 |
| Chen, K | 2020 | Humanoid Companion Robot | 2 | 1 | 1 | 2 | 1 | 1 | 1 |
| Chen, X | 2020 | Traditional Opera | 2 | 1 | 1 | 2 | 1 | 1 | 1 |
| Chenoweth | 2009 | Person-centred care vs. dementia-care mapping vs. usual care | 2 | 1 | 1 | 2 | 1 | 1 | 1 |
| Chenoweth | 2007 | Dementia Care Mapping | 2 | 2 | 2 | 2 | 1 | 1 | 1 |
| Chenoweth | 2014 | Person-centered residential care and environment | 2 | 1 | 2 | 2 | 1 | 2 | 1 |
| Cheon | 2008 | Memantine | 1 | 2 | 2 | 2 | 1 | 3 | 2 |
| Cheston | 2016 | ‘‘Living Well with Dementia’’ (LivDem) model of group support | 3 | 2 | 2 | 2 | 1 | 2 | 2 |
| Chew | 2015 | Multimodal cognitive and physical rehabilitation program | 2 | 2 | 2 | 2 | 1 | 3 | 2 |
| Cho | 2018 | Music therapy (singing group) | 2 | 1 | 1 | 2 | 1 | 2 | 1 |
| Chu | 2020 | Multifaceted walking intervention | 2 | 2 | 1 | 2 | 1 | 1 | 1 |
| Chung | 2009 | Intergenerational reminiscence programme | 3 | 2 | 2 | 2 | 1 | 1 | 2 |
| Churcher | 2017 | An adapted mindfulness intervention | 2 | 1 | 1 | 2 | 1 | 2 | 1 |
| Clare | 2019 | Individual goal‐oriented cognitive rehabilitation | 2 | 1 | 1 | 2 | 1 | 1 | 1 |
| Coelho | 2020 | Promoting Reminiscences with Virtual Reality Headsets | 2 | 2 | 1 | 3 | 3 | 3 | 3 |
| Coen | 2011 | Cognitive Stimulation Therapy | 2 | 1 | 3 | 2 | 1 | 1 | 2 |
| Collins | 2018 | Compassion-focused therapy group | 2 | 2 | 2 | 2 | 1 | 2 | 1 |
| Collins | 2020 | Indoor sensory garden | 2 | 2 | 2 | 2 | 1 | 1 | 1 |
| Cornelis | 2018 | Multicomponent rehabilitation programme | 2 | 2 | 2 | 2 | 1 | 1 | 1 |
| Cove | 2014 | Once weekly CST plus carer training program | 2 | 1 | 1 | 2 | 1 | 1 | 1 |
| Craig | 2018 | Compassion focused therapy | 2 | 2 | 3 | 2 | 1 | 1 | 2 |
| Cunningham | 2019 | Reminiscence Music with a Mobile App (Memory Tracks) | 3 | 2 | 3 | 2 | 1 | 3 | 3 |
| D'Cunha | 2019 | Art Gallery intervention | 2 | 2 | 2 | 2 | 1 | 1 | 1 |
| D'Onofrio | 2019 | MARIO service companion robot | 3 | 2 | 1 | 2 | 1 | 3 | 3 |
| Davis | 2001 | Cognitive intervention consisting of training in face-name associations, spaced retrieval, and cognitive stimulation | 3 | 1 | 1 | 2 | 3 | 1 | 3 |
| De Rooij | 2012 | Traditional vs. small-scale long-term care settings | 3 | 2 | 2 | 2 | 1 | 3 | 3 |
| De Vocht | 2015 | One-to-one 30-min individualised interaction per day | 3 | 2 | 2 | 2 | 1 | 3 | 3 |
| Doody | 2016 | Dextromethorphan/quinidine | 2 | 2 | 2 | 3 | 2 | 2 | 2 |
| Doody | 2013 | Semagacestat | 2 | 1 | 1 | 1 | 1 | 1 | 1 |
| Duff | 2007 | Hypnosis vs. discussion therapy | 3 | 1 | 2 | 2 | 3 | 3 | 3 |
| Edwards | 2013 | Therapeutic Garden | 3 | 2 | 2 | 2 | 1 | 1 | 2 |
| El Alili | 2020 | Namaste Care intervention | 3 | 1 | 1 | 2 | 1 | 3 | 3 |
| Enette | 2020 | Continuous vs. interval aerobic training | 2 | 1 | 1 | 2 | 1 | 1 | 1 |
| Fan | 2020 | Combination of donepezil with hyperbaric oxygen therapy and functional rehabilitation training | 3 | 1 | 2 | 2 | 1 | 3 | 3 |
| Ferrer | 2014 | Rehabilitation program (cognitive stimulation therapy) | 3 | 2 | 2 | 2 | 1 | 1 | 2 |
| Fialho | 2012 | Cognitive-behavioural intervention program | 3 | 2 | 2 | 2 | 1 | 1 | 2 |
| Fontaine | 2003 | Olanzapine vs. Risperidone | 2 | 1 | 2 | 1 | 1 | 1 | 1 |
| Garrido | 2020 | Music Playlists (music therapy) | 1 | 1 | 2 | 2 | 1 | 2 | 1 |
| Gault | 2015 | 𝛂7 agonist ABT-126 | 2 | 1 | 1 | 1 | 1 | 1 | 1 |
| Gavrilova | 2008 | 10/66 caregiver intervention - In Russia | 1 | 1 | 1 | 2 | 1 | 1 | 1 |
| Gibbor | 2020 | Individual Cognitive Stimulation Therapy (iCST) | 2 | 1 | 1 | 2 | 1 | 1 | 1 |
| Goyder | 2012 | Staff Training in Assisted Living Residences (STAR) program | 3 | 2 | 2 | 2 | 1 | 3 | 3 |
| Graff | 2007 | Occupational Therapy program | 2 | 1 | 2 | 2 | 1 | 2 | 1 |
| Green | 2009 | Tarenflurbil | 2 | 1 | 1 | 1 | 1 | 2 | 1 |
| Gresham | 2018 | A program that combines caregiver training with a residential respite stay | 2 | 2 | 1 | 2 | 1 | 3 | 2 |
| Gridley | 2016 | Life story work | 3 | 2 | 3 | 2 | 1 | 3 | 3 |
| Guerra | 2011 | 10/66 caregiver intervention - In Peru | 2 | 1 | 1 | 2 | 1 | 1 | 1 |
| Guerriero | 2015 | Emisymmetric bilateral stimulation | 2 | 2 | 2 | 2 | 1 | 1 | 1 |
| Gustafsson | 2015 | Robotic cat intervention | 3 | 2 | 2 | 3 | 1 | 1 | 3 |
| Halek | 2020 | Two types of dementia-specific case conferences | 1 | 2 | 1 | 2 | 1 | 3 | 2 |
| Hamill | 2012 | ‘Dancing down memory lane’: Circle dancing psychotherapeutic intervention | 3 | 2 | 2 | 2 | 1 | 1 | 2 |
| Hammarlund | 2021 | Affordable robotic pet ownership | 3 | 2 | 3 | 2 | 1 | 1 | 3 |
| Hattori | 2011 | Art therapy | 2 | 1 | 1 | 2 | 1 | 1 | 1 |
| Henskens | 2017 | Movement-oriented restorative care | 2 | 2 | 1 | 2 | 1 | 1 | 1 |
| Henskens | 2018 | Movement stimulating interventions | 2 | 1 | 2 | 2 | 1 | 2 | 1 |
| Hindle | 2018 | Goal-orientated cognitive rehabilitation | 2 | 1 | 1 | 2 | 1 | 1 | 1 |
| Hoffmann | 2016 | Moderate-to-high intensity aerobic exercise program | 2 | 1 | 1 | 2 | 1 | 1 | 1 |
| Hum | 2020 | Integrated palliative homecare programme | 2 | 2 | 2 | 2 | 1 | 3 | 2 |
| Hutson | 2014 | Sonas, a group intervention involving multisensory stimulation, reminiscence, and light physical activity | 2 | 1 | 1 | 2 | 1 | 1 | 1 |
| Jaaniste | 2015 | Drama Therapy | 3 | 2 | 1 | 2 | 1 | 2 | 2 |
| Jo | 2015 | Reminiscence therapy | 3 | 2 | 2 | 2 | 3 | 2 | 3 |
| Johnstone | 2016 | Dignity Therapy | 3 | 2 | 2 | 2 | 3 | 1 | 3 |
| Kallio | 2018 | Cognitive training | 2 | 1 | 1 | 2 | 3 | 1 | 2 |
| Karefjard | 2019 | Dog-assisted intervention | 3 | 2 | 1 | 2 | 1 | 3 | 3 |
| Kelly | 2017 | Community-based Cognitive Stimulation Therapy | 3 | 2 | 2 | 2 | 1 | 3 | 3 |
| Kim | 2017 | Occupation centred activity program | 3 | 1 | 1 | 2 | 1 | 3 | 3 |
| Kim | 2016 | Community-based multidomain cognitive intervention program | 1 | 1 | 1 | 2 | 1 | 2 | 1 |
| Kim | 2015 | Experience-based group therapy | 3 | 2 | 2 | 2 | 1 | 3 | 3 |
| Kim | 2020 | Recollection-Based Occupational Therapy | 2 | 1 | 1 | 2 | 1 | 3 | 2 |
| Kinderman | 2018 | Human rights based approach intervention | 2 | 1 | 1 | 2 | 1 | 1 | 1 |
| Knapp | 1994 | High dose Tacrine | 3 | 1 | 1 | 1 | 3 | 3 | 3 |
| Koh | 2020 | Person-centred creative dance intervention | 2 | 2 | 2 | 2 | 1 | 1 | 1 |
| Kohne | 2021 | Namaste Care intervention | 3 | 2 | 2 | 2 | 1 | 1 | 2 |
| Koivisto | 2017 | Early psychosocial intervention | 1 | 1 | 1 | 2 | 1 | 3 | 2 |
| Kok | 2018 | Small-scaled homelike nursing homes | 2 | 2 | 1 | 3 | 1 | 3 | 3 |
| Kontos | 2016 | Elder-clowning | 2 | 2 | 2 | 2 | 1 | 1 | 1 |
| Kumar | 2014 | Novel occupational therapy regimen | 2 | 1 | 1 | 2 | 1 | 1 | 1 |
| Kurz | 2012 | CORDIAL: Cognitive Rehabilitation and Cognitive-behavioral Treatment | 2 | 1 | 2 | 2 | 1 | 1 | 1 |
| Laakkonen | 2016 | Self-management groups for people with dementia and their spouses | 2 | 1 | 1 | 2 | 3 | 1 | 2 |
| Lai | 2020 | Weekly health services delivered through video communication apps | 2 | 2 | 3 | 2 | 1 | 3 | 3 |
| Lai | 2020 | Activity scheduling | 3 | 1 | 1 | 2 | 1 | 1 | 2 |
| Lam | 2018 | Whole-body vibration (WBV) added to a routine activity program | 2 | 1 | 1 | 2 | 1 | 1 | 1 |
| Lamb | 2018 | Moderate to high intensity exercise training | 3 | 1 | 1 | 2 | 1 | 1 | 2 |
| Larsson | 2012 | Memantine | 2 | 1 | 1 | 1 | 1 | 1 | 1 |
| Latham | 2020 | Namaste Care intervention | 3 | 2 | 2 | 2 | 1 | 1 | 2 |
| Lazar | 2016 | Multifunctional technology system | 3 | 2 | 2 | 3 | 2 | 1 | 3 |
| Leroi | 2020 | Intervention to support hearing and vision in dementia | 2 | 2 | 1 | 3 | 1 | 2 | 2 |
| Leroi | 2014 | 20 mg Memantine | 2 | 1 | 1 | 1 | 1 | 1 | 1 |
| Lin | 2019 | Creative expression therapy | 2 | 1 | 1 | 3 | 1 | 1 | 2 |
| Liu | 2019 | Comprehensive nursing intervention | 3 | 1 | 1 | 2 | 1 | 1 | 2 |
| Liu | 2019 | Predictive nursing care program | 3 | 1 | 1 | 2 | 1 | 1 | 2 |
| Livingstone | 2019 | Dementia RElAted Manual for Sleep; STrAtegies for RelaTives intervention (DREAMS START) | 2 | 1 | 1 | 2 | 1 | 1 | 1 |
| Livingstone | 2014 | STrAtegies for RelaTives psychological intervention (START) | 2 | 1 | 1 | 2 | 1 | 1 | 1 |
| Livingstone | 2019 | Managing Agitation and Raising Quality of Life (MARQUE) intervention | 2 | 1 | 1 | 2 | 1 | 1 | 1 |
| Logsdon | 2010 | Time-limited early-stage memory loss (ESML) support group program | 2 | 1 | 1 | 2 | 1 | 1 | 1 |
| Logsdon | 2016 | Program of specialized dementia adult day services | 2 | 2 | 1 | 2 | 1 | 3 | 2 |
| Lok | 2020 | Cognitive stimulation therapy based on Roy’s adaptation model | 2 | 1 | 1 | 2 | 1 | 1 | 1 |
| Lok | 2019 | Reminiscence therapy | 2 | 1 | 1 | 2 | 1 | 1 | 1 |
| Lovestone | 2015 | Tideglusib | 2 | 1 | 1 | 1 | 1 | 1 | 1 |
| Lu | 2006 | Testosterone | 2 | 1 | 1 | 1 | 1 | 2 | 1 |
| Machado | 2009 | Cognitive and functional rehabilitation program | 3 | 2 | 2 | 2 | 1 | 1 | 2 |
| Mador | 2003 | Cholinesterase inhibitor use (specifically Donepezil) | 2 | 2 | 2 | 2 | 1 | 1 | 1 |
| Maier | 2020 | Bupropion | 2 | 1 | 1 | 1 | 1 | 2 | 1 |
| Marinho | 2020 | CST | 2 | 1 | 1 | 1 | 1 | 1 | 1 |
| Marshall | 2015 | ‘Living Well with Dementia’ group vs. waiting-list control | 3 | 1 | 1 | 2 | 1 | 1 | 2 |
| Matthews | 2021 | Rasagiline | 2 | 1 | 1 | 1 | 1 | 2 | 1 |
| McCarney | 2007 | Minimal follow-up vs. intensive follow-up in participants in a placebo controlled trial of Ginkgo biloba | 2 | 1 | 1 | 3 | 1 | 1 | 2 |
| McCarney | 2008 | Ginkgo biloba | 2 | 1 | 1 | 1 | 1 | 1 | 1 |
| McGilton | 2017 | Communication intervention | 2 | 2 | 1 | 2 | 1 | 1 | 1 |
| Meeuwsen | 2013 | Follow-up care by memory clinics vs. General Practitioners | 2 | 2 | 1 | 2 | 1 | 1 | 1 |
| Meguro | 2008 | Donepezil combined with reminiscence therapy, Japanese caligraphy sessions and promotion of cooking small Japanese dishes. | 2 | 1 | 3 | 2 | 1 | 1 | 2 |
| Mehling | 2020 | Dyadic Group Exercises: Preventing Loss of Independence through Exercise(PLIE ́) | 2 | 1 | 2 | 2 | 1 | 2 | 1 |
| Menn | 2012 | Complex nonpharmacological intervention including actively approaching counseling and caregiver support groups with differing intensity | 2 | 1 | 2 | 2 | 1 | 1 | 1 |
| Merrill | 2006 | Vagus nerve stimulation | 2 | 2 | 2 | 3 | 1 | 1 | 2 |
| Middelstadt | 2016 | Cognitive Stimulation Therapy | 2 | 1 | 1 | 2 | 1 | 2 | 1 |
| Moir | 2019 | Music Therapy | 2 | 2 | 2 | 2 | 1 | 1 | 1 |
| Moyle | 2013 | Companion robots (PARO) vs. participation in an interactive reading group | 2 | 1 | 2 | 3 | 1 | 1 | 2 |
| Munch | 2017 | Daily Light Exposure | 2 | 2 | 3 | 2 | 1 | 3 | 3 |
| Nordgren | 2014 | Canine-assisted therapy | 2 | 2 | 2 | 2 | 1 | 3 | 2 |
| Nordheim | 2019 | Psychosocial intervention in couples coping with Dementia | 2 | 1 | 1 | 2 | 1 | 2 | 1 |
| Novelli | 2018 | Tailored Activity program | 3 | 1 | 1 | 2 | 1 | 1 | 2 |
| Olakehinde | 2018 | Cognitive Stimulation Therapy | 3 | 2 | 2 | 2 | 1 | 1 | 2 |
| Orgeta | 2019 | Behavioural Activation | 2 | 1 | 1 | 2 | 1 | 1 | 1 |
| Orrell | 2017 | Individual Cognitive Stimulation Therapy | 2 | 1 | 1 | 2 | 1 | 1 | 1 |
| Orrell | 2005 | Maintenance Cognitive Stimulation Therapy (CST) | 3 | 1 | 1 | 2 | 1 | 1 | 2 |
| Orrell | 2007 | 1 hour per week liaison input to deliver a personalised intervention package | 2 | 1 | 2 | 2 | 1 | 1 | 1 |
| Orrell | 2014 | Maintenance Cognitive Stimulation Therapy | 2 | 1 | 1 | 2 | 1 | 1 | 1 |
| Padala | 2017 | Home-Based Exercise Program | 2 | 1 | 1 | 3 | 1 | 1 | 2 |
| Paddick | 2017 | Cognitive Stimulation Therapy | 2 | 2 | 3 | 2 | 1 | 1 | 2 |
| Palm | 2019 | Various types of dementia care units: Large integrated, large segregated, small integrated, small segregated. | 2 | 2 | 1 | 2 | 1 | 1 | 1 |
| Park | 2017 | Referral to memory assessment services | 1 | 2 | 1 | 2 | 1 | 2 | 1 |
| Park | 2020 | Chair yoga vs. music intervention vs. chair-based exercise | 2 | 1 | 2 | 2 | 1 | 1 | 1 |
| Pérez-Sáez | 2021 | Individual reminiscence therapy | 3 | 1 | 1 | 2 | 1 | 1 | 2 |
| Pfeiffer | 1997 | Antidepressant medication | 3 | 2 | 3 | 2 | 1 | 1 | 3 |
| Phillips | 2021 | Modified ketogenic diet | 3 | 1 | 2 | 2 | 1 | 1 | 2 |
| Phung | 2013 | Early psychosocial counselling and support programme | 2 | 1 | 1 | 2 | 1 | 2 | 1 |
| Pimouguet | 2017 | Occupational Therapy program | 1 | 2 | 2 | 2 | 1 | 2 | 1 |
| Politis | 2004 | Kit-based activity intervention | 2 | 1 | 3 | 2 | 1 | 1 | 2 |
| Pongan | 2017 | Music therapy vs. painting therapy | 2 | 1 | 2 | 2 | 1 | 1 | 1 |
| Possin | 2019 | Collaborative dementia care via telephone and internet | 3 | 1 | 1 | 2 | 1 | 1 | 2 |
| Pozzi | 2018 | Community-based occupational therapy | 2 | 2 | 2 | 2 | 1 | 1 | 1 |
| Ptomey | 2019 | Remotely delivered exercise session | 1 | 2 | 2 | 2 | 1 | 1 | 1 |
| Raglio | 2015 | Active music therapy and individualized listening to music | 2 | 1 | 1 | 2 | 1 | 1 | 1 |
| Rapp | 2018 | Permanent placements in institution from temporary stays | 2 | 2 | 1 | 3 | 3 | 1 | 2 |
| Reimer | 2004 | Special Care Facility Compared with Traditional Environments | 2 | 2 | 1 | 2 | 3 | 1 | 2 |
| Richards | 2019 | Visual arts education | 2 | 1 | 1 | 2 | 1 | 1 | 1 |
| Ridder | 2013 | Individual music therapy | 2 | 1 | 2 | 2 | 1 | 1 | 1 |
| Ridder | 2009 | Music therapy | 3 | 3 | 2 | 3 | 1 | 1 | 3 |
| Rogers | 1998 | Donepezil | 2 | 1 | 1 | 1 | 3 | 2 | 2 |
| Rogers | 1996 | Donepezil | 2 | 1 | 1 | 1 | 3 | 1 | 2 |
| Rogers | 1998 | Donepezil | 2 | 1 | 1 | 1 | 3 | 1 | 2 |
| Rokstad | 2013 | Dementia Care Mapping vs. the VIPS practice model | 2 | 1 | 1 | 2 | 1 | 1 | 1 |
| Rokstad | 2017 | Specialised Day Care centres | 2 | 2 | 1 | 2 | 1 | 1 | 1 |
| Rose | 2020 | Reminiscence therapy | 2 | 2 | 3 | 2 | 1 | 1 | 2 |
| Rubbi | 2016 | Video-music therapy | 3 | 2 | 2 | 2 | 1 | 1 | 2 |
| Saint-Bryant | 2020 | SettleIN, a manualised staff-led programme designed to facilitate adjustment to care for new residents with dementia. | 2 | 1 | 1 | 2 | 1 | 2 | 1 |
| Sakakibara | 2015 | Generic donepezil vs. Aricept | 2 | 2 | 2 | 2 | 1 | 1 | 1 |
| Salazar | 2017 | Hatha Yoga | 2 | 2 | 2 | 2 | 1 | 3 | 2 |
| Sanchez-Valdeon | 2019 | Canine-assisted therapy | 2 | 2 | 1 | 2 | 1 | 1 | 1 |
| Sano | 1992 | Acetyl Levocarnitine | 2 | 1 | 1 | 1 | 3 | 1 | 2 |
| Santos | 2015 | Multidisciplinary rehabilitation program | 2 | 1 | 1 | 2 | 1 | 1 | 1 |
| Sarkamo | 2014 | Regular musical activities | 2 | 1 | 2 | 2 | 1 | 2 | 1 |
| Schall | 2018 | Art museum-based intervention | 2 | 1 | 2 | 2 | 1 | 2 | 1 |
| Schecker | 2013 | ‘Client-centered’ global stimulation vs. cognitive training | 2 | 1 | 1 | 2 | 1 | 2 | 1 |
| Scheltens | 2010 | Medical food | 1 | 1 | 1 | 1 | 1 | 1 | 1 |
| Serrani Azcurra | 2012 | Reminiscence program intervention | 2 | 1 | 2 | 2 | 3 | 1 | 2 |
| Shaw | 2021 | Group-based exercise | 2 | 1 | 1 | 2 | 1 | 1 | 1 |
| Sheehan | 2013 | Referral to general hospital liaison psychiatry services | 2 | 2 | 2 | 3 | 1 | 1 | 2 |
| Shi | 2012 | Acupuncture | 2 | 2 | 1 | 3 | 1 | 1 | 2 |
| Shi | 2015 | Acupuncture | 3 | 2 | 2 | 2 | 1 | 1 | 2 |
| Shoesmith | 2020 | Therapeutic visual art intervention | 2 | 2 | 2 | 3 | 1 | 1 | 2 |
| Silva | 2017 | Memo+ (a paper and pencil memory training program); vs. SenseCam (wearable camera used as a passive external memory aid); vs. written diary (a personal journal, used as control condition) | 2 | 1 | 1 | 2 | 3 | 1 | 2 |
| Silva Serelli | 2017 | Staff training for assisted living residences protocol | 2 | 2 | 2 | 3 | 1 | 1 | 2 |
| Simoncini | 2015 | Acupressure | 2 | 2 | 2 | 3 | 3 | 1 | 3 |
| Slaughter | 2015 | Sit-to-stand activity | 2 | 2 | 1 | 3 | 1 | 1 | 2 |
| Smith | 2012 | Deep Brain Stimulation | 2 | 2 | 3 | 3 | 1 | 1 | 3 |
| Soylemez | 2016 | Progressively Lowered Stress Threshold Model | 2 | 1 | 1 | 2 | 1 | 1 | 1 |
| Spector | 2003 | Cognitive Stimulation Therapy | 2 | 1 | 1 | 2 | 1 | 1 | 1 |
| Stanley | 2013 | Cognitive Behavioural Therapy-based intervention | 2 | 1 | 3 | 2 | 1 | 1 | 2 |
| Steinberg | 2009 | Home-based exercise intervention program | 2 | 1 | 1 | 2 | 1 | 1 | 1 |
| Stewart | 2017 | Cognitive Stimulation Therapy | 3 | 2 | 1 | 3 | 1 | 1 | 3 |
| Streater | 2016 | Cognitive Stimulation Therapy | 2 | 2 | 1 | 3 | 1 | 1 | 2 |
| Subramaniam | 2014 | Life review and life story books | 2 | 1 | 1 | 2 | 1 | 2 | 1 |
| Subramaniam | 2016 | Digital life storybooks | 2 | 3 | 2 | 3 | 1 | 1 | 3 |
| Sultzer | 2008 | Atypical antipsychotic medication (olanzapine, quetiapine, risperidone) | 2 | 1 | 1 | 3 | 1 | 1 | 2 |
| Swinnen | 2021 | Exergaming | 2 | 1 | 1 | 2 | 1 | 1 | 1 |
| Tai | 2016 | Multiple training modalities (Tai-Chi, calligraphy, drawing) | 2 | 2 | 1 | 2 | 1 | 1 | 1 |
| Tamplin | 2018 | Therapeutic group singing | 2 | 2 | 1 | 3 | 1 | 1 | 2 |
| Tanaka | 2017 | Group vs personal rehabilitation sessions | 2 | 1 | 1 | 2 | 1 | 2 | 1 |
| Tay | 2018 | Acute hospital dementia unit (Care for Acute Mentally Infirm Elders [CAMIE]) that adopts a person centred care protocol | 2 | 2 | 1 | 3 | 1 | 1 | 2 |
| Taylor | 2017 | Tailored home-based exercise program | 2 | 2 | 2 | 3 | 1 | 1 | 2 |
| Telenius | 2015 | High-Intensity Exercise Program | 1 | 1 | 1 | 2 | 1 | 1 | 1 |
| Teri | 2020 | Multicomponent exercise plus behavioral/psychosocial intervention (Reducing Disability in Alzheimer’s Disease-NorthWest [RDAD-NW]) | 2 | 2 | 1 | 3 | 1 | 2 | 2 |
| Testad | 2020 | 12-week health promotion course | 2 | 2 | 2 | 3 | 2 | 1 | 2 |
| Thorgrimsen | 2002 | Reminiscence therapy | 2 | 1 | 3 | 2 | 1 | 1 | 2 |
| Tietyen | 2020 | Visual Arts Education pedagogical approach | 2 | 2 | 2 | 3 | 1 | 1 | 2 |
| Todri | 2020 | Global Postural Reeducation | 2 | 1 | 1 | 2 | 1 | 1 | 1 |
| Todri | 2019 | Global Postural Reeducation | 2 | 1 | 2 | 2 | 1 | 1 | 1 |
| Travers | 2013 | Dog-assisted therapy | 2 | 1 | 3 | 2 | 1 | 1 | 2 |
| Travers | 2017 | Behavioral activities intervention (BE-ACTIV) | 3 | 1 | 2 | 3 | 1 | 1 | 3 |
| Uyar | 2019 | Dementia care and support program | 2 | 1 | 1 | 3 | 1 | 1 | 2 |
| Vagenas | 2015 | Rivastigmine transdermal patch | 2 | 2 | 1 | 3 | 1 | 1 | 2 |
| Valenti Soler | 2015 | Social Robots | 2 | 1 | 1 | 2 | 1 | 1 | 1 |
| van Dam | 2020 | Paracetamol | 2 | 1 | 1 | 1 | 1 | 1 | 1 |
| van de Ven-Vakhteeva | 2013 | Antipsychotic use | 2 | 2 | 2 | 3 | 1 | 1 | 2 |
| Van de Ven | 2013 | Dementia Care Mapping (DCM) | 3 | 1 | 2 | 2 | 1 | 2 | 2 |
| van den Elsen | 2015 | Tetrahydrocannabinol | 2 | 1 | 3 | 1 | 1 | 1 | 2 |
| van der Velde-van Buuringen | 2020 | Daily garden use | 2 | 2 | 1 | 3 | 1 | 1 | 2 |
| van Dijk | 2012 | Theatre intervention | 2 | 1 | 2 | 3 | 1 | 1 | 2 |
| van Haeften-van Dijk | 2016 | Community day care with carer support | 2 | 1 | 1 | 3 | 1 | 3 | 3 |
| Veleva | 2020 | Ultraviolet B irradiation vs. Oral vitamin D supplementation | 2 | 1 | 2 | 3 | 1 | 1 | 2 |
| Verbeek | 2010 | Effects of small-scale living facilities on residents | 2 | 1 | 2 | 3 | 1 | 1 | 2 |
| Vigliotti | 2019 | TimeSlips Creative Storytelling Program | 2 | 2 | 2 | 3 | 3 | 1 | 3 |
| Villar | 2019 | Intervention in which people with dementia attended meetings to discuss their individual care plans | 2 | 2 | 2 | 3 | 3 | 1 | 3 |
| Villars | 2015 | Therapeutic Educational Program | 2 | 2 | 2 | 2 | 1 | 1 | 1 |
| Viola | 2011 | Multidisciplinary cognitive rehabilitation program | 2 | 1 | 2 | 2 | 1 | 1 | 1 |
| Vroomen | 2015 | Intensive case management [ICMM] and linkage [LM] models | 2 | 1 | 2 | 2 | 1 | 1 | 1 |
| Walter | 2007 | Humour therapy | 2 | 1 | 1 | 3 | 3 | 1 | 3 |
| Wang | 2018 | Spore powder of Ganoderma lucidum | 3 | 1 | 1 | 1 | 1 | 1 | 2 |
| Ward | 2008 | Outpatient dementia service combined with cholinesterase inhibitor use | 2 | 2 | 2 | 3 | 1 | 1 | 2 |
| Weintraub | 2010 | Sertraline | 2 | 1 | 2 | 1 | 1 | 3 | 2 |
| Wenborn | 2021 | Community Occupational Therapy in Dementia–UK version (COTiD-UK). | 2 | 1 | 1 | 1 | 1 | 1 | 1 |
| Wenborn | 2013 | Occupational Therapy program | 2 | 1 | 2 | 2 | 1 | 1 | 1 |
| Werheid | 2020 | Cognitive Stimulation Therapy | 3 | 2 | 2 | 3 | 1 | 2 | 3 |
| Wilks | 2019 | Montessori based activity program | 3 | 2 | 2 | 3 | 1 | 1 | 3 |
| Wolf-Ostermann | 2012 | Shared-housing arrangements vs. Special Care Units | 1 | 2 | 2 | 3 | 1 | 1 | 2 |
| Wong | 2017 | Cognitive Stimulation Therapy | 2 | 2 | 1 | 3 | 1 | 1 | 2 |
| Woods | 2016 | Joint reminiscence groups - with participants with dementia and carers | 2 | 1 | 1 | 1 | 1 | 1 | 1 |
| Yamanaka | 2013 | Cognitive Stimulation Therapy | 2 | 1 | 2 | 1 | 1 | 1 | 1 |
| Yang | 2015 | Aerobic exercise (cycling intervention) | 2 | 1 | 1 | 2 | 1 | 3 | 2 |
| Yang, B | 2021 | Stressor-oriented multicomponent intervention | 2 | 1 | 1 | 2 | 1 | 1 | 1 |
| Yang, Y | 2021 | Horticultural therapy | 2 | 1 | 3 | 2 | 1 | 1 | 2 |
| Yasuda | 2017 | Care staff training based on person-centered care and dementia care mapping | 2 | 2 | 2 | 3 | 1 | 1 | 2 |
| Yokota | 2006 | Group home-care | 3 | 2 | 2 | 3 | 1 | 1 | 3 |
| Yu | 2013 | Aerobic exercise (cycling intervention) | 2 | 2 | 1 | 2 | 1 | 3 | 2 |
| Yu | 2019 | Mobile reminiscing therapy app | 2 | 1 | 1 | 2 | 3 | 2 | 2 |
| Yue | 2020 | Scalp electroacupuncture combined with memantine | 1 | 2 | 1 | 3 | 1 | 2 | 2 |
| Zhang, S | 2020 | Continuing care combined with music therapy | 3 | 2 | 1 | 3 | 1 | 1 | 3 |
| Zhang, X | 2020 | Rivastigmine hydrogen tartrate combined with donepezil hydrochloride | 3 | 2 | 1 | 3 | 1 | 1 | 3 |
| Zhu | 2020 | 3+1 holistic rehabilitation nursing mode | 3 | 2 | 1 | 3 | 1 | 1 | 3 |
| Zongfang | 2020 | Piracetam with Nimodipine | 3 | 2 | 1 | 3 | 1 | 1 | 3 |

The quality of each paper was evaluated using the Quality Assessment Tool for Quantitative Studies^1^, developed by the Effective Public Healthcare Panacea Project in Canada. Each study was assessed using the following domains, giving a score of strong (1), moderate (2) or weak (3) for each: selection bias, study design, confounders, blinding, data collection method, withdrawals and dropouts. Papers with no weak ratings were given a global rating of strong (1), those with one weak rating were given a global rating of moderate (2) and those with two or more weak ratings were given a global rating of weak (3). The quality of case reports and case series were assessed by the CARE case report guidelines^2^.

**References**

1 Quality Assessment Tool for Quantitative Studies: Effective Public Healthcare Panacea Project; [Available from: <https://www.ephpp.ca/quality-assessment-tool-for-quantitative-studies/>.

2 Gagnier JJ, Kienle G, Altman DG, Moher D, Sox H, Riley D. The CARE Guidelines: Consensus-based Clinical Case Reporting Guideline Development.: the CARE Group.
