# Supplementary material for: Systematic review of the efficacy of pharmacological and non-pharmacological interventions for improving quality of life of people with dementia
Source: Br J Psychiatry. 2025 Apr 1;228(1):55–67. doi: 10.1192/bjp.2025.11 (PMC12722012; doi:10.1192/bjp.2025.11)
Supplement: Luxton et al. supplementary material 8 — Luxton et al. supplementary material [file S000712502500011Xsup008.docx]

| **First Author & intervention studied** | **1** | **2** | **3a** | **3b** | **3c** | **3d** | **4** | **5a** | **5b** | **5c** | **5d** | **6** | **7** | **8a** | **8b** | **8c** | **8d** | **9a** | **9b** | **9c** | **10a** | **10b** | **10c** | **10d** | **11a** | **11b** | **11c** | **11d** | **12** | **13** |
| --- | --- | --- | --- | --- | --- | --- | --- | --- | --- | --- | --- | --- | --- | --- | --- | --- | --- | --- | --- | --- | --- | --- | --- | --- | --- | --- | --- | --- | --- | --- |
| Foloppe:  Virtual reality-based training. | Y | N | N | Y | Y | N | Y | Y | Y | Y | N | N | Y | Y | N | Y | N | Y | Y | N | Y | Y | N | N | N | Y | Y | Y | Y | N |
| Graff:  Occupational Therapy program. | Y | N | N | Y | Y | Y | Y | Y | Y | Y | Y | N | N | N | N | Y | N | Y | Y | N | Y | Y | N | N | Y | Y | Y | Y | N | Y |
| Lassell:  Adaptive gardening and adaptive riding. | N | N | N | Y | Y | Y | Y | Y | N | N | N | N | N | N | N | Y | N | Y | Y | N | Y | Y | N | N | Y | Y | Y | Y | N | Y |
| Nakanishi:  Instrumental Activities of Daily Living habituation. | N | N | N | Y | Y | Y | Y | Y | Y | Y | Y | N | N | Y | N | Y | N | Y | Y | N | Y | Y | N | N | Y | Y | Y | Y | N | Y |
| Raglio:  Global music approach to dementia. | Y | N | N | N | N | N | N | Y | N | N | Y | N | N | N | N | Y | N | Y | Y | N | Y | Y | Y | N | N | N | Y | Y | N | Y |

**Supplementary material-6:** Quality assessment of case reports and case series included in review using the CARE checklist^1^

Y= Yes; N=No; 1= The diagnosis or intervention of primary focus followed by the words “case report”; 2= Keywords; 3a= What is unique about the case and what does it add to the scientific literature?; 3b= The patient’s main concerns and important clinical findings; 3c= the primary diagnoses, interventions, and outcomes; 3d= “take-away” lessons of the case report (abstract); 4= Briefly summarizes why this case is unique; 5a= De-identified patient specific information; 5b= Primary concerns and symptoms of the patient; 5c= Medical, family, and psychosocial history including relevant genetic information; 5d= Relevant past interventions and their outcomes; 6= Describe significant physical examination and important clinical findings; 7= Historical and current information from this episode of care organised as a timeline; 8a= Diagnostic methods, 8b= Diagnostic challenges; 8c= Diagnosis; 8d= prognosis; 9a= Type of therapeutic intervention; 9b= dosage, strength, duration of intervention; 9c= changes in intervention and their rationale; 10a= assessed outcomes; 10b= follow-up diagnostic tests/ other tests; 10c= Intervention adherence and tolerability; 10d= Adverse effects; 11a= Strengths and limitations; 11b= Discussion of the relevant medical literature; 11c= rationale for conclusions; 11d= key take-away message in conclusion; 12= patient perspective; 13= informed consent from the patient.

**References:**

1 Riley DS, Barber MS, Kienle GS, Aronson JK, von Schoen-Angerer T, Tugwell P, *et al.* CARE guidelines for case reports: explanation and elaboration document. *J Clin Epidemiol* 2017; **89**: 218–35.
