# Supplementary material for: Systematic review of the efficacy of pharmacological and non-pharmacological interventions for improving quality of life of people with dementia
Source: Br J Psychiatry. 2025 Apr 1;228(1):55–67. doi: 10.1192/bjp.2025.11 (PMC12722012; doi:10.1192/bjp.2025.11)
Supplement: Luxton et al. supplementary material 9 — Luxton et al. supplementary material [file S000712502500011Xsup009.docx]

**Supplementary material-7:** Non-pharmacological interventions that have level 3 evidence for improving quality of life of people with dementia^1,2^.

| **Intervention** | **GRADE certainty rating** |
| --- | --- |
| Time-limited early-stage memory loss support group program^3^ | High |
| Comprehensive nursing intervention^4^ | Moderate |
| Intensive occupational therapy centred program^5^ | Moderate |
| Multidisciplinary rehabilitation program^6^ | Moderate |
| Nursing patients in street clothes^7^ | Moderate |
| Scalp electroacupuncture^8^ | Moderate |
| Specialised Day Care centres^9^ | Moderate |
| 3+1 holistic rehabilitation nursing mode^10^ | Low |
| Aerobic walking exercise training and upper limbs exercises^11^ | Low |
| Art museum-based intervention^12^ | Low |
| Involving institutionalised people with dementia in their care-planning meetings^13^ | Low |
| Personalised multi-domain intervention^14^ | Low |
| Person Centred Care model within an acute hospital dementia unit^15^ | Low |
| Training intervention for homecare workers^16^ | Low |

GRADE of certainty ratings^1^: High = the true effect is similar to the estimated effect, Moderate = the true effect is probably close to the estimated effect; Low = the true effect may be markedly different from the estimated effect; Very Low = the true effect is probably markedly different from the estimated effect.

**References:**

1 Guyatt GH, Oxman AD, Vist GE, Kunz R, Falck-Ytter Y, Alonso-Coello P, *et al.* GRADE: an emerging consensus on rating quality of evidence and strength of recommendations. *BMJ* 2008; **336**: 924–6.

2 OCEBM Levels of Evidence Working Group. The Oxford 2011 Levels of Evidence. Oxford Centre for Evidence-Based Medicine.

3 Logsdon RG, Pike KC, McCurry SM, Hunter P, Maher J, Snyder L, *et al.* Early-stage memory loss support groups: outcomes from a randomized controlled clinical trial. *J Gerontol B Psychol Sci Soc Sci* 2010; **65**: 691–7.

4 Liu SY, Shen YY, Zheng GF. Application of comprehensive nursing intervention for Alzheimer’s patients and its effects on recovery of cognitive function. *Int J Clin Exp Med* 2019; **12**: 4012–9.

5 Kim KU, Kim SH, Oh HW. The effects of occupation-centered activity program on fall-related factors and quality of life in patients with dementia. *J Phys Ther Sci* 2017; **29**: 1188–91.

6 Santos GD, Nunes P v, Stella F, Brum PS, Yassuda MS, Ueno LM, *et al.* Multidisciplinary rehabilitation program: Effects of a multimodal intervention for patients with Alzheimer’s disease and cognitive impairment without dementia. *Rev Psiquiatr Clín* 2015; **42**: 153–6.

7 Charras K, Gzil F. Judging a Book by Its Cover: Uniforms and Quality of Life in Special Care Units for People With Dementia. *Am J Alzheimers Dis Other Demen* 2013; **28**: 450–8.

8 Yue A, Han X, Mao E, Wu G, Gao J, Huang L, *et al.* The effect of scalp electroacupuncture combined with Memantine in patients with vascular dementia: A retrospective study. *Medicine* 2020; **99**: e21242.

9 Rokstad AMM, Engedal K, Kirkevold Ø, Šaltytė Benth J, Barca ML, Selbæk G, *et al.* The association between attending specialized day care centers and the quality of life of people with dementia. *Int Psychogeriatr* 2017; **29**: 627–36.

10 Zhu B, Yao QP, Shao ZM, Sun LL, Ruan HL, Wang GY, *et al.* Effects of 3+1 holistic rehabilitation nursing mode on the rehabilitation and cognitive function of patients with Alzheimer’s disease. *Int J Clin Exp Med* 2020; **13**: 5645–52.

11 Abd El- Kader SM. Role of Aerobic Exercise Training in Changing Exercise Tolerance and Quality of Life in Alzheimer’s Disease. *Eur J Gen Med* 2011; **8**: 1–6.

12 Schall A, Tesky VA, Adams AK, Pantel J. Art museum-based intervention to promote emotional well-being and improve quality of life in people with dementia: The ARTEMIS project. *Dementia* 2018; **17**: 728–43.

13 Villar F, Celdran M, Vila-Miravent J, Fernandez E. Involving institutionalized people with dementia in their care-planning meetings: Impact on their quality of life measured by a proxy method: Innovative Practice. *Dementia* 2019; **18**: 1936–41.

14 Koskas P, Kohler S, Estrada J, Sebbagh M, Lacaille S, Lilamand M. Effect of a multi-domain intervention on the quality of life in older adults with major neurocognitive disorder: A pilot study. *Revue Neurologique* 2022; **178**(4): 355-62.

15. Tay FHE, Thompson CL, Nieh CM, Nieh CC, Koh HM, Tan JJC, *et al.* Person-centered care for older people with dementia in the acute hospital. *Alzheimers Dement (N Y)* 2018; **4**: 19–27.

16. Cooper C, Zabihi S, Akhtar A, Lee T, Isaaq A, Le Novere M, et al. Feasibility and acceptability of NIDUS-professional, a training and support intervention for homecare workers caring for clients living with dementia: a cluster-randomised feasibility trial. *Age & Ageing* 2024; **53**(4): 01
