# Supplementary material for: Systematic review of the efficacy of pharmacological and non-pharmacological interventions for improving quality of life of people with dementia
Source: Br J Psychiatry. 2025 Apr 1;228(1):55–67. doi: 10.1192/bjp.2025.11 (PMC12722012; doi:10.1192/bjp.2025.11)
Supplement: Luxton et al. supplementary material 10 — Luxton et al. supplementary material [file S000712502500011Xsup010.docx]

**Supplementary material-8:** Non-pharmacological interventions that have level 4 evidence for improving quality of life of people with dementia^1,2^.

| **Intervention** | **GRADE certainty rating** |
| --- | --- |
| Acupuncture^3^ | Moderate |
| Multicomponent exercise plus psychosocial intervention^4^ | Moderate |
| Preventing Loss of Independence through Exercise program^5^ | Moderate |
| 12-week health promotion course^6^ | Low |
| Canine-assisted therapy^7^ | Low |
| Care staff training based on person-centered care and dementia care mapping^8^ | Low |
| Communication intervention^9^ | Low |
| Dog-assisted intervention in nursing homes^10^ | Low |
| Compassion-focused therapy group^11^ | Low |
| Elder-clowning^12^ | Low |
| Group-based singing^13^ | Low |
| Integrated palliative homecare programme^14^ | Low |
| Intergenerational reminiscence programme^15^ | Low |
| Multifaceted walking intervention^16^ | Low |
| Namaste Care intervention^17^ | Low |
| Permanent placement in institution^18^ | Low |
| Person-centred creative dance intervention^19^ | Low |
| Rehabilitation program consisting of CST^20^ | Low |
| Reminiscence music with mobile app (Memory Tracks)^21^ | Low |
| Remotely Delivered Exercise Sessions^22^ | Low |
| Therapeutic Educational Program^23^ | Low |
| ‘‘Living Well with Dementia’’ (LivDem) model of group support^24^ | Very low |
| ‘Dancing down memory lane’: Circle dancing psychotherapeutic intervention^25^ | Very low |
| Affordable robotic pet ownership^26^ | Very low |
| Cognitive-behavioural intervention program^27^ | Very low |
| Computer interactive reminiscence and conversation aid groups^28^ | Very low |
| Digital life storybooks^29^ | Very low |
| Dignity Therapy^30^ | Very low |
| Emisymmetric bilateral stimulation^31^ | Very low |
| Experience-based group therapy^32^ | Very low |
| Global Music Approach to Dementia intervention^33^ | Very low |
| Group reminiscence therapy^34^ | Very low |
| High daily light exposure^35^ | Very low |
| Indoor sensory garden^36^ | Very low |
| Instrumental Activities of Daily Living habituation^37^ | Very low |
| Multicomponent rehabilitation programme^38^ | Very low |
| Music therapy^4^ | Very low |
| Robotic cat intervention^39^ | Very low |
| Therapeutic garden^40^ | Very low |
| Therapeutic visual art intervention^41^ | Very low |
| Video-music therapy^42^ | Very low |
| 4-week music therapy course^43^ | Very low |

GRADE of certainty ratings^1^: High = the true effect is similar to the estimated effect, Moderate = the true effect is probably close to the estimated effect; Low = the true effect may be markedly different from the estimated effect; Very Low = the true effect is probably markedly different from the estimated effect.

**References:**

1 Guyatt GH, Oxman AD, Vist GE, Kunz R, Falck-Ytter Y, Alonso-Coello P, *et al.* GRADE: an emerging consensus on rating quality of evidence and strength of recommendations. *BMJ* 2008; **336**: 924–6.

2 OCEBM Levels of Evidence Working Group. The Oxford 2011 Levels of Evidence. Oxford Centre for Evidence-Based Medicine.

3 Shi GX, Liu CZ, Li QQ, Zhu H, Wang LP. Influence of acupuncture on cognitive function and markers of oxidative DNA damage in patients with vascular dementia. *Journal of Traditional Chinese Medicine* 2012; **32**: 199–202.

4 Teri L, Logsdon RG, McCurry SM, Pike KC, McGough EL. Translating an Evidence-based Multicomponent Intervention for Older Adults With Dementia and Caregivers. *Gerontologist* 2020; **60**: 548–57.

5 Mehling WE, Scott TM, Duffy J, Whitmer RA, Chesney MA, Boscardin WJ, *et al.* Dyadic Group Exercises for Persons with Memory Deficits and Care Partners: Mixed-Method Findings from the Paired Preventing Loss of Independence through Exercise (PLIE) Randomized Trial. *Journal of Alzheimer’s Disease* 2020; **78**: 1689–706.

6 Testad I, Kajander M, Gjestsen MT, Dalen I. Health promotion intervention for people with early-stage dementia: A quasi-experimental study. *Brain Behav* 2020; **10**: e01888.

7 Nordgren L, Engstrom G. Animal-assisted intervention in dementia: effects on quality of life. *Clin Nurs Res* 2014; **23**: 7–19.

8 Yasuda M, Sakakibara H. Care staff training based on person-centered care and dementia care mapping, and its effects on the quality of life of nursing home residents with dementia. *Aging Ment Health* 2017; **21**: 991–6.

9 McGilton KS, Rochon E, Sidani S, Shaw A, Ben-David BM, Saragosa M, *et al.* Can We Help Care Providers Communicate More Effectively With Persons Having Dementia Living in Long-Term Care Homes? *Am J Alzheimers Dis Other Demen* 2017; **32**: 41–50.

10 Karefjard A, Nordgren L. Effects of dog-assisted intervention on quality of life in nursing home residents with dementia. *Scand J Occup Ther* 2019; **26**: 433–40.

11 Collins RN, Gilligan LJ, Poz R. The Evaluation of a Compassion-Focused Therapy Group for Couples Experiencing a Dementia Diagnosis. *Clin Gerontol* 2018; **41**: 474–86.

12 Kontos P, Miller KL, Colobong R, Palma Lazgare LI, Binns M, Low LF, *et al.* Elder-Clowning in Long-Term Dementia Care: Results of a Pilot Study. *J Am Geriatr Soc* 2016; **64**: 347–53.

13 Moir AR, Cassidy-Nolan D, Gough AS, Cassidy K-L. Music Therapy in Long-Term Care: Impact on Behavioural and Psychological Symptoms of Dementia and Facility Milieu. *Canadian Journal of Music Therapy* 2019; **25**: 50–9.

14 Hum A, Tay RY, Wong YKY, Ali NB, Leong IYO, Wu HY, *et al.* Advanced dementia: an integrated homecare programme. *BMJ Support Palliat Care* 2020; **10**: e40.

15 Chung JCC. An intergenerational reminiscence programme for older adults with early dementia and youth volunteers: values and challenges. *Scand J Caring Sci* 2009; **23**: 259–64.

16 Chu CH, Puts M, Brooks D, Parry M, McGilton KS. A Feasibility Study of a Multifaceted Walking Intervention to Maintain the Functional Mobility, Activities of Daily Living, and Quality of Life of Nursing Home Residents With Dementia. *Rehabilitation Nursing Journal* 2020; **45**: 204–17.

17 Latham I, Brooker D, Bray J, Jacobson-Wright N, Frost F. The Impact of Implementing a Namaste Care Intervention in UK Care Homes for People Living with Advanced Dementia, Staff and Families. *International Journal of Environmental Research & Public Health [Electronic Resource]* 2020; **17**: 18.

18 Rapp T, Apouey BH, Senik C. The impact of institution use on the wellbeing of Alzheimer’s disease patients and their caregivers. *Soc Sci Med* 2018; **207**: 1–10.

19 Koh WLE, Low F, Kam JW, Rahim S, Ng WF, Ng L. Person-centred creative dance intervention for persons with dementia living in the community in Singapore. *Dementia-International Journal of Social Research and Practice* 2020; **19**: 2430–43.

20 Ferrer B, del Valle A. A Rehabilitation Program for Alzheimer’s Disease. *Journal of Nursing Research* 2014; **22**: 192–9.

21 Cunningham S, Brill M, Whalley JH, Read R, Anderson G, Edwards S, *et al.* Assessing Wellbeing in People Living with Dementia Using Reminiscence Music with a Mobile App (Memory Tracks): A Mixed Methods Cohort Study. *J Healthc Eng* 2019; **2019**: 8924273.

22 Ptomey LT, Vidoni ED, Montenegro-Montenegro E, Thompson MA, Sherman JR, Gorczyca AM, *et al.* The Feasibility of Remotely Delivered Exercise Session in Adults With Alzheimer’s Disease and Their Caregivers. *J Aging Phys Act* 2019; **27**: 670–7.

23 Villars H, Dupuy C, Perrin A, Vellas B, Nourhashemi F. Impact of a therapeutic educational program on quality of life in Alzheimer’s disease: results of a pilot study. *Journal of Alzheimer’s Disease* 2015; **43**: 167–76.

24 Cheston R, Howells L. A feasibility study of translating ‘Living Well with Dementia’ groups into a Primary Care Improving Access to Psychological Therapy service (innovative practice). *Dementia* 2016; **15**: 273–8.

25 Hamill M, Smith L, Röhricht F. ‘Dancing down memory lane’: Circle dancing as a psychotherapeutic intervention in dementia—a pilot study. *Dementia (14713012)* 2012; **11**: 709–24.

26 Hammarlund RA, Whatley KL, Zielinski MH, Jubert JC. Benefits of Affordable Robotic Pet Ownership in Older Adults With Dementia. *J Gerontol Nurs* 2021; **47**: 18–22.

27 Fialho PPA, Koenig AM, dos Santos MDL, Barbosa MT, Caramelli P. Positive effects of a cognitive-behavioral intervention program for family caregivers of demented elderly. *Arq Neuropsiquiatr* 2012; **70**: 786–92.

28 Astell AJ, Smith SK, Potter S, Preston-Jones E. Computer Interactive Reminiscence and Conversation Aid groups—Delivering cognitive stimulation with technology. *Alzheimer’s and Dementia: Translational Research and Clinical Interventions* 2018; **4**: 481–7.

29 Subramaniam P, Woods B. Digital life storybooks for people with dementia living in care homes: an evaluation. *Clin Interv Aging* 2016; **11**: 1263–76.

30 Johnston B, Lawton S, McCaw C, Law E, Murray J, Gibb J, *et al.* Living well with dementia: enhancing dignity and quality of life, using a novel intervention, Dignity Therapy. *Int J Older People Nurs* 2016; **11**: 107–20.

31 Guerriero F, Botarelli E, Mele G, Polo L, Zoncu D, Renati P, *et al.* An innovative intervention for the treatment of cognitive impairment–emisymmetric bilateral stimulation improves cognitive functions in alzheimer’s disease and mild cognitive impairment: An open-label study. *Neuropsychiatr Dis Treat* 2015; **11**: 2391–404.

32 Kim HH. Effects of experience-based group therapy on cognitive and physical functions and psychological symptoms of elderly people with mild dementia. *J Phys Ther Sci* 2015; **27**: 2069–71.

33 Raglio A, Filippi S, Leonardelli L, Trentini E, Bellandi D. The Global Music Approach to Dementia (GMA-D): evidences from a case report. *Aging-Clinical & Experimental Research* 2018; **30**: 1533–6.

34 Jo HK, Song E. The Effect of Reminiscence Therapy on Depression, Quality of Life, Ego-Integrity, Social Behavior Function, and Activies of Daily Living in Elderly Patients With Mild Dementia. *Educ Gerontol* 2015; **41**: 1–13.

35 Munch M, Schmieder M, Bieler K, Goldbach R, Fuhrmann T, Zumstein N, *et al.* Bright Light Delights: Effects of Daily Light Exposure on Emotions, Restactivity Cycles, Sleep and Melatonin Secretion in Severely Demented Patients. *Curr Alzheimer Res* 2017; **14**: 1063–75.

36 Collins H, van Puymbroeck M, Hawkins BL, Vidotto J. The Impact of a Sensory Garden for People with Dementia. *Ther Recreation J* 2020; **54**: 48–63.

37 Nakanishi K, Yamaga T. Effect of Instrumental Activities of Daily Living habituation due to routinising therapy in patients with frontotemporal dementia. *BMJ Case Rep* 2021; **14**: 4.

38 Cornelis E, Gorus E, Beyer I, van Puyvelde K, Lieten S, Versijpt J, *et al.* A retrospective study of a multicomponent rehabilitation programme for community-dwelling persons with dementia and their caregivers. *British Journal of Occupational Therapy* 2018; **81**: 5–14.

39 Gustafsson C, Svanberg C, Mullersdorf M. Using a Robotic Cat in Dementia Care: A Pilot Study. *J Gerontol Nurs* 2015; **41**: 46–56.

40 Edwards CA, McDonnell C, Merl H. An evaluation of a therapeutic garden’s influence on the quality of life of aged care residents with dementia. *Dementia* 2013; **12**: 494–510.

41 Shoesmith E, Charura D, Surr C. Acceptability and feasibility study of a six-week person-centred, therapeutic visual art intervention for people with dementia. *Arts Health* 2020; : 1–19.

42 Rubbi I, Magnani D, Naldoni G, di Lorenzo R, Cremonini V, Capucci P, *et al.* Efficacy of video-music therapy on quality of life improvement in a group of patients with Alzheimer’s disease: a pre-post study. *Acta Bio-Medica de l Ateneo Parmense* 2016; **87**: 30–7.

43 Ridder HM, Wigram T, Ottesen AM. A pilot study on the effects of music therapy on frontotemporal dementia -- developing a research protocol. *Nord J Music Ther* 2009; **18**: 103–32.
