# Supplementary figures and images for: Systematic review of the efficacy of pharmacological and non-pharmacological interventions for improving quality of life of people with dementia
Source: Br J Psychiatry. 2025 Apr 1;228(1):55–67. doi: 10.1192/bjp.2025.11 (PMC12722012; doi:10.1192/bjp.2025.11)

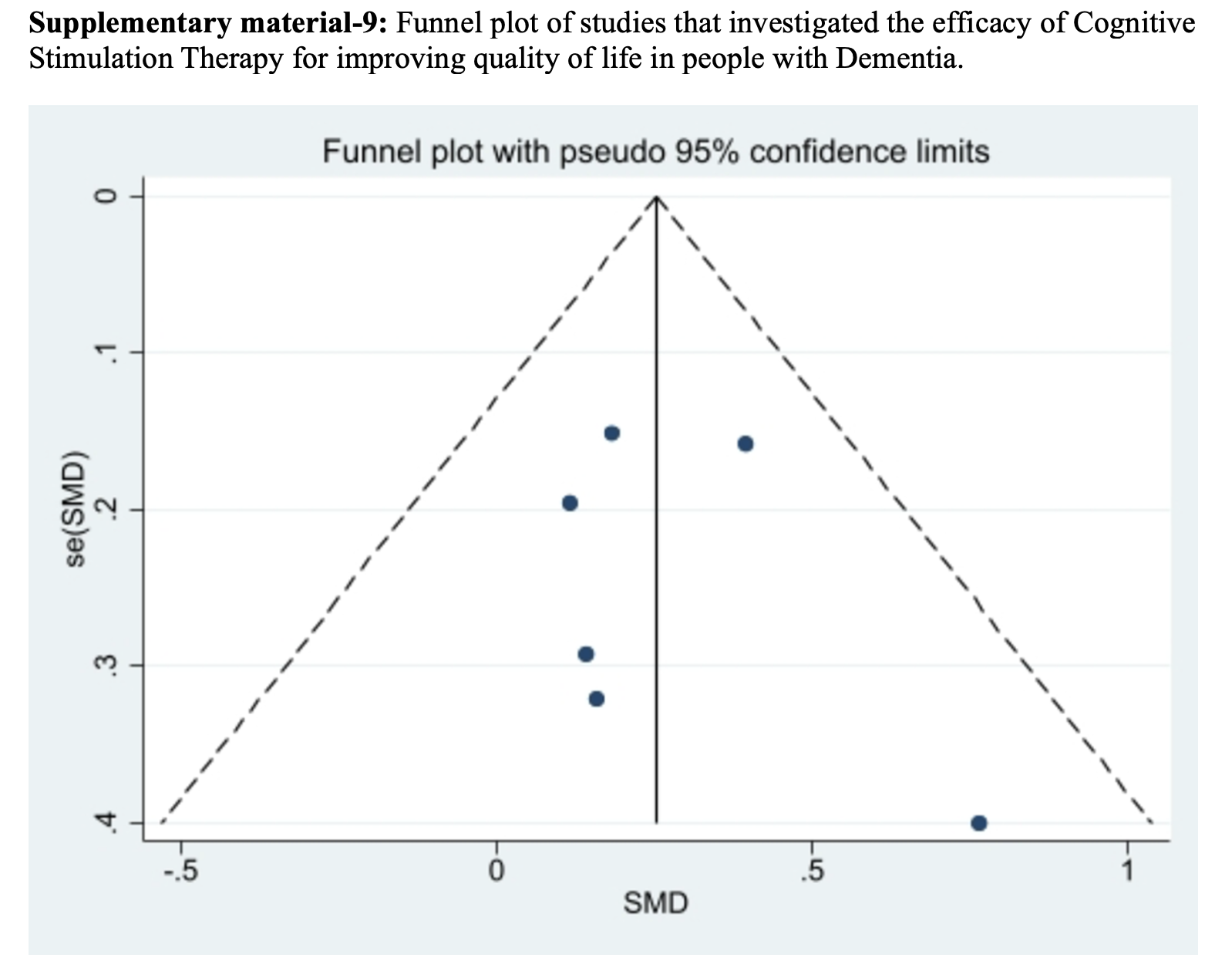

Supplement: Luxton et al. supplementary material 11 — Luxton et al. supplementary material [file S000712502500011Xsup011.zip › Supplementary material-9.png]
